# mrMLM v4.0.2: An R Platform for Multi-locus Genome-wide Association Studies

**DOI:** 10.1016/j.gpb.2020.06.006

**Published:** 2020-12-18

**Authors:** Ya-Wen Zhang, Cox Lwaka Tamba, Yang-Jun Wen, Pei Li, Wen-Long Ren, Yuan-Li Ni, Jun Gao, Yuan-Ming Zhang

**Affiliations:** 1 Crop Information Center, College of Plant Science and Technology, Huazhong Agricultural University, Wuhan 430070, China; 2 Department of Mathematics, Egerton University, Egerton 536-20115, Kenya; 3 State Key Laboratory of Crop Genetics and Germplasm Enhancement, Nanjing Agricultural University, Nanjing 210095, China; 4 Department of Epidemiology and Medical Statistics, School of Public Health, Nantong University, Nantong 226019, China; 5 College of Informatics, Huazhong Agricultural University, Wuhan 430070, China

**Keywords:** Genome-wide association study, Linear mixed model, mrMLM, Multi-locus genetic model, R

## Abstract

Previous studies have reported that some important loci are missed in single-locus **genome-wide association studies** (GWAS), especially because of the large phenotypic error in field experiments. To solve this issue, multi-locus GWAS methods have been recommended. However, only a few software packages for multi-locus GWAS are available. Therefore, we developed an **R** software named **mrMLM** v4.0.2. This software integrates mrMLM, FASTmrMLM, FASTmrEMMA, pLARmEB, pKWmEB, and ISIS EM-BLASSO methods developed by our lab. There are four components in mrMLM v4.0.2, including dataset input, parameter setting, software running, and result output. The *fread* function in data.table is used to quickly read datasets, especially big datasets, and the doParallel package is used to conduct parallel computation using multiple CPUs. In addition, the graphical user interface software mrMLM.GUI v4.0.2, built upon Shiny, is also available. To confirm the correctness of the aforementioned programs, all the methods in mrMLM v4.0.2 and three widely-used methods were used to analyze real and simulated datasets. The results confirm the superior performance of mrMLM v4.0.2 to other methods currently available. False positive rates are effectively controlled, albeit with a less stringent significance threshold. mrMLM v4.0.2 is publicly available at BioCode (https://bigd.big.ac.cn/biocode/tools/BT007077) or R (https://cran.r-project.org/web/packages/mrMLM.GUI/index.html) as an open-source software.

## Introduction

Since the establishment of the mixed linear model (MLM) framework of genome-wide association studies (GWAS) [1], [2], the MLM-based GWAS methodologies have been widely used to identify many important loci for complex traits in animals, plants, and humans. With the technological advances in molecular biology, a huge number of markers are easily obtained. However, this brings new computational and analytic challenges. The MLM-based single-marker association in genome-wide scans proves its feasibility. To increase statistical power and decrease running time in quantitative trait nucleotide (QTN) detection, a series of additional MLM-based methods have been proposed. For example, Kang et al. [3] proposed an efficient mixed model association (EMMA), which was then extended to generate EMMAX [4] and GEMMA [5]. Meanwhile, Zhang et al. [6] reported a compressed MLM (CMLM) method, which was then extended to develop ECMLM [7] and SUPER [8]. In addition, other methods have also been developed, *e.g.*, GRAMMAR-Gamma [9], FaST-LMM [10], FaST-LMM-Select [11], and BOLT-LMM [12]. All the aforementioned methods have been subjected to multiple testing. To control the false positive rate in such tests, the Bonferroni correction is frequently adopted. However, this correction is often too conservative to detect many important loci.

To detect more QTNs with a low false positive rate, multi-locus methods have been recommended. This recommendation was implemented for the first time by Segura and his colleagues [13]. Thereafter, Liu et al. [14] developed FarmCPU. Based on the advantages of the random model of QTN effect over the fixed model [15], we have recently developed six multi-locus methods: mrMLM [16], FASTmrMLM [17] (File S1), FASTmrEMMA [18], ISIS EM-BLASSO [19], pLARmEB [20], and pKWmEB [21] (File S2). These methods include two stages. First, various algorithms are used to select all the potentially associated markers. Second, the selected markers are put in one model, then all the effects in this model are estimated by empirical Bayes, and all the non-zero effects are further identified by likelihood ratio test for true QTNs. Although a less stringent significance threshold is adopted, these methods have high power and accuracy, and a low false positive rate.

Many packages are available in the GWAS software, *e.g.*, PLINK [22], TASSEL [23], EMMA [3], EMMAX [4], GEMMA [5], and GAPIT [24], [25] (File S2). However, these packages are almost all based on single-marker association in genome scans. To popularize our multi-locus GWAS methods, we integrated all the six multi-locus approaches into one R package named mrMLM v4.0.2 (Figure S1).

## Implementation

**Figure 1:**
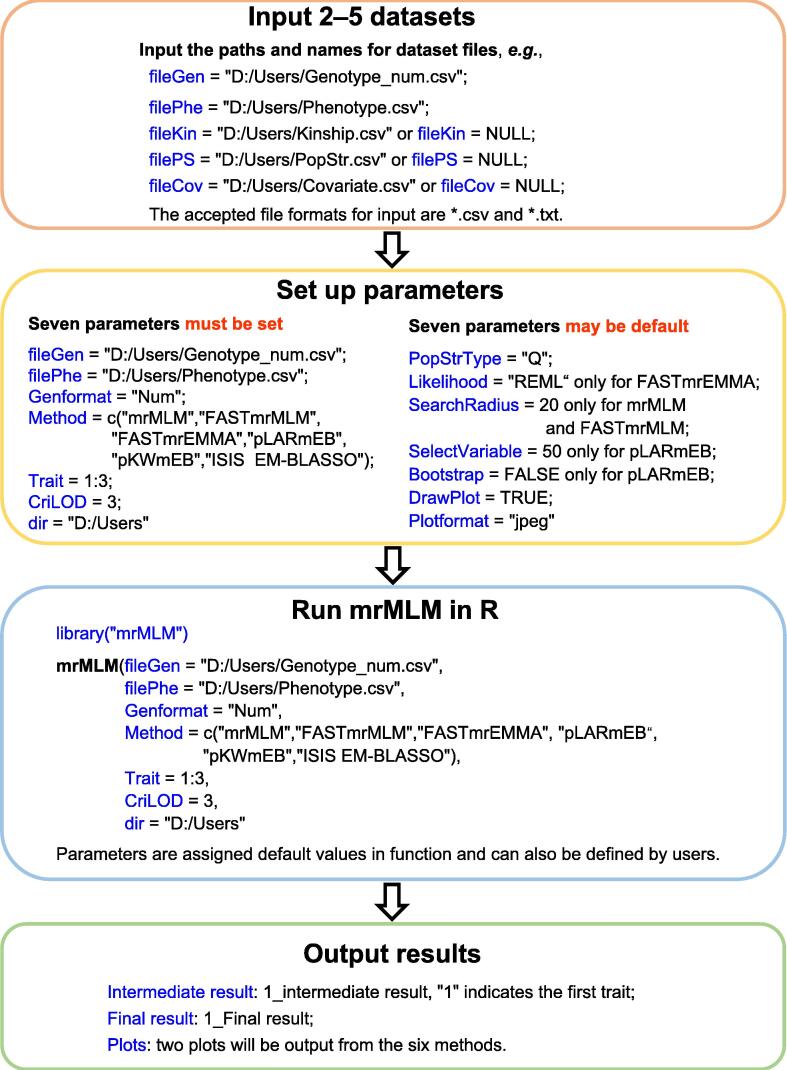
The framework of mrMLM v4.0.2

mrMLM v4.0.2 includes four parts (Figure 1): dataset input, parameter setting, software running, and result output. In the dataset input module, users need to input trait phenotypes and marker genotypes. The two types of datasets are input by the filePhe and fileGen files, respectively, and the available file formats are *.csv and *.txt. Marker genotypes may be indicated by mrMLM numeric (or character) and Hapmap formats, and are used to calculate both kinship (using mrMLM or EMMA [3]) and population structure (using Structure [26] or fastSTRUCTURE [27]) matrices. This software also has an option to input kinship matrix, population structure matrix, and covariate table. The three types of datasets are input by the fileKin, filePS, and fileCov files, respectively. In the parameter setting module, users need to set 17 parameters. Among these parameters, Likelihood, SearchRadius, and SelectVariable are specific to method. Seven parameters may be default or set by users. fileGen, filePhe, Genformat, Method, Trait, CriLOD, and dir must be set by users. In the software running module, users need to use two commands: library(“mrMLM”) and mrMLM(…). In the result output module, intermediate and final results and two plots (*.png, *.tiff, *.jpeg, and *.pdf) are output to the path that users have previously set, *i.e.*, dir=“D:/Users”. The software is started in a computer or server via the codes below (File S3):

mrMLM(fileGen=“D:/Users/Genotype_num.csv”,filePhe=“D:/Users/Phenotype.csv”,fileKin=“D:/Users/Kinship.csv”,filePS=“D:/Users/PopStr.csv”,PopStrType=“Q”,fileCov=“D:/Users/Covariate.csv”,Genformat=“Num”,Method = c(“mrMLM”,“FASTmrMLM”,“FASTmrEMMA”,“pLARmEB”,“pKWmEB”,“ISIS EM-BLASSO”),Likelihood= “REML”,Trait = 1:3,SearchRadius = 20,CriLOD = 3,SelectVariable = 50,Bootstrap = FALSE,DrawPlot = FALSE,Plotformat=“jpeg”,dir=“D:/Users”)

R core is a single-threaded program, and its computing mode limits its ability to handle large-scale data. In mrMLM v4.0.2, however, several R packages were used to perform parallel calculation. First, *detectCores*() and *makeCluster*(cl.cores) in a parallel package were used, respectively, to detect the number of CPUs on the current host and create a set of copies of R running in parallel and communicating over sockets. Then, *registerDoParallel*(cl) in doParallel package was used to register the parallel backend with the foreach package. Third, ‘for’ loop was replaced by *foreach*(i = 1:n,.combine='rbind')%dopar%{…} in foreach package. Finally, *stopCluster*(cl) in parallel package was used to stop the aforementioned parallel calculation.

*fread* function in data.table is used to quickly read datasets, especially big datasets. For reading one genetic dataset with 500 individuals and one million markers, *fread* was three times faster (72.84 s) than *read.csv* (201.45 s). Meanwhile, we utilized the advantages of package bigmemory, which can create, store, access, and manipulate massive matrices, to define the huge genotypic matrix with the aid of the *big.matrix*() function. This largely saves the running time, especially for massive genetic matrix.

The graphical user interface (GUI) software mrMLM.GUI v4.0.2, built upon Shiny, is available as well. The interactive GUI is started via the two commands “*library*(mrMLM.GUI)” and “*mrMLM.GUI*()” (File S4). The next operation can be done through clicking the mouse conveniently.

## Results

To test the performance of the software package mrMLM v4.0.2, three real datasets in rice [28], maize [29], and Simmental beef cattle [30] were downloaded from the Rice SNP-Seek Database (http://snp-seek.irri.org./_download.zul), the Maizego (http://www.maizego.org/Resources.html), and the Dryad Digital Repository (https://datadryad.org/stash/dataset/doi:10.5061/dryad.4qc06), respectively (File S5). In the aforementioned three datasets, the traits of interest are grain width, oil concentration, and kidney weight, respectively; the numbers of phenotypic accessions are 2262, 368, and 1136, respectively; the numbers of markers are 1.01, 1.06, and 0.67 million, respectively (File S5).

### Influence of various factors on QTN detection using mrMLM v4.0.2

**Figure 2:**
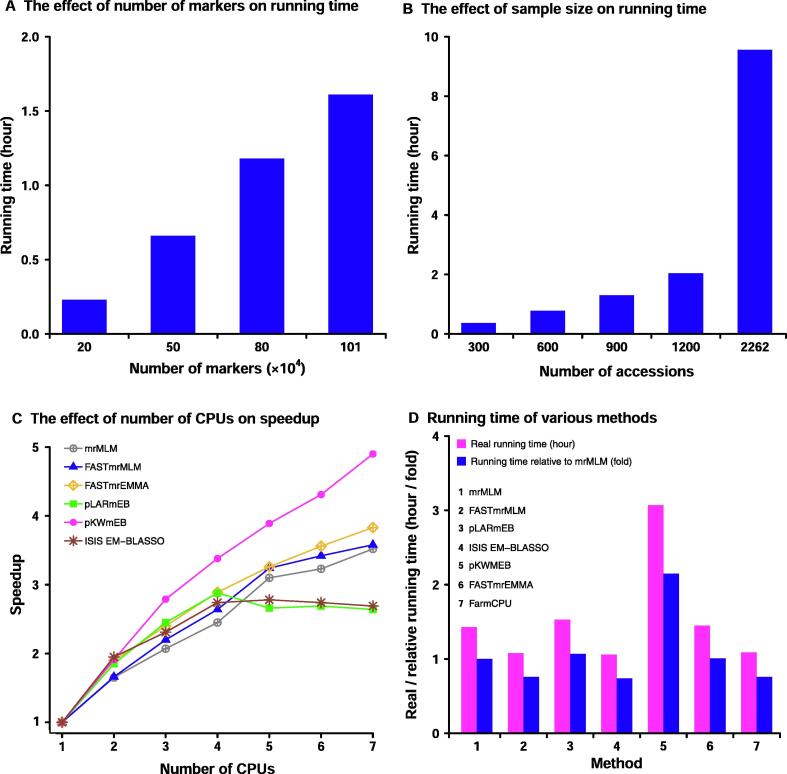
Performance of mrMLM v4.0.2 in detecting QTNs for rice grain width The dataset was derived from the reference [28]. QTN, quantitative trait nucleotide; GWAS, genome-wide association study.

To investigate the effect of the number of markers on running time, four samples with various numbers of markers (0.2, 0.5, 0.8, and 1.01 million) and a fixed sample size (500 accessions) were sampled from the real dataset from rice [28]. As a result, it took 0.23, 0.66, 1.18, and 1.61 hours, respectively (Figure 2A). This indicates the increase of running time with the increase of the number of markers. To investigate the effect of sample size on running time, 300, 600, 900, 1200, and 2262 accessions were sampled from 2262 accessions each with 1.01 million markers from the rice dataset [28]. As a result, it took 0.37, 0.78, 1.30, 2.04, and 9.56 hours, respectively (Figure 2B). This indicates that larger sample size requires much more running time than smaller ones.

To investigate the effect of the number of CPUs on speedup, one sample with 500 accessions and 1.01 million markers was analyzed by the mrMLM software under various numbers of CPUs (1 to 7). As a result, the speedups are 1.00, 1.65, 2.07, 2.45, 3.10, 3.23, and 3.52, respectively (Figure 2C; Table S1). This indicates the effectiveness of parallel computing. The relatively small speedups with 5–7 CPUs for pLARmEB and ISIS EM-BLASSO may be due to the fact that their potentially associated markers were determined at the chromosome and genome levels, respectively (Table S1). To compare the running time of various methods, one sample with 500 accessions and 1.01 million markers was analyzed by seven methods (mrMLM, FASTmrMLM, FASTmrEMMA, pLARmEB, pKWmEB, ISIS EM-BLASSO, and FarmCPU). As a result, it took 1.43, 1.08, 1.45, 1.53, 3.07, 1.06, and 1.09 hours, respectively (Figure 2D). This indicates that ISIS EM-BLASSO is the fastest one, and FASTmrMLM is equivalent with FarmCPU and faster than mrMLM.

The first to fourth experiments were conducted on the first to fourth servers, respectively (File S5).

### Real data analyses in rice, maize, and Simmental beef cattle

We re-analyzed the aforementioned three datasets in rice [28], maize [29], and Simmental beef cattle [30]. The details can be found in File S5.

**Figure 3:**
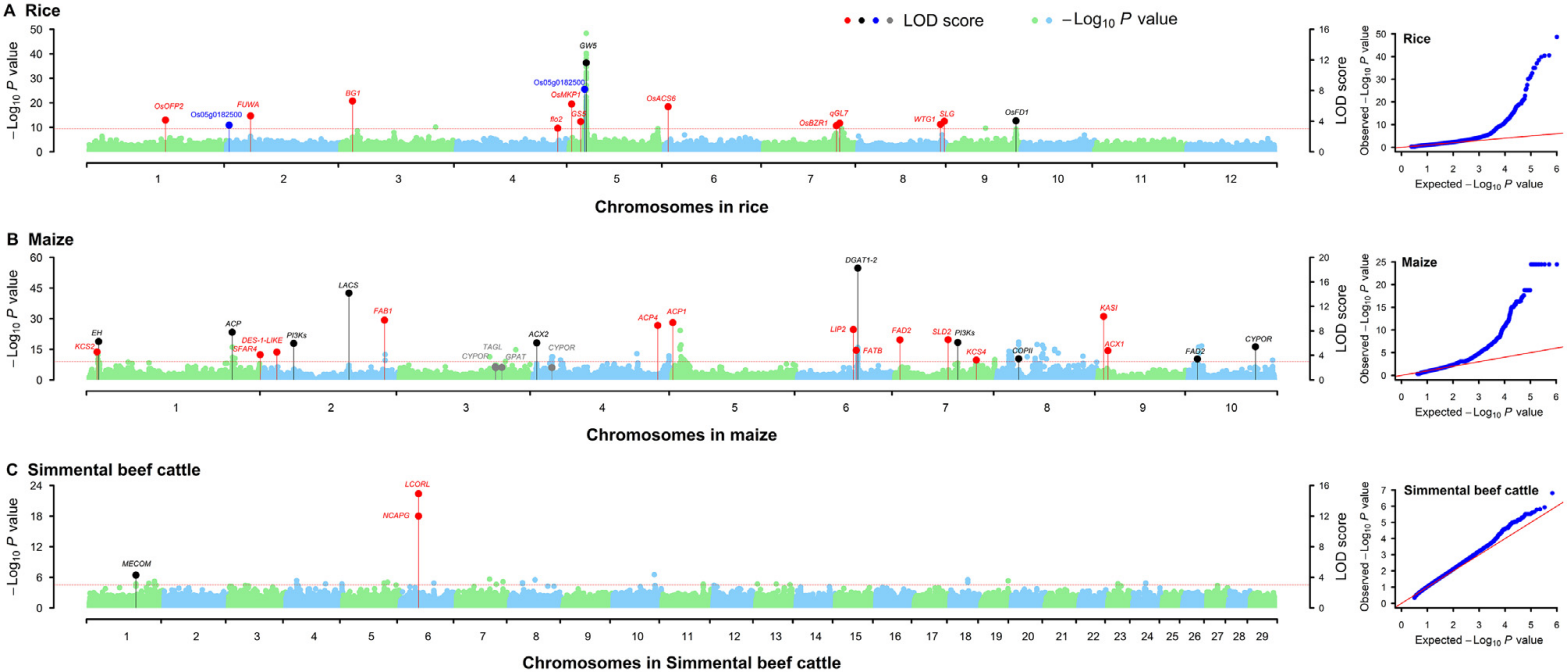
Manhattan and QQ plots for grain width, oil concentration, and kidney weight in GWAS using mrMLM v4.0.2 Left is Manhattan plot, while right is QQ plot. **A.** Grain width in rice [28]. **B.** Oil concentration in maize [29]. **C.** Kidney weight in Simmental beef cattle [30]. The dots were used to indicate the known genes detected both by mrMLM and in original studies (black), only by the software mrMLM (red), and only in original studies (grey), as well as candidate genes around QTNs from the software mrMLM (blue). QQ, quantile–quantile.

The total running time of mrMLM, FASTmrMLM, FASTmrEMMA, pLARmEB, pKWmEB, and ISIS EM-BLASSO for the rice dataset is 9.56, 3.37, 11.58, 5.09, 6.13, and 1.06 hours, respectively. Clearly, ISIS EM-BLASSO is the fastest followed by FASTmrMLM, pLARmEB, pKWmEB, and mrMLM, while FASTmrEMMA is the slowest. The total numbers of QTNs identified by the aforementioned six methods for grain width in rice are 73, 77, 42, 59, 17, and 31, respectively (Table S2). Around these QTNs, some genes have been reported to be associated with grain width. Among these reported genes, two were identified both by mrMLM and by Wang and his colleagues [28], and eleven were detected only by mrMLM (Figure 3A; Table S3). In addition, two genes were predicted to be associated with grain width in this study (Figure 3A; Table S4).

The total numbers of QTNs detected by the aforementioned six methods for oil concentration in maize are 42, 43, 31, 29, 17, and 6, respectively (Table S5). Around these QTNs, some genes have been reported to be associated with maize oil concentration. Among these reported genes, ten were identified both by mrMLM and by Li and his colleagues [29], thirteen were detected only by mrMLM, and four were identified only by Li and his colleagues [29] (Figure 3B; Table S6).

The total numbers of QTNs identified by the aforementioned six methods for kidney weight in Simmental beef cattle are 4, 55, 167, 117, 8, and 48, respectively (Table S7). Around these QTNs, some genes have been reported to be associated with kidney weight. Among these reported genes, *MECOM* was identified both by mrMLM and by An and his colleagues [31]. *LCORL* and *NCAPG*, which are very important genes for kidney weight in cattle, were detected only by mrMLM (Figure 3C; Table S8).

## Discussion

To confirm the correctness of our software mrMLM v4.0.2, the same simulation datasets (https://doi.org/10.5061/dryad.sk652) from Zhang et al. [20] (File S6) were re-analyzed by the aforementioned six methods and three current methods (GEMMA [5], FarmCPU [14], and EMMAX [4]). As a result, our six methods are better than the three current methods (Figures S2–S4; Tables S9–S11). The conclusion was also confirmed by the studies of Zhang and his colleagues [32]. As compared with the original packages of our multi-locus GWAS methods, there have been some improvements in the new version. First, the FASTmrMLM algorithm is described for the first time in this study (File S1). Then, the new package is faster in reading datasets and efficient in parallel computing (Figure 2C). Even if the sample size is larger than 2000, FASTmrEMMA is fast as well. This is because it is unnecessary to solve eigenvector at genome scan. Finally, the option for continuous covariates has been set up in order to analyze animal and human GWAS datasets. The new package works well for continuous variables in plant, animal, and human GWAS, although the current version doesn’t work for the case-control datasets in human genetics. In addition, we correct one mistake in the determination of the potentially associated SNPs in the Monte Carlo simulation studies of Zhang and his colleagues [20].

In the work of Zhang’s group [32], several major concerns in GWAS have been discussed, *i.e.*, methodological selection, the critical probability value or log of odds (LOD) score, reliable candidate genes, and heritability missing.

Using mrMLM v4.0.2, individual parameters may be changed in order to obtain the best results (Files S3 and S4). For example, the number of potentially associated SNPs for each chromosome in pLARmEB [20] is set at 50, and the search radius in mrMLM [16] and FASTmrMLM [17] is set at 20 kb in real data analysis. In addition, users should understand some parameter settings. For example, the maximum number of CPUs in parallel computation is set at 10. If users want to use more CPU cores, this parameter needs to be modified in the codes. Of course, the accuracy, size, and color of the GWAS figures and the critical LOD score line of significant QTNs may be changed as well.

## Conclusion

To popularize our multi-locus GWAS methods, six multi-locus methods have been integrated into the software mrMLM v4.0.2. In this package, three genotypic data formats are available, big dataset can be analyzed at server, parallel computation with multiple CPUs can be performed, and parameters in the GWAS figures may be set. In addition, the graphical user interface software, mrMLM.GUI v4.0.2, built upon Shiny, is available as well. Real data analyses and Monte Carlo simulation studies confirmed the advantages of our multi-locus GWAS methods.

## Code availability

mrMLM v4.0.2 and mrMLM.GUI v4.0.2 are freely available for public use at BioCode (https://bigd.big.ac.cn/biocode/tools/7077) and R (https://cran.r-project.org/web/packages/).

## CRediT author statement

**Ya-Wen Zhang:** Software, Writing - original draft. **Cox Lwaka Tamba:** Methodology. **Yang-Jun Wen:** Methodology. **Pei Li:** Software. **Wen-Long Ren:** Software. **Yuan-Li Ni:** Software. **Jun Gao:** Software. **Yuan-Ming Zhang:** Conceptualization, Supervision, Methodology, Writing - review & editing. All authors read and approved the final manuscript.

## Competing interests

The authors have declared no competing interests.

## Supplementary material

Supplementary File S1 The FASTmrMLM algorithm

Supplementary File S2 The GWAS methodologies and software packages

Supplementary File S3 User manual for mrMLM v4.0.2

Supplementary File S4 User manual for mrMLM.GUI v4.0.2

Supplementary File S5 Real data analyses in rice, maize, and Simmental beef cattle

Supplementary File S6 Monte Carlo simulation experiments

Supplementary Figure S1 Methodological comparison in mrMLM v4.0.2

Supplementary Figure S2 Comparison of powers in QTN detection between the new and existing methods in three simulation experiments A. The first simulation experiment. **B.** The second simulation experiment. **C.** The third simulation experiment. The new methods include our multi-locus GWAS methods, while the existing methods include GEMMA, EMMAX, and FarmCPU. QTN, quantitative trait nucleotide.

Supplementary Figure S3 Comparison of mean squared errors in QTN effect estimation between the new and existing methods in three simulation experiments A. The first simulation experiment. **B.** The second simulation experiment. **C.** The third simulation experiment. The new methods include our multi-locus GWAS methods, while the existing methods include GEMMA, EMMAX, and FarmCPU. QTN, quantitative trait nucleotide.

Supplementary Figure S4 Comparison of false positive rates (%) in QTN detection between the new and existing methods in three simulation experiments A. The first simulation experiment. **B.** The second simulation experiment. **C.** The third simulation experiment. The new methods include our multi-locus GWAS methods, while the existing methods include GEMMA, EMMAX, and FarmCPU. QTN, quantitative trait nucleotide.

Supplementary Table S1 The speedup in parallel computing under various numbers of CPUs and various GWAS approaches

Supplementary Table S2 All the QTNs for grain width in rice detected by our multi-locus GWAS methods

Supplementary Table S3 Previously reported genes for grain width in rice around the QTNs identified by our multi-locus GWAS methods

Supplementary Table S4 Fifteen new candidate genes for rice grain size and development detected by our multi-locus GWAS methods

Supplementary Table S5 All the QTNs for oil concentration in maize detected by our multi-locus GWAS methods

Supplementary Table S6 Comparison of seed oil related genes in maize identified by the software mrMLM in this study with those in the ref [29].

Supplementary Table S7 All the QTNs for kidney weight in Simmental beef cattle detected by our multi-locus GWAS methods

Supplementary Table S8 Previously reported genes for kidney weight in Simmental beef cattle around the QTNs identified by our multi-locus GWAS methods

Supplementary Table S9 Comparison of power (%), mean squared error (MSE), and false positive rate (FPR, %) for nine GWAS methods in the first simulation experiment

Supplementary Table S10 Comparison of power (%), MSE, and FPR (%) for nine GWAS methods in the second simulation experiment

Supplementary Table S11 Comparison of power (%), MSE, and FPR (%) for nine GWAS methods in the third simulation experiment
