# Supplementary material for: mrMLM v4.0.2: An R Platform for Multi-locus Genome-wide Association Studies
Source: Genomics Proteomics Bioinformatics. 2020 Dec 18;18(4):481–7. doi: 10.1016/j.gpb.2020.06.006 (PMC8242264; doi:10.1016/j.gpb.2020.06.006)
Supplement: Supplementary File S1 — The FASTmrMLM algorithm [file mmc1.docx]

**File S1 The FASTmrMLM algorithm**

**Genetic model**

We consider a mixed linear regression model,

(1)

where $\mathbf{y}$ is an $n\times1$ phenotypic vector of quantitative trait for $n$ individuals, $\mathbf{X}$ is an $n\times q$ incident matrix of fixed effects $\mathbf{\alpha}$ including the overall mean, $\mathbf{Z}_{\mathbf{i}}$ is an $n\times1$ vector of the *i*th SNP, $\beta_{i}$ is a random effect of the *i*th marker, it is assumed to be a normal distribution with zero mean and each marker prior variance $\sigma_{i}^{2}$, is the polygenic effect with a multivariate normal distribution with zero mean and variance $\sigma_{g}^{2}$ described by a kinship matrix $\mathbf{K}$, and $\varepsilon\text{\textasciitilde MVN}\left(0\text{,}\mathbf{I}\sigma_{e}^{2}\right)$ is residual error where $\mathbf{I}$ is an $n\times n$ identity matrix and $\sigma_{e}^{2}$ is residual variance. In this study, the kinship matrix $\mathbf{K}$ is marker inferred kinship matrix defined as $\mathbf{K}=\sum_{i=1}^{m} Z_{i}Z'_{i}/m$.

From equation (1) we have that

$\begin{matrix} \text{E}(\mathbf{y})=\mathbf{X\alpha} \\ \text{Var}(\mathbf{y})=\mathbf{Z}_{i}\mathbf{Z}'_{i}\sigma_{i}^{2}+\mathbf{K}\sigma_{g}^{2}+\mathbf{I}\sigma_{e}^{2}=\sigma_{e}^{2}\left(\mathbf{Z}_{i}\mathbf{Z}'_{i}\omega_{i}+\mathbf{K}\omega_{g}+\mathbf{I}\right) =\sigma_{e}^{2}\mathbf{H} \end{matrix}$ (2)

where $\omega_{i}=\sigma_{i}^{2}/\sigma_{e}^{2}$ and $\omega_{g}=\sigma_{g}^{2}/\sigma_{e}^{2}$ are the variance ratios and $\mathbf{H}=\mathbf{Z}_{i}\mathbf{Z}'_{i}\omega_{i}+\mathbf{K}\omega_{g}+\mathbf{I}$. In this model, we have three random effects: the *i*th marker effect, polygenic effect, and residual error. Under the pure polygenic model, we have that $\text{E}(\mathbf{y})=\mathbf{X}\alpha$ and $\text{Var}(\mathbf{y}) =\sigma_{e}^{2}\left(\mathbf{K}\omega_{g}+\mathbf{I}\right)$. We pre-estimate the value of $\omega_{g}=\sigma_{g}^{2}/\sigma_{e}^{2}$ under the pure polygenic model and fix it when testing each SNP effect in the genome-wide scanning. Using spectral decomposition, we can find a diagonal matrix $\mathbf{D}=\text{diag}\left(\delta_{1},.\cdots,\delta_{m}\right)$ and a matrix $\mathbf{U}$ such that $\mathbf{K}=\mathbf{UD}\mathbf{U}'$. Notice that spectral decomposition can be performed on $\mathbf{K}$ since as defined it is a square, symmetric matrix. Transforming $\mathbf{y}$ in Equation (1) by multiplying by $\mathbf{U}'$, we have,

(3)

Let $\mathbf{y}^{*}=\mathbf{U}'\mathbf{y}, \mathbf{X}^{*}=\mathbf{U}'\mathbf{X}, \mathbf{Z}_{i}^{\mathbf{*}}= \mathbf{U}'\mathbf{Z}_{i} ,$ then Equation (3) becomes

It follows that,

(4)

For simplicity, let $\mathbf{D}_{1}=\text{diag}\left(\delta_{1}\hat{\omega}_{g}+1,\delta_{2}\hat{\omega}_{g}+1,\cdots,\delta_{m}\hat{\omega}_{g}+1\right)$. $\hat{\omega}_{g}$ is fixed, and $\mathbf{D}_{1}$, as defined, is a positive semi-definite matrix. Therefore, we can obtain $\mathbf{D}_{\mathbf{1}}^{\mathbf{1}/\mathbf{2}}$. Further transforming $\mathbf{y}^{*}$ by multiplying equation (3) through by $\mathbf{D}_{\mathbf{1}}^{\mathbf{-}\mathbf{1}/\mathbf{2}}$ and letting $\mathbf{y}_{c}=\mathbf{D}_{\mathbf{1}}^{\mathbf{-}\mathbf{1}/\mathbf{2}}\mathbf{y}^{*},\mathbf{X}_{c}=\mathbf{D}_{\mathbf{1}}^{\mathbf{-}\mathbf{1}/\mathbf{2}}\mathbf{X}^{*},\mathbf{Z}_{c}=\mathbf{D}_{\mathbf{1}}^{\mathbf{-}\mathbf{1}/\mathbf{2}}\mathbf{Z}_{i}^{*}$ and . We have that,

(5)

Notice that the transformation in Equation (5) is equivalent to multiplying the original $\mathbf{y}$ by $\mathbf{U}'\mathbf{D}_{\mathbf{1}}^{-\frac{\mathbf{1}}{\mathbf{2}}}$, *i.e.,* $\mathbf{y}_{c}=\mathbf{U}'\mathbf{D}_{\mathbf{1}}^{-\mathbf{1}/\mathbf{2}}\left(\mathbf{y}\right)$. Now,

$\begin{matrix} \text{E}\left(\mathbf{y}_{c}\right)=\mathbf{X}_{c}\alpha\\ \text{Var}\left(\mathbf{y}_{c}\right)=\sigma_{e}^{2}\left(\mathbf{Z}_{ci}\mathbf{Z}_{ci}^{'}\omega_{i}+\mathbf{I}\right) =\sigma_{e}^{2}\left(\mathbf{A}+\mathbf{B}\right)=\sigma_{e}^{2}\mathbf{H} \end{matrix}$ (6)

where $\mathbf{A}=\mathbf{Z}_{ci}\mathbf{Z}_{ci}^{'}\omega_{i}$, $\mathbf{B}=\mathbf{I}$, and $\mathbf{H}=\mathbf{A}+\mathbf{B}$. Thus, the distribution of our transformed data vector $\mathbf{y}_{c}$ is normal with the mean $\mathbf{X}_{c}\mathbf{\alpha}$ and variance-covariance matrix $\sigma_{e}^{2}\mathbf{H} .$ The parameters to be estimated are $\sigma_{e}^{2}$ and $\omega_{i}$ for $i=1,2,\cdots,m$.

**Parameter estimation**

The profiled residual log likelihood (REML) of $\mathbf{y}^{*}$ after absorbing other terms in the constant term $C$ is,

(7)

where, $\mathbf{P}=\mathbf{H}^{-1}-\mathbf{H}^{-1}\mathbf{X}_{c}\left(\mathbf{X}_{c}^{'}\mathbf{H}^{-1}\mathbf{X}_{c}\right)^{-1}\mathbf{X}_{c}^{'}\mathbf{H}^{-1},$ and $q=\text{rank}\left(\mathbf{X}_{c}\right)$. We differentiate equation (7) to obtain REML estimates as shown below.

**Estimation of residual variance $\sigma_{e}^{2}$:** We have

This can be simplified to

$\hat{\sigma}_{e}^{2}=\frac{\mathbf{y}_{c}^{'}\mathbf{Py}_{c}}{n-q} =\frac{1}{n-q}\left[\left(\mathbf{y}_{c}^{'}\mathbf{H}^{-1}\mathbf{y}_{c}\right)-\left(\mathbf{y}_{c}^{'}\mathbf{H}^{-1}\mathbf{X}_{c}\right)\left(\mathbf{X}_{c}^{'}\mathbf{H}^{-1}\mathbf{X}_{c}\right)^{-1}\left(\mathbf{X}_{c}^{'}\mathbf{H}^{-1}\mathbf{y}_{c}\right)\right]$ (8)

**Estimation of the variance ratio** $\omega_{i}$**:** We have that

(9)

The second derivative of the residual log likelihood function with respect to $\omega_{i}$ is given below, and it is used to obtain the Hessian matrix.

(10)

Evaluation of REML equation and its derivatives require $\mathbf{H}^{-1}$ and $\left|\mathbf{H}\right|$. $\mathbf{H}$ is the sum of the above two matrices. The inverse and determinant of $\mathbf{B}$ can easily be computed because it is an identity matrix, and $\mathbf{A}$ is a matrix of rank 1. In mrMLM of the ref [1], $\mathbf{H}$ is also the sum of two matrices but in this new algorithm (FASTmrMLM) we have simplified the $\mathbf{H}$ further to be the sum of an identity matrix and a matrix of rank one. Therefore,

$\mathbf{H}^{-1} = \mathbf{B}^{-1}-\frac{1}{1+g}\mathbf{B}^{-1}\mathbf{AB}^{-1}=\mathbf{I}-\frac{1}{1+g}\mathbf{A}=\mathbf{I}-\frac{\omega_{i}}{1+g}\mathbf{Z}_{ci}\mathbf{Z}_{ci}^{'}$ (11)

and $\left|\mathbf{H}\right|=\left(1+g\right)\left|\mathbf{B}\right|=1+g ,$ where $g=\text{tr}(\mathbf{AB}^{-1})=\text{tr}(\mathbf{A})=\omega_{i} \text{tr}\left(\mathbf{Z}_{ci}\mathbf{Z}_{ci}^{'}\right)$ [2]. REML requires many quadratic terms in the form $\mathbf{\eta}^{'}\mathbf{H}^{-1}\mathbf{\tau}$, which can be expressed as

$\mathbf{\eta}^{'}\mathbf{H}^{-1}\mathbf{\tau}=\mathbf{\eta}^{'}\mathbf{\tau}-\frac{\omega_{i}}{1+g}\mathbf{\eta}^{'}\mathbf{Z}_{ci}\mathbf{Z}_{ci}^{'}\mathbf{\tau}$ (12)

where $\mathbf{\eta}^{'}$ and $\mathbf{\tau}$ can be any vectors or matrices with $\mathbf{H}^{-1}$ such as $\mathbf{X}_{c}^{'}\mathbf{H}^{-1}\mathbf{X}_{c}$, $\mathbf{y}_{c}^{'}\mathbf{Py}_{c}$ or $\mathbf{X}_{c}^{'}\mathbf{Py}_{c}$. Note that $\mathbf{\eta}^{'}\mathbf{\tau}=\sum_{j=1}^{n} \eta_{j}^{'}\tau_{j}$, where $\eta_{j}$ corresponds to the *j*th row (element) of the matrix (vector) $\mathbf{\eta}$ and $\tau_{j}$ corresponds to the *j*th row (element) of the matrix (vector) $\mathbf{\tau}$ for $j=1,2,\cdots\text{, }n\text{ .}$ Thus, the value in Equation (8) above can easily be estimated because with the help of Equation (12) each term in the bracket in Equation (8) is in the form $\mathbf{\eta}^{'}\mathbf{H}^{-1}\mathbf{\tau}$. For example, $\mathbf{y}_{c}\mathbf{H}^{-1}\mathbf{y}_{c}=\mathbf{y}_{c}^{'}\mathbf{y}_{c}-\frac{\omega_{i}}{1+g}\mathbf{y}_{c}^{'}\mathbf{Z}_{ci}\mathbf{Z}_{ci}^{'}\mathbf{y}_{c}$. Also, the gradient function / score function in Equation (9) and Hessian matrix in Equation (10) can also be expressed in the form $\mathbf{\eta}^{'}\mathbf{H}^{-1}\mathbf{\tau}$. With these simplifications, the computation of the variance ratio $\omega_{i}$ for the *i*th SNP is less computationally intensive via the Newton-Raphson method. We estimate the variance ratio $\omega_{i}$ for the *i*th SNP by equating the gradient functions to be zero using the Newton-Raphson technique. With our simplifications,

(13)

which is in the form $\mathbf{\eta}^{'}\mathbf{H}^{-1}\mathbf{\tau}$, and therefore its computation is so fast. We can also express the second derivative expression in Equation (10) in the form $\mathbf{\eta}^{'}\mathbf{H}^{-1}\mathbf{\tau}$:

With these simplifications, the Newton-Raphson will converge smoothly to the estimate value of variance ratio $\omega_{i}$ for the *i*th SNP. The gradient function and the Hessian matrix are in a simplified form. Therefore, the computations in each simulation run are fast. We have implemented this algorithm in R software.

**Empirical Bayes estimate of** **:** The joint distribution of $\mathbf{y}$ and is a multivariate normal distribution

$$\left(\begin{matrix} \mathbf{y} \\ \beta_{i} \end{matrix}\right)\sim MVN\left(\left[\begin{matrix} \mathbf{X}\alpha\\ 0 \end{matrix}\right], \left[\begin{matrix} \mathbf{Z}_{i}\mathbf{Z}'_{i}\omega_{i}+\mathbf{K}\omega_{g}+\mathbf{I} \mathbf{Z}_{i}\sigma_{i}^{2} \\ \mathbf{Z}_{i}^{'}\sigma_{i}^{2} \sigma_{i}^{2} \end{matrix}\right]\right)$$

The conditional distribution of given $\mathbf{y}$ is

$$\beta_{i}|\mathbf{y}\sim\text{N}\left[\mathbf{Z}_{i}^{'}\hat{\sigma}_{i}^{2}\left(\mathbf{Z}_{i}\mathbf{Z}'_{i}\hat{\sigma}_{i}^{2}+\mathbf{K}\hat{\sigma}_{g}^{2}+\mathbf{I}\hat{\sigma}_{e}^{2}\right)^{-1}\left(\mathbf{y}-\mathbf{X}\hat{\alpha}\right) , \right.\left.\hat{\sigma}_{i}^{2}- \hat{\sigma}_{i}^{2}\mathbf{Z}'_{i}\left(\mathbf{Z}_{i}\mathbf{Z}'_{i}\hat{\sigma}_{i}^{2}+\mathbf{K}\hat{\sigma}_{g}^{2}+\mathbf{I}\hat{\sigma}_{e}^{2}\right)^{-1}\mathbf{Z}_{i}\hat{\sigma}_{i}^{2}\right]$$

From a Bayesian analysis point of view, the conditional mean of given $\mathbf{y}$ is an empirical Bayes estimate of . Based on this framework, we obtain the Wald test statistic $\frac{\left(\text{E}\left(\beta_{i}|\mathbf{y}\right)\right)^{2}}{\text{Var}\left(\beta_{i}|\mathbf{y}\right)}\sim\chi_{1}^{2}$ (Chi-square test with 1 degree of freedom) and using this distribution we obtain P-value for each marker effect. We test each marker effect at 1% level of significance. We do not perform multiple test correction because we intend to include markers that pass this initial test in a multi-locus model.

**Detection of true QTNs in multi-locus model**

If the number of markers passing the 1% level of significance test is more than $n$, we invoke the LARS algorithm [3] to select the $n-1$ variables that are most likely associated with the quantitative trait of interest. LARS is a flexible method for variable selection, which is conducted in **lars** package (http://cran.r-project.org/web/packages/lars/) in R language. The $n-1$ markers are then included in a multi-locus model. Note that if the number of markers passing the initial test is less than $n$, we skip the LARS step and proceed to include all the selected markers in a multi-locus model. We compared various multi-locus methods: SCAD [4], adaptive Lasso [5] and EM-Empirical Bayes [6]. EM-Empirical Bayes has the highest statistical power and accuracy of the estimated marker effects. EM-Empirical Bayes is a random model method given as,

$\mathbf{y}=\mathbf{X\alpha}+\sum_{i=1}^{s} \mathbf{Z}_{i}\beta_{i}+\mathbf{\varepsilon}$ (14)

where $\mathbf{y}$, $\mathbf{X}$ and $\mathbf{\alpha}$ are the same as in model in Equation (1), $s$ is the number of potentially associated markers selected from the first step in FASTmrMLM, $\mathbf{Z}_{i}$ and $\beta_{i}$ are $n\times1$ incident vector and the random effect of the *i*th SNP, respectively. The polygenic variance is not included in the model because the model included all the potentially associated QTNs. We assume a normal prior for $\beta_{i}$, $\beta_{i}|\sigma_{i}^{2}\sim N\left(0,\sigma_{i}^{2}\right)$ and a scaled $\chi^{2}$ prior for $\sigma_{i}^{2}$, $f\left(\sigma_{i}^{2}|\tau,\omega\right)\propto\left(\sigma_{i}^{2}\right)^{-\frac{1}{2}\left(\tau+2\right)}\text{exp}\left(-\frac{\omega}{2\sigma_{i}^{2}}\right)$and we set $\left(\tau,\omega\right)=\left(0,0\right)$, which is Jeffrey’s prior $f\left(\sigma_{i}^{2}|\tau,\omega\right)=\sigma_{i}^{2}/2$ [6]. The procedure for parameter estimation in EM-Empirical Bayes is as follows:

1. Initial step: We set initial values as

(15)

1. E-step: QTN effect can be predicted by

$\text{E}\left(\beta_{i}\right)=\sigma_{i}^{2}\mathbf{Z}'_{i}\mathbf{V}^{-1}\left(\mathbf{y}-\mathbf{X}\alpha\right)$ (16)

where $\mathbf{V}=\sum_{i=1}^{s} \mathbf{Z}_{i}\mathbf{Z}'_{i}\sigma_{i}^{2}+\mathbf{I}\sigma_{e}^{2}$.

1. M-step: To update parameters $\sigma_{i}^{2}$, $\mathbf{\alpha}$ and $\sigma_{e}^{2}$

$\begin{matrix} \sigma_{i}^{2}=\frac{\text{E}\left(\beta'_{i}\beta_{i}\right)+\omega}{\tau+3} \\ \alpha=\left(\mathbf{X}'\mathbf{V}^{-1}\mathbf{X}\right)^{-1}\mathbf{X}'\mathbf{V}^{-1}\mathbf{y} \\ \hat{\sigma}_{e}^{2}=\frac{1}{n}\left(\mathbf{y}-\mathbf{X}\alpha\right)^{'}\left\{\left(\mathbf{y}-\mathbf{X}\alpha\right)-\sum_{i=1}^{s} \mathbf{Z}_{i}\text{E}\left(\beta_{i}\right)\right\} \end{matrix}$ (17)

where $\text{E}\left(\beta'_{i}\beta_{i}\right)=\text{E}\left(\beta'_{i}\right)\text{E}\left(\beta_{i}\right)+\text{tr}\left[\text{Var}\left(\beta_{i}\right)\right] ,$ $\text{Var}\left(\beta_{i}\right)=\mathbf{I}\sigma_{i}^{2}-\sigma_{i}^{2}\mathbf{Z}'_{i}\mathbf{V}^{-1}\mathbf{Z}_{i}\sigma_{i}^{2}$ and $\left(\tau,\omega\right)=\left(0,0\right)$.

We repeat E-step and M-step until convergence is satisfied. We select all SNPs with a score $\text{LOD}\geq3$ (log of odds) and regard them as significant. We term our algorithm as a fast multi-locus random-SNP-effect mixed linear model (FASTmrMLM).

**References**

[1] Wang SB, Feng JY, Ren WL, Huang B, Zhou L, Wen YJ, et al. Improving power and accuracy of genome-wide association studies via a multi-locus mixed linear model methodology. Sci Rep 2016;6:19444.

[2] Miller KS. On the inverse of the sum of matrices. Mathematics Magazine 1981;54:67–72.

[3] Robert AS. Discussion of "Least angle regression" by Efron et al. The Annals of Statistics 2004;32:490–4.

[4] Fan J, Li R. Variable selection via nonconcave penalized likelihood and its oracle properties. Publications of the American Statal Association 2001;96:1348–60.

[5] Hui Z. The adaptive Lasso and its oracle properties. Publications of the American Statal Association 2006;101:1418–29.

[6] Xu S. An expectation-maximization algorithm for the Lasso estimation of quantitative trait locus effects. Heredity 2010;105:483–94.
