# Supplementary material for: mrMLM v4.0.2: An R Platform for Multi-locus Genome-wide Association Studies
Source: Genomics Proteomics Bioinformatics. 2020 Dec 18;18(4):481–7. doi: 10.1016/j.gpb.2020.06.006 (PMC8242264; doi:10.1016/j.gpb.2020.06.006)
Supplement: Supplementary File S2 — The GWAS methodologies and software packages [file mmc2.docx]

**File S2 The GWAS methodologies and software packages**

**Multi-locus random-SNP-effect mixed linear model (mrMLM) and FASTmrMLM**

In the mrMLM of the ref [1], three techniques were used to reduce the running time in the first stage. First, the polygenic-to-residual variance ratio *λ* was estimated under a pure polygenic model (the null model) and then treated as a constant ($\hat{\lambda}$) in single-marker association in genome-wide scans. Then, the residual variance at the single-marker association was estimated together with fixed effects. More importantly, eigen decomposition for kinship matrix **K** was carried out so that $\mathbf{K}=\mathbf{UDU}^{T}$, where $\mathbf{D}=\text{diag}\left\{\delta_{1},\cdots,\delta_{n}\right\}$ is a diagonal matrix for the eigenvalues and **U** is an *n* × *n* matrix for the eigenvectors. Therefore, the special structure of $\text{R}_{k}$ in restricted likelihood function allows us to implement the Woodbury matrix identities for calculating $\left|\text{R}_{k}\right|$ and $\text{R}_{k}^{-1}$, so running time is significantly reduced. Using the above three techniques, all the markers on the genome are scanned and some potentially associated markers are selected. In the second stage, all the potentially associated markers are placed into one model; their effects are estimated by empirical Bayes; and all the non-zero effects are further identified by likelihood ratio test for true QTNs.

To further reduce the running time, the above model transformation is changed from $\mathbf{y}^{*}=\mathbf{U}^{T}\mathbf{y}$ in the ref [1] into $\mathbf{y}^{*}=\mathbf{R}^{-\frac{1}{2}}\mathbf{U}^{T}\mathbf{y}$ in the ref [2], where $\mathbf{R}=\mathbf{D}\hat{\lambda}+\mathbf{I}$. Using the results of [3], thus, $\left|\mathbf{H}\right|$, $\mathbf{H}^{\text{-}1}$ and the quadratic term with the form of $\mathbf{\eta}^{T}\mathbf{H}^{\text{-}1}\mathbf{\tau}$ can be quickly and easily calculated, where $\mathbf{H}\text{=}\mathbf{Z}_{ci}\mathbf{Z}_{ci}^{T}\omega_{i}+\mathbf{I}$, and $\mathbf{Z}_{ci}$ is a vector of genotype indicators for the *k*th marker. In addition, least angle regression [4] is used to select $n-1$ variables to be included in the multi-locus genetic model if the number of potentially associated SNPs at the first stage is more than the sample size *n*. This method named FASTmrMLM.

**ISIS EM-BLASSO**

The ref [5] developed an iterative modified-sure independence screening (ISIS) implemented by expectation-maximization (EM)-Bayesian LASSO (BLASSO), which is referred to as ISIS EM-BLASSO. In the first stage, the ISIS method was used to reduce the number of SNPs to a moderate size. In other words, we first reduce the number of SNPs by selecting only those that are significantly correlated with the trait at the 0.01 level of significance. At this case, slight correlations between SNPs and the trait can be captured. Then, the selected SNP effects are shrunk by SCAD in order to select relevant SNPs. The procedure is replicated twice, and all the selected SNPs are potentially associated with the trait. The second stage is the same as that in mrMLM. Note that both EM-BLASSO and empirical Bayes are derived from [6], although different names are used in our studies.

**Fast multi-locus random-SNP-effect EMMA** (**FASTmrEMMA**)

Wen et al. [7] proposed FASTmrEMMA, which is a multi-locus GWAS method. The first two techniques in mrMLM of the ref [1] are also adopted in FASTmrEMMA. However, two new techniques are implemented. First, we do pre-multiplication $\mathbf{C}=\mathbf{Q}_{1}\mathbf{\Lambda}_{r}^{-\frac{1}{2}}\mathbf{Q}_{1}^{T}$ for the standard mixed linear model equation, where $\mathbf{B}=\hat{\lambda}\mathbf{ZKZ}^{T}+\mathbf{I}_{n}=\left(\mathbf{Q}_{1}\mathbf{\Lambda}_{r}^{\frac{1}{2}}\mathbf{Q}_{1}^{T}\right)\left(\mathbf{Q}_{1}\mathbf{\Lambda}_{r}^{\frac{1}{2}}\mathbf{Q}_{1}^{T}\right)$, $r=\text{rank}\left(\mathbf{B}\right)$ and **Z** was the design matrix for polygenic effect. Its purpose is to whiten the covariance of kinship matrix K and residual noise. Then, the nonzero eigen decomposition of matrix $\mathbf{X}_{C}\mathbf{X}_{C}^{T}$ is the same as that of $\mathbf{X}_{C}^{T}\mathbf{X}_{C}$ (a positive number), where $\mathbf{X}_{C}\text{=}\mathbf{CX}$ and $\mathbf{X}$ was an vector of marker genotypes. This means that the number of nonzero eigenvalues is specified as one. As a result, all the formulae in the estimation of all the parameters can be simply and explicitly expressed, as described in EMMA. Using the above four techniques, all the markers on the genome are scanned and some potentially associated markers are obtained in the first stage. The second stage is the same as that in the mrMLM.

**pKWmEB**

Although non-parametric methods in GWAS are robust in QTN detection, the absence of a polygenic background control in single*-*marker association in genome-wide scans results in a high false positive rate. To overcome this shortcoming, the algorithm of the ref [7] is used to whiten the covariance matrix of kinship matrix K (polygenic background) and residual noise. Using the transferred model, the Kruskal-Wallis test along with least angle regression can be used to select all the potentially associated markers. All the selected markers are further evaluated by empirical Bayes and likelihood ratio test for true QTN detection. This is the pKWmEB method proposed by the ref [8].

**pLARmEB**

The first stages in all the above methods are involved in the one-dimensional genome-wide scan by testing one marker at a time. If we want to test all the markers on one chromosome at a time while the other markers are viewed as polygenic background, the model transformation of the ref [7] can be used to whiten the covariance matrix of kinship matrix K and residual noise. At the transferred model, least angle regression is used to select the *t* most potentially associated SNPs from all the markers on each chromosome (*t*=198 in the Monte Carlo simulation studies, and 50 in real data analysis). The second stage is the same as that in the mrMLM. This is the pLARmEB method of the ref [9].

All the above six methods are implemented in the R software package mrMLM. The critical P-value of significance is set as 0.0002, which is converted from a LOD score of 3.0 in the test statistics using $p=\text{Pr(}\chi_{df=1}^{2}\text{>3.0}\times\text{4.61)=0.0002}$ [1]. mrMLM v4.0.2 and mrMLM.GUI v4.0.2 are freely available online at <https://cran.r-project.org/web/packages/mrMLM/index.html> and <https://cran.r-project.org/>web/packages/mrMLM.GUI/index.html, respectively. The relationship among the above six methods was showed in Figure S1.

**Genome-wide efficient mixed model association (GEMMA)**

This is an existing single-locus genome scan method, a fixed model version of the original MLM [10], and is used as the gold standard of single-locus model method for comparison. It is implemented in the C software GEMMA (http://www.xzlab.org/software.html) [10]. The P-value threshold of significance is set as 0.05/*m*, where *m* is the number of markers.

**Efficient mixed-model association eXpedited (EMMAX)**

EMMAX [11] is an extension of EMMA [12], and both methods are existing single-locus genome scan GWAS methods. If each QTN explains only a small fraction of phenotypic variation for complex traits, this allows us to avoid the repetitive variance component estimation procedure. Although there is almost no improvement for statistical power in QTN detection, the running time is significantly reduced. The EMMAX software is available at http://genetics.cs.ucla.edu/emmax/. The P-value threshold of significance is the same as that for GEMMA.

**FarmCPU**

This is an existing multi-locus GWAS method [13] and is used as the gold standard of multi-locus model method for comparison. FarmCPU iteratively uses fixed and random effect models for GWAS. The P-value threshold of significance is the same as that for GEMMA. The method is implemented in the R software package MVP v2.0 (<https://github.com/XiaoleiLiuBio/MVP>).

**References**

1. Wang SB, Feng JY, Ren WL, Huang B, Zhou L, Wen YJ, et al. Improving power and accuracy of genome-wide association studies via a multi-locus mixed linear model methodology. Sci Rep 2016;6:19444.
2. Tamba CL, Zhang YM. A fast mrMLM algorithm for multi-locus genome-wide association studies. bioRxiv 2018; https://doi.org/10.1101/341784.
3. Miller KS. On the inverse of the sum of matrices. Math Mag 1981;54:67–72.
4. Efron B, Hastie T, Johnstone L, Tibshirani R. Least angle regression.Ann Stat 2004;32:407–51.
5. Tamba CL, Ni YL, Zhang YM. Iterative sure independence screening EM-Bayesian LASSO algorithm for multi-locus genome-wide association studies. PLoS Comput Biol 2017;13:e1005357.
6. Xu s. An expectation-maximization algorithm for the Lasso estimation of quantitative trait locus effects. Heredity 2010 ;105:483–94.
7. Wen YJ, Zhang H, Ni YL, Huang B, Zhang J, Feng JY, et al. Methodological implementation of mixed linear models in multi-locus genome-wide association studies. Brief Bioinform 2018;19:700–12.
8. Ren WL, Wen YJ, Dunwell JM, Zhang YM. pKWmEB: Integration of Kruskal-Wallis test with empirical Bayes under polygenic background control for multi-locus genome-wide association study. Heredity 2018;120:208–18.
9. Zhang J, Feng JY, Ni YL, Wen YJ, Niu Y, Tamba CL, et al. pLARmEB: integration of least angle regression with empirical Bayes for multilocus genome-wide association studies. Heredity 2017;118:517–24.
10. Zhou X, Stephens M. Genome-wide efficient mixed-model analysis for association studies. Nat Genet 2012;44:821–4.
11. Kang HM, Sul JH, Service SK, Zaitlen NA, Kong SY, Freimer NB, et al. Variance component model to account for sample structure in genome-wide association studies. Nat Genet 2010;42:348–54.
12. Kang HM, Zaitlen NA, Wade CM, Kirby A, Heckerman D, Daly MJ, et al. Efficient control of population structure in model organism association mapping. Genetics 2008;178:1709–23.
13. Liu X, Huang M, Fan B, Buckler ES, Zhang Z. Iterative usage of fixed and random effect models for powerful and efficient genome-wide association studies. PLoS Genet 2016;12:e1005767.
