# Supplementary material for: mrMLM v4.0.2: An R Platform for Multi-locus Genome-wide Association Studies
Source: Genomics Proteomics Bioinformatics. 2020 Dec 18;18(4):481–7. doi: 10.1016/j.gpb.2020.06.006 (PMC8242264; doi:10.1016/j.gpb.2020.06.006)
Supplement: Supplementary File S3 — User manual for mrMLM v4.0.2 [file mmc3.docx]

**File S3 User manual for software mrMLM v4.0.2**

**Disclaimer**

While extensive testing has been performed by Yuan-Ming Zhang’s Lab at Crop Information Center of College of Plant Science and Technology, Huazhong Agricultural University, the results are, in general, reliable, correct or appropriate. However, results are not guaranteed for any specific datasets. We strongly recommend that users validate the mrMLM results with other software packages, *i.e.*, GEMMA, EMMAX, GAPIT v2 & PLINK.

**Download website**

<https://cran.r-project.org/web/packages/mrMLM/index.html> (R3.6.3) or https://bigd.big.ac.cn/biocode/tools/BT007077 (R3.6.3).

**Citation**

| Method or software | References |
| --- | --- |
| mrMLM | Wang et al. ***Scientific Reports*** 2016, **6**:19444. |
| ISIS EM-BLASSO | Tamba et al. ***PLoS Computational Biology*** 2017, 13: e1005357. |
| pLARmEB | Zhang et al. ***Heredity*** 2017, 118: 517–524. |
| FASTmrEMMA | Wen et al. ***Briefings in Bioinformatics*** 2018, 19(4): 700–712. |
| pKWmEB | Ren et al. ***Heredity*** 2018, 120: 418–428. |
| FASTmrMLM | Tamba & Zhang, ***bioRxiv***, 2018, doi: https://doi.org/10.1101/341784. |
|  | Zhang et al. ***Genomics, Proteomics & Bioinformatics*** 2020, doi: https://doi.org/10.1016/j.gpb.2020.06.006 |
| Software mrMLM | Zhang et al. ***Genomics, Proteomics & Bioinformatics*** 2020, doi: https://doi.org/10.1016/j.gpb.2020.06.006 |

*Note*: These references are listed in section of References.

This work was supported by the National Natural Science Foundation of China (Grant Nos. 31571268, 31871242, and U1602261), Huazhong Agricultural University Scientific & Technological Self-innovation Foundation (Grant No. 2014RC020), and State Key Laboratory of Cotton Biology Open Fund (Grant Nos. CB2017B01 and CB2019B01).

**1 INTRODUCTION**

**1.1 Why mrMLM?**

**mrMLM** (**m**ulti-locus **r**andom-SNP-effect **M**ixed **L**inear **M**odel) program is an R package for multi-locus genome-wide association studies (GWAS). At present this program (v4.0) includes six methods: 1) mrMLM, 2) FASTmrEMMA (Fast multi-locus random-SNP-effect EMMA), 3) ISIS EM-BLASSO (Iterative Sure Independence Screening EM-Bayesian LASSO), 4) pLARmEB (polygenic-background-control- based least angle regression plus empirical Bayes), 5) pKWmEB (polygenic- background-control-based Kruskal-Wallis test plus empirical Bayes); and 6) fast mrMLM (FASTmrMLM).

mrMLM 4.0.2 works well on Windows, Linux (desktop) and MacOS.

**1.2** **Getting started**

The software package mrMLM runs only in the R software environment and can be freely downloaded from https://bigd.big.ac.cn/biocode/tools/7077, or requested from the maintainer, Dr Yuan-Ming Zhang at College of Plant Science and Technology, Huazhong Agri Univ ([soyzhang@mail.hzau.edu.cn](mailto:soyzhang@mail.hzau.edu.cn)).

Note: Users may need to install Rtools <https://cran.r-project.org/bin/windows/Rtools/> and add it into the system of PATH (Figure 1). The purpose is to ensure that the results can be written to the computer.


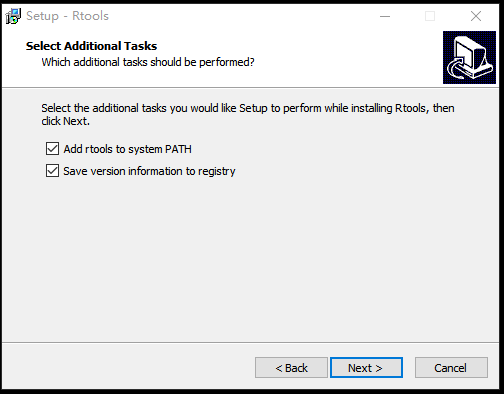


**Figure 1 Install Rtools**

**1.2.1 One-Click installation**

Within R environment, the mrMLM software can be installed online using the below command:

install.packages("mrMLM")

**1.2.2 Step-by-step installation**

**1.2.2.1 Install the add-on packages**

**Offline installation** Users may download the below 25 packages from [CRAN](https://cran.r-project.org/) (<https://cran.r-project.org/>), github (https://github.com/) and google search.

coin, colorspace, data.table, doParallel, foreach, iterators, lars, later, libcoin, lpsolve, matrixStats, modeltools, multcomp, mvtnorm, ncvreg, pillar, Rcpp, RcppArmadillo, RcppEigen, sampling, sandwich, sbl, TH.data, tibble, zoo.

Under the R environment, then, users find “Packages”—“Install package(s) from local files…”, select all the above 25 packages, and install them offline.

**1.2.2.2 Install mrMLM**

Open R GUI, select "Packages"—"Install package(s) from local files…" and then find the mrMLM package which you have downloaded on your desktop.

**User Manual file** Users can decompress the mrMLM package and find the User Manual file (name: **Instruction.pdf**) in the folder of “…/mrMLM/inst/doc”.

**1.2.3 Run mrMLM**

Once the software mrMLM is installed, users may run it using two commands:

library("mrMLM")

mrMLM(***) (***: please see § 2.1.2 Example)

If users re-use the software mrMLM, users also use the above two commands.

1. **Function**
   1. **mrMLM()**

**2.1.1 Parameter settings**

| Parameter | Meaning | File format | Note |
| --- | --- | --- | --- |
| fileGen | File path & name in your computer, *i.e.*,  fileGen="D:/Users/Genotype_num.csv" | *.csv; *.txt (Genotypic values. **Row**: markers; **Column**: individuals) | Tables 1–3 |
| filePhe | File path & name in your computer, *i.e.*,  filePhe="D:/Users/Phenotype.csv" | *.csv; *.txt (Phenotypic values. **Row**: individual; **Column**: traits) | Table 4 |
| fileKin | File path & name in your computer, *i.e.*,  fileKin="D:/Users/Kinship.csv" or fileKin=NULL | *.csv; *.txt (Kinship matrix. **Row** & **Column**: individuals) | Table 5 |
| filePS | File path & name in your computer,*i.e.*,  filePS="D:/Users/PopStr.csv" or filePS=NULL | *.csv; *.txt [Population structure. **Row**: individual; **Column**: sub-populations 1, 2, ┄, *k* (No. of sub-populations)] | Table 6–8 |
| PopStrType | Three types of population structures: *Q* (*Q* matrix), PCA (principal components), EvolPopStr (evolutionary population structure) | | |
| fileCov | File path & name in your computer,*i.e.*, fileCov="D:/Users/Covariate.csv" or fileCov=NULL | *.csv; *.txt (Covariate. **Row**: individual; **Column**: covariate 1, 2, ┄, *k* (No. of covariate)) | Table 9 |
| Genformat | Format for genotypic codes: Num (number), Cha (character) & Hmp (Hapmap), *i.e.*, Genformat="Num" | | |
| Method | Six multi-locus GWAS methods. Users may select one to six methods. For example,  method=c("mrMLM","FASTmrMLM","FASTmrEMMA","pLARmEB","pKWmEB","ISIS EM-BLASSO") | | |
| Likelihood | This parameter is only for FASTmrEMMA, including restricted maximum likelihood (REML) and maximum likelihood (ML).  Likelihood="REML" or Likelihood="ML" | | |
| Trait | Traits analyzed from number 1 to number 2. For example, **trait=1:3** indicates that users analyze the first to third traits. | | |
| SearchRadius | This parameter is only for mrMLM and FASTmrMLM, indicating Search Radius in search of potentially associated QTN.  **SearchRadius=20** indicates that only one potentially associated QTN was selected within 20 kb. | | |
| CriLOD | Critical LOD score for significant QTN. **CriLOD=3** indicates that the critical LOD score for significant QTN is set at 3.0. | | |
| SelectVariable | This parameter is only for pLARmEB. **SelectVariable=50** indicates that 50 potentially associated variables are selected from each chromosome. Users may change this number in real data analysis in order to obtain the best final results. | | |
| Bootstrap | This parameter is only for pLARmEB, including FALSE & TRUE. **Bootstrap=FALSE** indicates the analysis of only real dataset;  **Bootstrap=TRUE** indicates the analysis of both real dataset and four resampling datasets. | | |
| DrawPlot | This parameter is for all the six methods, including FALSE and TRUE. **DrawPlot=FALSE** indicates no figure output; **DrawPlot=TRUE** indicates the output of the Manhattan, QQ and LOD score against genome position figures. | | |
| Plotformat | This parameter is for all the figure files, including *.jpeg, *.png, *.tiff and *.pdf. **Plotformat="jpeg"** indicates the *.jpeg format of plot file. | | |
| dir | Save path in your computer, *i.e*, "D:/Users" | | |

**2.1.2 Example**

**The full codes**

mrMLM(fileGen="D:/Users/Genotype_num.csv",filePhe="D:/Users/Phenotype.csv",fileKin=NULL,filePS=NULL,PopStrType=NULL,fileCov=NULL,Genformat="Num",method=c("mrMLM","FASTmrMLM","FASTmrEMMA","pLARmEB","pKWmEB","ISIS EM-BLASSO"),Likelihood="REML",trait=1:3,SearchRadius=20,CriLOD=3, SelectVariable=50,Bootstrap=FALSE,DrawPlot=FALSE, Plotformat="jpeg", dir="D:/Users")

**The reduced codes**

mrMLM(fileGen="D:/Users/Genotype_num.csv", filePhe="D:/Users/Phenotype.csv", Genformat="Num", method=c("mrMLM","FASTmrMLM","FASTmrEMMA","pLARmEB","pKWmEB","ISIS EM-BLASSO"), trait=1:3, CriLOD=3, dir="D:/Users")

It should be noted that users must set "fileGen", "filePhe", "Genformat", "method", "trait", "CriLOD" and "dir", and the other eight parameters can be default in function, including PopStrType="Q"; Likelihood="REML"only for FASTmrEMMA; SearchRadius=20 only for mrMLM and FASTmrMLM; SelectVariable=50 & Bootstrap=FALSE only for pLARmEB; DrawPlot=TRUE; Plotformat= "jpeg".

**2.2 Function MultiManhattan()**

Users can use this function to draw or adjust the Manhattan plot according to their own needs.

**2.2.1 Parameter settings**

| **Parameter** | **Meaning** |
| --- | --- |
| **ResultIntermediate** | Intermediate results obtained by the mrMLM software |
| **ResultFinal** | Final results obtained by the mrMLM software |
| **mar** | A numerical vector of the form c(bottom, left, top, right), which gives the number of lines of margin to be specified on the four sides of the plot, and the default is c(2.9, 2.8, 0.7, 2.8) |
| **LabDistance** | Distance between label and axis; the default is 1.5 |
| **ScaleDistance** | Distance between scale values and axis; the default is 0.4 |
| **LabelSize** | Size of all the three labels; the default is 0.8 |
| **ScaleSize** | Size of scale values; the default is 0.7 |
| **AxisLwd** | The width of axis, a positive number; the default is 5 |
| **TckLength** | The length of tick marks; the default is -0.03 |
| **LogTimes** | Magnification of {-log10(P-value)}; the default is 2 |
| **LODTimes** | Magnification of {LOD score}; the default is 1.2 |
| **lodline** | The significant LOD score; the default is 3 |
| **dirplot** | Path to save plot; the default is current working directory |
| **PlotFormat** | Format of the plot, *i.e.*, *.tiff, *.png, *.jpeg, *.pdf |
| **width** | Figure width; the default is 28000 |
| **height** | Figure height; the default is 7000 |
| **pointsize** | Word resolution, with the unit of 1/72 inch, being pixels per inch (ppi); the default is 60 |
| **res** | Figure resolution, with the unit of pixels per inch (ppi); the default is 600 |
| **MarkGene** | To mark genes in plot or not; if “TRUE” is selected, a file, namely “Reference information to mark gene.csv”, that contains the *x* and *y* axis information of all the significant QTNs will generate. The default is “FALSE”, indicating that no candidate or known gene names are marked in Manhattan plot. |
| **Pos_x** | The *x* axis positions of all the marked genes are input |
| **Pos_y** | The *y* axis positions of all the marked genes are input |
| **GeneName** | All the gene names are input |
| **GeneNameColour** | The color of gene names |
| **…** | Arguments passed to points, axis, text |

**2.2.2 Example**

The full codes

MultiManhattan(ResultIntermediate="D:/Users/intermediate result.csv", ResultFinal="D:/Users/Final result.csv", mar=c(2.9,2.8,0.7,2.8),LabDistance=1.5,ScaleDistance=0.4,LabelSize=0.8,ScaleSize=0.7,AxisLwd=5,TckLength=-0.03,LogTimes=2,LODTimes=1.2,lodline=3,dirplot="D:/Users", PlotFormat=c("tiff","png","jpeg","pdf"), width= 28000, height=7000, pointsize = 60, res=600, MarkGene=TRUE, Pos_x=c(139,195), Pos_y=c(7.5,7), GeneName=c("Gene1","Gene2"), GeneNameColour**=**"blue")

The reduced codes

MultiManhattan(ResultIntermediate="D:/Users/intermediate result.csv", ResultFinal="D:/Users/Final result.csv")

It should be noted that users must set up two parameters "ResultIntermediate" and "ResultFinal", and the other twenty-one parameters may be default in this Function.

**2.3 Dataset format**

**Numeric format for dataset “fileGen”** (Table 1) The first column, named "**rs#**", stands for marker ID, *i.e.*, “PZB00859.1”. The second column, named "**chrom**", stands for chromosome, *i.e.*, numeric variable “1”. The third column, named "**pos**", stands for the position (bp) of SNP on the chromosome. The fourth column, named "**genotype for code 1**", indicates reference base for code variable *x* = 1. Among the remaining columns, each column lists all the genotypes for one individual, and the first row shows the individual names. For each marker, homozygous genotypes are expressed by 1 and -1, respectively, and the heterozygous and missing genotypes are indicated by zero. If the base for the first individual is missing, the base firstly observed in this row is what we list. Note that the genotype with code **1** will be also appeared in the **Result** files.

**Table 1 The numeric format of the genotypic dataset**

| rs# | chrom | pos | genotype for code 1 | 33-16 | Nov-38 | A4226 | A4722 |
| --- | --- | --- | --- | --- | --- | --- | --- |
| PZB00859.1 | 1 | 157104 | C | 1 | 1 | 1 | 1 |
| PZA01271.1 | 1 | 1947984 | C | 1 | -1 | 1 | -1 |
| PZA03613.2 | 1 | 2914066 | G | 1 | 1 | 1 | 1 |
| PZA03613.1 | 1 | 2914171 | T | 1 | 1 | 1 | 1 |
| PZA03614.2 | 1 | 2915078 | G | 1 | 1 | 1 | 1 |
| PZA03614.1 | 1 | 2915242 | T | 1 | 1 | 1 | 1 |
| PZA02117.1 | 1 | 223466480 | A | 1 | 1 | 1 | -1 |
| PZA00403.5 | 1 | 223466873 | T | 1 | 1 | 1 | 0 |
|  |  |  |  |  |  |  |  |

**Character format for dataset “fileGen”** (Table 2) The first three columns are same as those in Table 1. The differences are that the marker values are character, such as **A, T, C, G,** and **N**, and the other notations are heterozygous genotypes. The “**N**” indicates the missing of genotypes. The first rows from the fourth to last columns are individual name.

**Table 2 The character format of the genotypic** **dataset**

| rs# | chrom | pos | 33-16 | Nov-38 | A4226 | A4722 |
| --- | --- | --- | --- | --- | --- | --- |
| PZB00859.1 | 1 | 157104 | C | C | C | C |
| PZA01271.1 | 1 | 1947984 | C | G | C | G |
| PZA03613.2 | 1 | 2914066 | G | G | G | G |
| PZA03613.1 | 1 | 2914171 | T | T | T | T |
|  |  |  |  |  |  |  |

**Hapmap format for dataset “fileGen”** (Table 3) Please see the TASSEL software in details. Here we introduce simply. The first eleven columns describe the specific information of markers and individuals, and these column names must be **"rs#"**, **"alleles"**, **"chrom"**, **"pos"**, **"strand"**, **"assembly#"**, **"center"**, **"protLSID"**, **"assayLSID"**, **"panelLSID",** and **"QCcode"**. In the **"rs#"** (1st)**, "chrom"** (3rd) **and "pos"** (4th) columns, the information has been described as the above. The values of marker genotypes should be character, such as **AA, TT, CC, GG, NN, AC,** and **AG**, where the "**NN**" indicates the missing or unknown of genotypes. In the 2nd and 5th to 11th columns, **"NA"** indicates **no information** available. All the individual genotypic information will be showed from the 12th to last columns. In each column, individual name is listed in the first row, *i.e.*, “33-16”, and the others are the genotypes (character).

**Table 3 The hapmap format of the genotypic dataset**

| rs# | alleles | chrom | pos | strand | assembly# | center | protLSID | assayLSID | panelLSID | QCcode | 33-16 | … |
| --- | --- | --- | --- | --- | --- | --- | --- | --- | --- | --- | --- | --- |
| PZB00859.1 | A/C | 1 | 157104 | + | AGPv1 | Panzea | NA | NA | maize282 | NA | CC | … |
| PZA01271.1 | C/G | 1 | 1947984 | + | AGPv1 | Panzea | NA | NA | maize282 | NA | CC | … |
| PZA03613.2 | G/T | 1 | 2914066 | + | AGPv1 | Panzea | NA | NA | maize282 | NA | GG | … |
| PZA03613.1 | A/T | 1 | 2914171 | + | AGPv1 | Panzea | NA | NA | maize282 | NA | TT | … |
| PZA03614.2 | A/G | 1 | 2915078 | + | AGPv1 | Panzea | NA | NA | maize282 | NA | GG | … |
| PZA03614.1 | A/T | 1 | 2915242 | + | AGPv1 | Panzea | NA | NA | maize282 | NA | TT | … |
| PZA02117.1 | A/G | 1 | 223466480 | + | AGPv1 | Panzea | NA | NA | maize282 | NA | AA | … |
|  |  |  |  |  |  |  |  |  |  |  |  | … |

　Before implementing GWAS, the above character genotypes should be transferred into numeric information. Here the homozygous genotype of each marker for the first individual is transferred into 1, another homozygous genotype for this marker is transferred into -1, and heterozygous and missing genotypes are transferred into zero. If the base for the first individual is missing, the base firstly observed in this row is what we list.

**Format for the dataset “filePhe”** (Table 4) The **Phenotypic** file should be a file with ***.csv** or ***.txt** format. The first column lists individual ID, *i.e.*, “B46”, and “<Phenotype>” should be showed in the first row. Among the other columns, each column lists all the observations for the trait, its trait name is showed in the first row, *i.e.*, “trait1”, and phenotypic values are in the corresponding rows of their individuals. "NA" indicates the missing or unknown of phenotypes.

**Table 4 The format of Phenotypic dataset**

| <Phenotype> | trait1 | trait2 | trait3 |
| --- | --- | --- | --- |
| B46 | 42 | 43.02 | 44.32 |
| B52 | 72.5 | 71.88 | 72.8 |
| B57 | 41 | 41.7 | 41.42 |
| B64 | 74.5 | 74.43 | 74.5 |
|  |  |  |  |

**The format for dataset “fileKin”** (Table 5) The Kinship file should be a file with ***.csv** or ***.txt** format. In the first column in Table 5, “263” is sample size (*n*), and “33-16”, “Nov-38” and “A4226” are individual ID. Note that “*n*” is the number of common individuals between the phenotypic and genotypic datasets. All the kinship coefficients are listed as an *n* × *n* matrix.

fileKin=NULL indicates that the Kinship matrix is calculated by the software mrMLM. Here only the above *n* individuals are used to calculate the Kinship matrix. fileKin="D:/Users/Kinship.csv" means that the K matrix with name Kinship.csv is uploaded from the folder "D:/Users". If the number and order of individuals in Kinship.csv are not consistent with those of the above *n* individuals, our software may match the K matrix in order that the number and order of the transferred K matrix are consistent with those in the above *n* individuals.

***Q* matrix format for dataset “filePS”** (Table 6) The *Q* matrix dataset in Table 6 consists of a (*n*+2) × (*k*+1) matrix, where *n* is the number of the above common individuals and *k* is the number of sub-populations. In the first column, “<**PopStr**>” and “<**ID**>” should present in the first and second rows, respectively; “33-16”, “Nov-38”, and “A4226” are individual ID. In the 2nd to (*k*+1)-th columns, “*Q*_1_” to “*Q_k_*” indicate sub-populations. In the third row, “0.014”, “0.972”, and “0.014” are the posterior probabilities of the “33-16” individual in the 1st, 2nd, and 3rd subpopulations, respectively. When the *Q* matrix is uploaded to the software, the software will automatically delete the column whose sum is the smallest.

**Table 5 The format of the Kinship dataset**

| 263 |  |  |  |  |  |
| --- | --- | --- | --- | --- | --- |
| 33-16 | 1.00809 | 0.45954 | 0.50677 | 0.42503 | 0.45591 |
| Nov-38 | 0.45954 | 1.03352 | 0.43048 | 0.47044 | 0.39597 |
| A4226 | 0.50677 | 0.43048 | 1.01717 | 0.45409 | 0.43775 |
| A4722 | 0.42503 | 0.47044 | 0.45409 | 0.89002 | 0.34874 |
| A188 | 0.45591 | 0.39597 | 0.43775 | 0.34874 | 1.0099 |
| A214N | 0.34693 | 0.33421 | 0.39779 | 0.29244 | 0.33058 |
| A239 | 0.43593 | 0.46499 | 0.40323 | 0.36691 | 0.39597 |
| A272 | 0.34874 | 0.40505 | 0.31423 | 0.3887 | 0.44138 |
| A441-5 | 0.47952 | 0.44138 | 0.47226 | 0.47952 | 0.49224 |
| A554 | 0.39779 | 0.45954 | 0.5431 | 0.48679 | 0.4214 |
|  |  |  |  |  |  |

**Table 6 The format of the filePS dataset**

| <PopStr> |  |  |  |
| --- | --- | --- | --- |
| <ID> | Q1 | Q2 | Q3 |
| 33-16 | 0.014 | 0.972 | 0.014 |
| Nov-38 | 0.003 | 0.993 | 0.004 |
| A4226 | 0.071 | 0.917 | 0.012 |
| A4722 | 0.035 | 0.854 | 0.111 |
| A188 | 0.013 | 0.982 | 0.005 |
| A214N | 0.762 | 0.017 | 0.221 |
| A239 | 0.035 | 0.963 | 0.002 |
| A272 | 0.019 | 0.122 | 0.859 |
|  |  |  |  |

**Principal components format for dataset “filePS”** (Table 7) The principal component dataset in Table 7 consists of a (*n*+2) × (*k*+1) matrix, where *n* is the number of the common individuals and *k* is the number of principal components. In the first column, “<**PCA**>” and “<**ID**>” should present in the first and second rows, respectively; “33-16”, “Nov-38”, and “A4226” are individual ID. In the 2nd to (*k*+1)-th columns, “PC_1_” to “PC*_k_*” indicate the first to *k*-th principal components. In the second column, “0.306”, …, “0.216” are the scores of the first principal component for the 1st to 9-th individuals, respectively. Notethat the software doesn’t

delete any principle components.

**Table 7 The dataset format of principal components**

| <PCA> |  |  |  |
| --- | --- | --- | --- |
| <ID> | PC1 | PC2 | PC3 |
| 33-16 | 0.306 | 0.029 | 0.226 |
| Nov-38 | -0.708 | -2.071 | 1.413 |
| A4226 | -2.330 | 0.116 | -0.824 |
| A4722 | 1.059 | 0.470 | -1.315 |
| A188 | -2.376 | 1.087 | -0.135 |
| A214N | -2.346 | 0.516 | 0.666 |
| A239 | -0.099 | -0.318 | -0.473 |
| A272 | -0.053 | 0.093 | -0.275 |
| A441-5 | 0.216 | -0.535 | -0.159 |
|  |  |  |  |

**Evolutionary population structure format for dataset “filePS”** (Table 8) The evolutionary population structure dataset in Table 8 consists of a (*n*+2) × 2 matrix, where *n* is the number of the common individuals. In the first column, “<**EvolPopStr**>” and “<**ID**>” should present in the first and second rows, respectively; “33-16”, “Nov-38”, and “A4226” are individual ID. In the second column, “EvolType” indicates the evolutionary type, *i.e.*, the evolutionary types for individuals “33-16” and “A4722” are “A” and “B”, respectively. such as wild (A), landrace (B), and bred (C) soybeans.

**Table 8 The dataset format of evolutionary population structure**

| <EvolPopStr> |  |
| --- | --- |
| <ID> | EvolType |
| 33-16 | A |
| A4722 | B |
| A188 | A |
| A239 | B |
|  |  |

filePS=NULL indicates no inclusion of population structure in the genetic model. filePS="D:/Users/PopStr.csv" means that population structure dataset with name PopStr.csv is uploaded from the folder “D:/Users”. If the number and order of individuals in PopStr.csv aren’t consistent with those of the above common individuals, our software may match the population structure matrix in order that the number and order of new matrix are consistent with those in the above common individuals.

**The format for dataset “fileCov”** (Table 9) The “**Covariate**” dataset consists of the (*n*+2) × (*k*+1) matrix, where *n* is the number of the common individuals and *k* is the number of covariates. In the first column, “<**Covariate**>” and “<**ID**>” should present in the first and second rows, respectively. If covariate is categorical, it should be named as Cate_covariate*. If covariate is continuous, it should be named as Con_covariate* (Table 9).

**Table 9 The format of the fileCov dataset**

| <Covariate> |  |  |  |  |
| --- | --- | --- | --- | --- |
| <ID> | Cate_covariate1 | Cate_covariate2 | Con_covariate1 | Con_covariate2 |
| 33-16 | A | C | 349.5 | 374 |
| Nov-38 | B | C | 205 | 452 |
| A4226 | A | D | 300 | 374 |
| A4722 | A | D | 190 | 452 |
| A188 | B | C | 213 | 374 |
|  |  |  |  |  |

fileCov=NULL indicates no inclusion of covariates in the genetic model. fileCov="D:/Users/covariate.csv" means that the covariates with name covariate.csv are uploaded from the folder “D:/Users”. If the number and order of individuals in the uploaded file are not consistent with those in the above common individuals, our software need to change the number and order of individuals in order to match the above datasets.

**2.4 Result**

Once the running of the software mrMLM v4.0.2 is ended, the “results” files will appear on the Directory, which was set up by users before running the software. The results for each trait include “*_intermediate result.csv”, “*_Final result.csv”, and Manhattan and QQ plots.

In the *_intermediate result.csv file, there are thirteen columns, including Trait ID, Trait name, reference sequence number (rs#, marker name), chromosome, marker's position (bp) on the chromosome, SNP effect ($\gamma_{k}$, Effect) (mrMLM, FASTmrMLM, and FASTmrEMMA), -log_10_(P) (mrMLM, FASTmrMLM, FASTmrEMMA, and pKWmEB), and genotype for code 1.

In the Final result file, there are fourteen columns, including Trait ID, Trait name, method, reference sequence number (rs#, marker names), chromosome, marker's position (bp) in the chromosome, QTN effect, LOD score, -log_10_(P), the proportion of phenotypic variance explained by significant QTN (r^2^), minor allelic frequency, genotype for code 1, residual error variance, and total phenotypic variance.

In the Manhattan plot, each marker -log_10_(P) median among the -log_10_(P) values from the mrMLM, FASTmrMLM, FASTmrEMMA, and pKWmEB approaches is used to draw the Manhattan plots. If users do not select one of the above four approaches, the software program does not produce the Manhattan plot. In the Manhattan plot, these dots are indicated by light colors. All the QTNs commonly identified by multiple approaches are indicated by the pink dots that are shown above dotted vertical lines, while all the QTNs identified by one single approach are indicated by the light color dots that are also shown above dotted vertical lines (Figure 2).


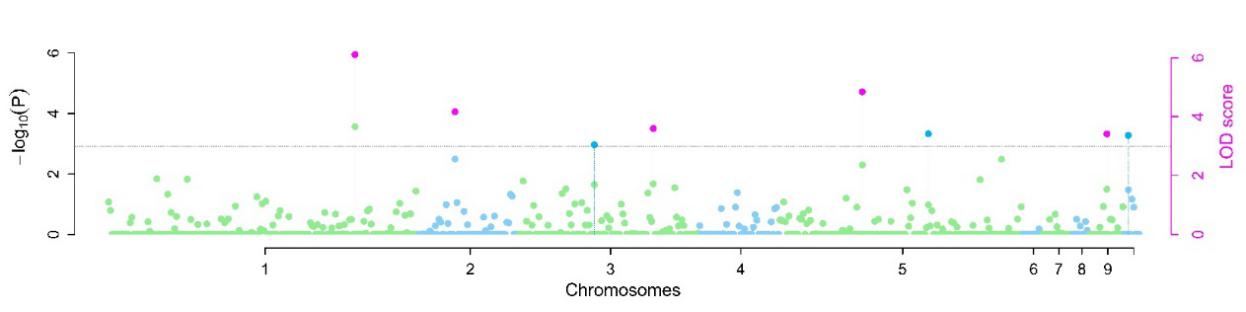


**Figure 2 Manhattan plot**

The setups for the resolution of the Manhattan plot are default. If users select the format of *.pdf, the Figure width is 16 [with the unit of inches], Figure height is 4 [with the unit of inches], and Word resolution is 20 [with the unit of 1/72 inch, ppi]. If users select the other three format, Figure width is 28,000, Figure height is 7000 [with the unit of pixel (px)], word resolution is 60 [with the unit of 1/72 inch, being pixels per inch (ppi)], and Figure resolution is 600 [with the unit of pixels per inch (ppi)].

Using the P-values in Figure 2, it is easy to draw the QQ plot (Figure 3). If users do not select one of the above four approaches, the software program does not produce the QQ plot. The setups for the resolution of the QQ plot are default. If users select the format of *.pdf, the Figure width is 7 [with the unit of inches], Figure height is 7 [with the unit of inches], and Word resolution is 25 [with the unit of 1/72 inch, ppi]. If users select the other three format, Figure width is 10,000, Figure height is 10,000 [with the unit of pixel (px)], word resolution is 60 [with the unit of 1/72 inch, being pixels per inch (ppi)], and Figure resolution is 600 [with the unit of pixels per inch (ppi)].


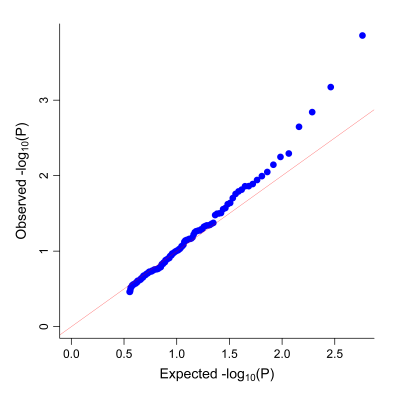


**Figure 3. QQ plot**
