# Supplementary material for: mrMLM v4.0.2: An R Platform for Multi-locus Genome-wide Association Studies
Source: Genomics Proteomics Bioinformatics. 2020 Dec 18;18(4):481–7. doi: 10.1016/j.gpb.2020.06.006 (PMC8242264; doi:10.1016/j.gpb.2020.06.006)
Supplement: Supplementary File S4 — User manual for mrMLM.GUI v4.0.2 [file mmc4.docx]

**File S4 User manual for software mrMLM.GUI v4.0.2**

**Disclaimer**

While extensive testing has been performed by Yuan-Ming Zhang’s Lab at the Crop Information Center of College of Plant Science and Technology, Huazhong Agricultural University, the results are, in general, reliable, correct or appropriate. However, results are not guaranteed for any specific datasets. We strongly recommend that users validate the mrMLM.GUI results with other software packages, *i.e.*, GEMMA, EMMAX, GAPIT v2, and PLINK.

**Download website**

<https://cran.r-project.org/web/packages/mrMLM.GUI/index.html> (R3.6.3) or https://bigd.big.ac.cn/biocode/tools/BT007077 (R3.6.3)

| Method or software | References |
| --- | --- |
| mrMLM | Wang et al. ***Scientific Reports*** 2016, 6:19444 |
| ISIS EM-BLASSO | Tamba et al. ***PLoS Computational Biology*** 2017, 13: e1005357. |
| pLARmEB | Zhang et al. ***Heredity*** 2017, 118: 517–524 |
| FASTmrEMMA | Wen et al. ***Briefings in Bioinformatics*** 2018, 19: 700–712. |
| pKWmEB | Ren et al. ***Heredity*** 2018, 120: 418–428 |
| FASTmrMLM | Tamba & Zhang, ***bioRxiv*,** 2018, doi: https://doi.org/10.1101/341784 |
|  | Zhang et al. ***Genomics, Proteomics & Bioinformatics*** 2020, doi: https://doi.org/10.1016/j.gpb.2020.06.006 |
| Software mrMLM | Zhang et al. ***Genomics, Proteomics & Bioinformatics*** 2020, doi: https://doi.org/10.1016/j.gpb.2020.06.006 |

*Note*: These references are listed in section of References.

| **m**  **r**  **M**  **L**  **M** 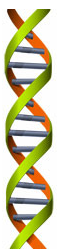 **ulti-locus**  **andom-SNP-effect**  **ixed**  **inear**  **odel** | 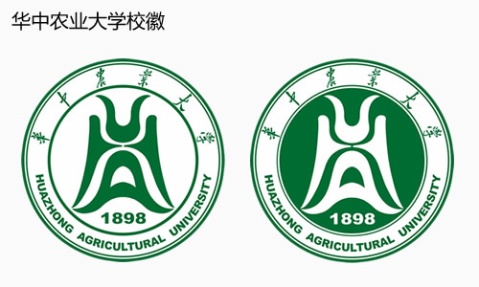 |
| --- | --- |

This work was supported by the National Natural Science Foundation of China (31571268, 31871242, and U1602261), Huazhong Agricultural University Scientific & Technological Self-innovation Foundation (Program No. 2014RC020), and State Key Laboratory of Cotton Biology Open Fund (CB2017B01 & CB2019B01).

**1 INTRODUCTION**

**1.1 Why mrMLM.GUI?**

**mrMLM.GUI** (**m**ulti-locus **r**andom-SNP-effect **M**ixed **L**inear **M**odel with **G**raphical **U**ser **I**nterface) program is an R package for multi-locus genome-wide association study (GWAS). At present this program (v4.0.2) includes six methods: 1) mrMLM, 2) FASTmrEMMA (Fast multi-locus random-SNP-effect EMMA), 3) ISIS EM-BLASSO (Iterative Sure Independence Screening EM-Bayesian LASSO), 4) pLARmEB (polygenic-background-control-based least angle regression plus empirical Bayes), 5) pKWmEB (polygenic-background-control-based Kruskal-Wallis test plus empirical Bayes), and 6) FASTmrMLM (fast mrMLM).

mrMLM.GUI 4.0.2 works well on Windows, Linux (desktop), and MacOS.

**1.2** **Getting started**

The software package mrMLM.GUI runs only in the R software environment and can be freely downloaded from https://bigd.big.ac.cn/biocode/tools/7077, or requested from the maintainer, Dr Yuan-Ming Zhang at College of Plant Science and Technology, Huazhong Agri Univ ([soyzhang@mail.hzau.edu.cn](mailto:soyzhang@mail.hzau.edu.cn)).

Note: Users may need to install Rtools <https://cran.r-project.org/bin/windows/Rtools/> and add it into the system of PATH (Fig 1). Our purpose is to ensure that the results can be written to the computer.


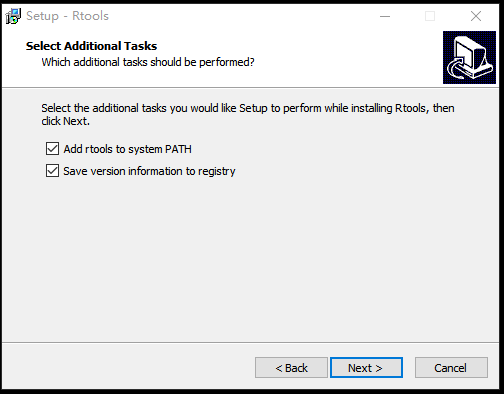


**Figure 1 Install Rtools**

**1.2.1 One-Click installation**

Within R environment, the mrMLM.GUI software can be installed online using the below command:

install.packages("mrMLM.GUI")

**1.2.2 Step-by-step installation**

**1.2.2.1 Install the add-on packages**

**Offline installation** Users may download the below 53 packages from [CRAN](https://cran.r-project.org/) (<https://cran.r-project.org/>), github (https://github.com/), and google search.

bigmemory, bigmemory.sri, calibrate, coin, colorspace, crayon, data.table, digest,

doParallel, ellipsis, fastmap, foreach, ggplot2, glue, gtable, htmltools, httpuv, iterators,

jsonlite, lars, later, libcoin, lifestyle, lpsolve, matrixStats, magrittr, mime, modeltools,

mrMLM, multcomp, munsell, mvtnorm, ncvreg, pillar, pkgconfig, promises, qqman,

R6, Rcpp, RcppArmadillo, RcppEigen, rlang, sampling, sbl, sandwich, scales, shiny,

shinyjs, TH.data, tibble, vctrs, xtable, zoo.

Under the R environment, then, users find “Packages”—“Install package(s) from local files…”, select all the above 53 packages, and install them offline.

**1.2.2.2 Install mrMLM.GUI**

Open R GUI, select "Packages"—"Install package(s) from local files…", and then find the mrMLM.GUI package in which you have downloaded on your desktop.

**User Manual** Users can decompress the mrMLM.GUI package and find the User Manual file (name: **Instruction.pdf**) in the folder of “…/mrMLM.GUI/inst/doc”.

**1.2.3 Run mrMLM.GUI**

Once the software mrMLM.GUI is installed, users may run it using two commands:

library("mrMLM.GUI")

mrMLM.GUI()

If users re-use the software mrMLM.GUI, users also use the above two commands.

1. **Dataset input**

**2.1 Genotypic dataset**

The **Genotypic** file should be a ***.csv** or ***.txt** format file.

**Numeric format for Genotypic dataset** (Table 1) The first column, named "**rs#**", stands for marker ID, *i.e.*, “PZB00859.1”. The second column, named "**chrom**", stands for chromosome, *i.e.*, numeric variable “1”. The third column, named "**pos**", stands for the position (bp) of SNP on the chromosome. The fourth column, named "**genotype for code 1**", indicates reference base for code variable *x* = 1. Among the remaining columns, each column lists all the genotypes for one individual, and the first row shows the individual names. For each marker, homozygous genotypes are expressed by 1 and -1, respectively, and the heterozygous and missing genotypes are indicated by zero. If the base for the first individual is missing, the base firstly observed in this row is what we list. Note that the genotype with code **1** will be also listed in the **Result** files.

**Table 1 The numeric format of the genotypic dataset**

| rs# | chrom | pos | genotype for code 1 | 33-16 | Nov-38 | A4226 | A4722 |
| --- | --- | --- | --- | --- | --- | --- | --- |
| PZB00859.1 | 1 | 157104 | C | 1 | 1 | 1 | 1 |
| PZA01271.1 | 1 | 1947984 | C | 1 | -1 | 1 | -1 |
| PZA03613.2 | 1 | 2914066 | G | 1 | 1 | 1 | 1 |
| PZA03613.1 | 1 | 2914171 | T | 1 | 1 | 1 | 1 |
| PZA03614.2 | 1 | 2915078 | G | 1 | 1 | 1 | 1 |
| PZA03614.1 | 1 | 2915242 | T | 1 | 1 | 1 | 1 |
| PZA02117.1 | 1 | 223466480 | A | 1 | 1 | 1 | -1 |
| PZA00403.5 | 1 | 223466873 | T | 1 | 1 | 1 | 0 |
|  |  |  |  |  |  |  |  |

**Character format for Genotypic dataset** The first three columns in Table 2 are same as those in Table 1. The differences are that the marker values are characters, such as **A, T, C, G,** and **N**, and the other notations are heterozygous genotypes. The “**N**” indicates the missing of genotypes. The first row from the fourth to last columns lists the names of individuals, *i.e.*, “33-16” and “Nov-38”.

**Table 2 The character format of the genotypic** **dataset**

| rs# | chrom | pos | 33-16 | Nov-38 | A4226 | A4722 |
| --- | --- | --- | --- | --- | --- | --- |
| PZB00859.1 | 1 | 157104 | C | C | C | C |
| PZA01271.1 | 1 | 1947984 | C | G | C | G |
| PZA03613.2 | 1 | 2914066 | G | G | G | G |
| PZA03613.1 | 1 | 2914171 | T | T | T | T |
|  |  |  |  |  |  |  |

**Hapmap format for Genotypic dataset** Please see the TASSEL software in details. Here we describe simply. The first eleven columns describe the specific information of markers and individuals, and their column names must be **"rs#"**, **"alleles"**, **"chrom"**, **"pos"**, **"strand"**, **"assembly#"**, **"center"**, **"protLSID"**, **"assayLSID"**, **"panelLSID"**, and **"QCcode"**. In the **"rs#"** (1st)**, "chrom"** (3rd), **and "pos"** (4th) columns, their information is described as the above in Table 2. The values of marker genotypes should be character, such as **AA, TT, CC, GG, NN, AC,** and **AG**, where the "**NN**" indicates the missing or unknown of genotypes. In the 2nd and 5th to 11th columns, **"NA"** indicates **no information** available. All the individual genotypic information will be showed from the 12th to last columns. In each column, individual name is listed in the first row, *i.e.*, “33-16”, and the others are the genotypes (character).

**Table 3 The hapmap format of the genotypic dataset**

| rs# | alleles | chrom | pos | strand | assembly# | center | protLSID | assayLSID | panelLSID | QCcode | 33-16 | … |
| --- | --- | --- | --- | --- | --- | --- | --- | --- | --- | --- | --- | --- |
| PZB00859.1 | A/C | 1 | 157104 | + | AGPv1 | Panzea | NA | NA | maize282 | NA | CC | … |
| PZA01271.1 | C/G | 1 | 1947984 | + | AGPv1 | Panzea | NA | NA | maize282 | NA | CC | … |
| PZA03613.2 | G/T | 1 | 2914066 | + | AGPv1 | Panzea | NA | NA | maize282 | NA | GG | … |
| PZA03613.1 | A/T | 1 | 2914171 | + | AGPv1 | Panzea | NA | NA | maize282 | NA | TT | … |
| PZA03614.2 | A/G | 1 | 2915078 | + | AGPv1 | Panzea | NA | NA | maize282 | NA | GG | … |
| PZA03614.1 | A/T | 1 | 2915242 | + | AGPv1 | Panzea | NA | NA | maize282 | NA | TT | … |
| PZA02117.1 | A/G | 1 | 223466480 | + | AGPv1 | Panzea | NA | NA | maize282 | NA | AA | … |
| PZA00403.5 | C/T | 1 | 223466873 | + | AGPv1 | Panzea | NA | NA | maize282 | NA | TT | … |
|  |  |  |  |  |  |  |  |  |  |  |  | … |

Before implementing GWAS, the above character genotypes should be transferred into numeric information. Here the homozygous genotype of each marker for the first individual is transferred into 1, another homozygous genotype for this marker is transferred into -1, and the heterozygous and missing genotypes are transferred into zero. If the base for the first individual is missing, the base firstly observed in this row is what we list.

**2.2 Phenotypic dataset**

The **Phenotypic** file with the ***.csv** or ***.txt** format is showed in Table 4. The first column lists individual ID, *i.e.*, “B46”, and “<Phenotype>” should be showed in the first row. Among the other columns, each column lists all the observations for one trait, and its trait name is showed in the first row, *i.e.*, “trait1”. "NA" indicates the missing or unknown of phenotypes.

**Table 4 The format of Phenotypic dataset**

| <Phenotype> | trait1 | trait2 | trait3 |
| --- | --- | --- | --- |
| B46 | 42 | 43.02 | 44.32 |
| B52 | 72.5 | 71.88 | 72.8 |
| B57 | 41 | 41.7 | 41.42 |
| B64 | 74.5 | 74.43 | 74.5 |
|  |  |  |  |

**2.3 Kinship dataset**

The Kinship file with the ***.csv** or ***.txt** format is showed in Table 5. In the first column, “263” is sample size (*n*), and “33-16”, “Nov-38”, and “A4226” are individual ID. Note that “*n*” is the number of common individuals between the phenotypic and genotypic datasets. All the kinship coefficients are listed as an *n* × *n* matrix.

**Table 5 The format of the Kinship dataset**

| 263 |  |  |  |  |  |
| --- | --- | --- | --- | --- | --- |
| 33-16 | 1.00809 | 0.45954 | 0.50677 | 0.42503 | 0.45591 |
| Nov-38 | 0.45954 | 1.03352 | 0.43048 | 0.47044 | 0.39597 |
| A4226 | 0.50677 | 0.43048 | 1.01717 | 0.45409 | 0.43775 |
| A4722 | 0.42503 | 0.47044 | 0.45409 | 0.89002 | 0.34874 |
| A188 | 0.45591 | 0.39597 | 0.43775 | 0.34874 | 1.0099 |
| A214N | 0.34693 | 0.33421 | 0.39779 | 0.29244 | 0.33058 |
|  |  |  |  |  |  |

When users select “**Calculate kinship (K) matrix by this software**”, these coefficients between pairs of the above common individuals in the phenotypic and genotypic datasets can be calculated. When users select to input and upload “**Kinship (K)**” matrix file, the number and order of individuals in the uploaded file may be not consistent with those in the phenotypic and genotypic datasets. At this case, our software can let the number and order of individuals in the uploaded K matrix file be consistent with those in the phenotypic and genotypic datasets.

**2.4 Population Structure dataset**

**Dataset format of *Q* matrix** The *Q* matrix dataset in Table 6 consists of a (*n*+2) × (*k*+1) matrix, where *n* is the number of the common individuals and *k* is the number of sub-populations. In the first column, “<**PopStr**>” and “<**ID**>” should present in the first and second rows, respectively; “33-16”, “Nov-38”, and “A4226” are individual ID. In the 2nd to (*k*+1)-th columns, “*Q*_1_” to “*Q_k_*” indicate sub-populations. In the third row, “0.014”, “0.972”, and “0.014” are the posterior probabilities of the “33-16” individual from the first, second, and third subpopulations, respectively. When the *Q* matrix is uploaded to the software, the software will automatically delete the column whose sum is the smallest.

**Table 6 The format of the Population Structure dataset**

| <PopStr> |  |  |  |
| --- | --- | --- | --- |
| <ID> | Q1 | Q2 | Q3 |
| 33-16 | 0.014 | 0.972 | 0.014 |
| Nov-38 | 0.003 | 0.993 | 0.004 |
| A4226 | 0.071 | 0.917 | 0.012 |
| A4722 | 0.035 | 0.854 | 0.111 |
| A188 | 0.013 | 0.982 | 0.005 |
| A214N | 0.762 | 0.017 | 0.221 |
| A239 | 0.035 | 0.963 | 0.002 |
| A272 | 0.019 | 0.122 | 0.859 |
| A441-5 | 0.005 | 0.531 | 0.464 |
|  |  |  |  |

**Dataset format of principal components** The principal component dataset in Table 7 consists of a (*n*+2) × (*k*+1) matrix, where *n* is the number of the common individuals and *k* is the number of principal components. In the first column, “<**PCA**>” and “<**ID**>” should present in the first and second rows, respectively; “33-16”, “Nov-38”, and “A4226” are individual ID. In the 2nd to (*k*+1)-th columns, “PC_1_” to “PC*_k_*” indicate the first to *k*-th principal components. In the second column, “0.306”, …, “0.216” are the scores of the first principal component for the 1st to 9-th individuals, respectively.

**Table 7 The format of the Principal components dataset**

| <PCA> |  |  |  |
| --- | --- | --- | --- |
| <ID> | PC1 | PC2 | PC3 |
| 33-16 | 0.306 | 0.029 | 0.226 |
| Nov-38 | -0.708 | -2.071 | 1.413 |
| A4226 | -2.330 | 0.116 | -0.824 |
| A4722 | 1.059 | 0.470 | -1.315 |
| A188 | -2.376 | 1.087 | -0.135 |
| A214N | -2.346 | 0.516 | 0.666 |
| A239 | -0.099 | -0.318 | -0.473 |
| A272 | -0.053 | 0.093 | -0.275 |
| A441-5 | 0.216 | -0.535 | -0.159 |
|  |  |  |  |

**Table 8 The format of the Evolutionary population structure dataset**

| <EvolPopStr> |  |
| --- | --- |
| <ID> | EvolType |
| 33-16 | A |
| Nov-38 | A |
| A4226 | A |
| A4722 | B |
| A188 | A |
| A214N | A |
| A239 | B |
|  |  |

**Dataset format of evolutionary population structure** The evolutionary population structure dataset in Table 8 consists of a (*n*+2) × 2 matrix, where *n* is the number of the common individuals. In the first column, “<**EvolPopStr**>” and “<**ID**>” should present in the first and second rows, respectively; “33-16”, “Nov-38”, and “A4226” are individual ID. In the second column, “EvolType” indicates the evolutionary type, *i.e.*, the evolutionary types for individuals “33-16” and “A4722” are “A” and “B”, respectively.

“**Not included in the model**” indicates no inclusion of population structure in the genetic model. On the contrary, it should be “**Included**”. At this case, users should upload the population structure file. If the number and order of individuals in the uploaded file aren’t consistent with those in the phenotypic and genotypic datasets, our software may change the population structure matrix in order that the number and order of individuals are consistent with those in the above common individuals.

**2.5 Covariate dataset**

The “**Covariate**” dataset in Table 9 consists of the (*n*+2) × (*k*+1) matrix, where *n* is the number of the common individuals and *k* is the number of covariates. In the first column, “<**Covariate**>” and “<**ID**>” should present in the first and second rows, respectively. The 2nd to (*k*+1)-th columns are covariates. If covariate is categorical, it should be named as Cate_covariate*. If covariate is continuous, it should be named as Con_covariate*.

**Table 9 The format of the fileCov dataset**

| <Covariate> |  |  |  |  |
| --- | --- | --- | --- | --- |
| <ID> | Cate_covariate1 | Cate_covariate2 | Con_covariate1 | Con_covariate2 |
| 33-16 | A | C | 349.5 | 374 |
| Nov-38 | B | C | 205 | 452 |
| A4226 | A | D | 300 | 374 |
| A4722 | A | D | 190 | 452 |
| A188 | B | C | 213 | 374 |
|  |  |  |  |  |

“**Not included in the model**” indicates no inclusion of covariates in the genetic model. On the contrary, it should be “**Included**”. At this case, users should upload the covariate file. If the number and order of individuals in the uploaded file aren’t consistent with those in the above common individuals, our software may change the number and order of individual in order to match the original datasets.

1. **Operation process**

**3.1 The Graphical User Interface of mrMLM.GUI**


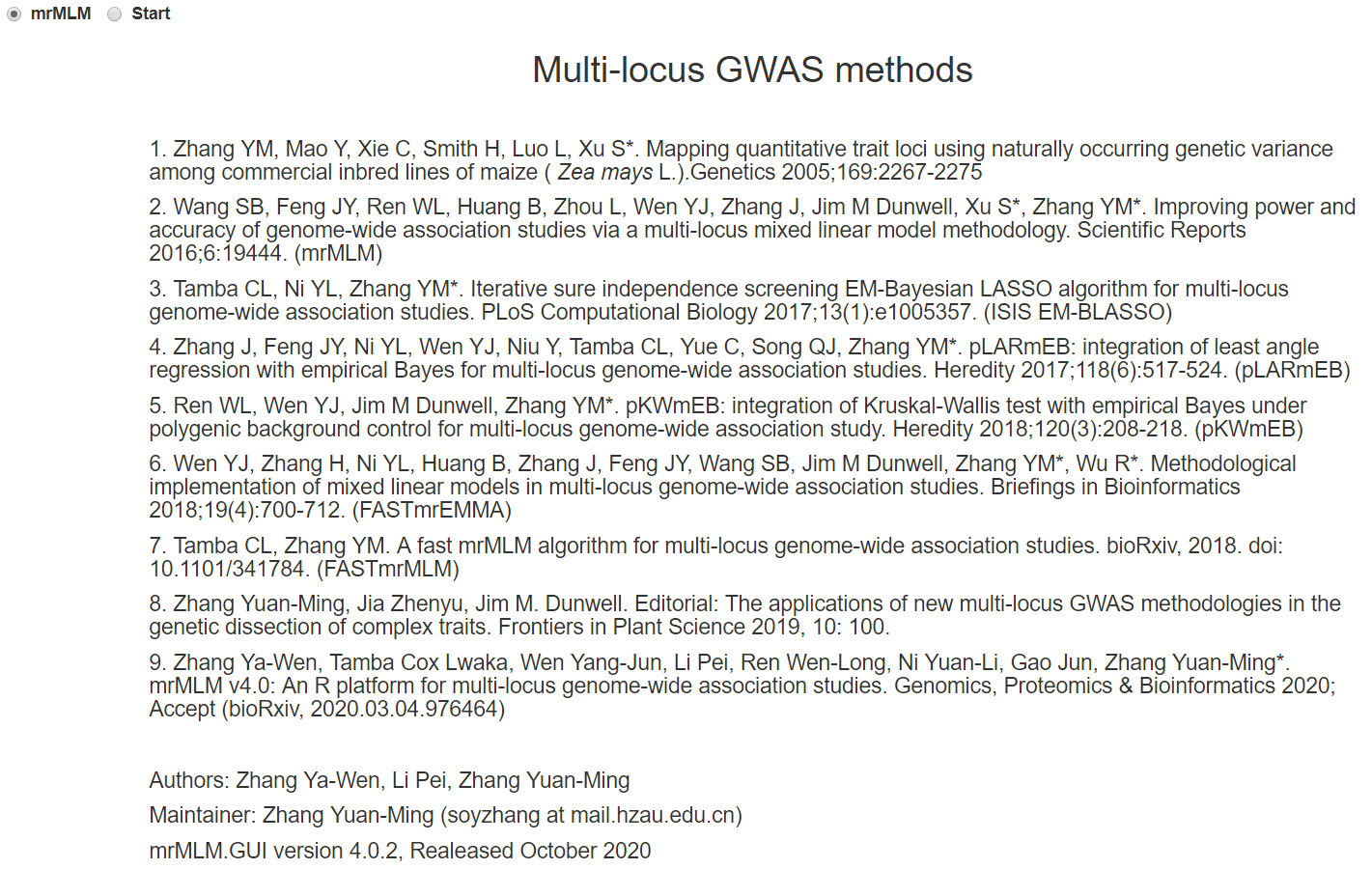


**Figure 2 The Graphical User Interface of mrMLM.GUI**

**3.2 Input dataset**

Users must upload the genotypic and phenotypic files (Figures 3 & 4), while the Kinship, Population-Structure, and Covariate files are optional. In Kinship module, users should upload the Kinship matrix if users select “**Input Kinship (K) matrix file**” (Figure 5). Users don’t need to upload this file if users select “**Calculate Kinship (K) matrix by this software**”, at this case, the K matrix can be calculated automatically. In Population Structure module, users should upload the Population Structure file if users select “**Included**” (Figure 6). There is no inclusion of population structure information in the genetic model if users select “**Not included in the model**”. In Covariate module, users should upload the covariate file if users select “**Included**” (Figure 7). There is no inclusion of covariates in the genetic model if users select “**Not included in the model**”.


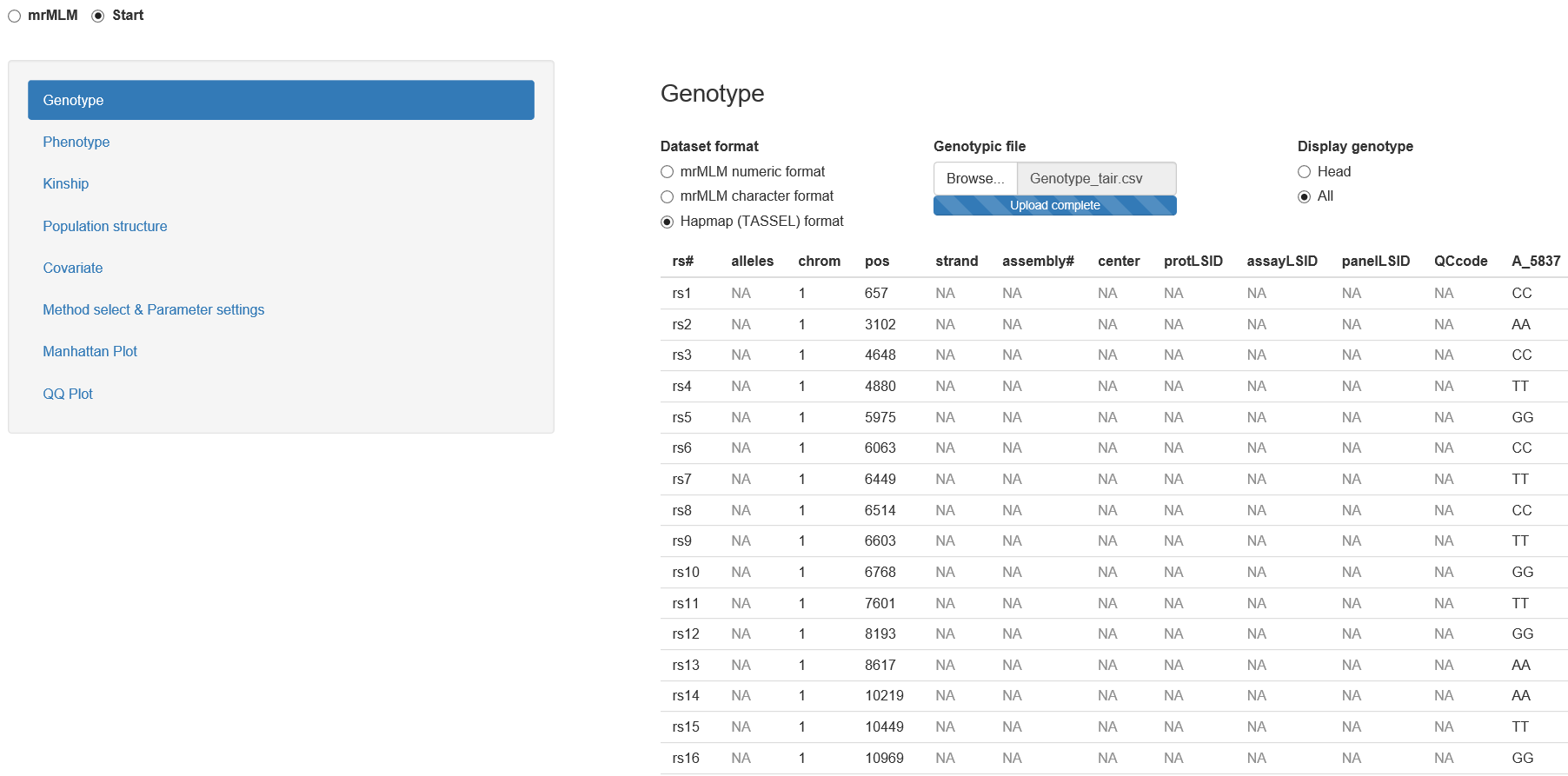


**Figure 3 Input genotypic dataset**


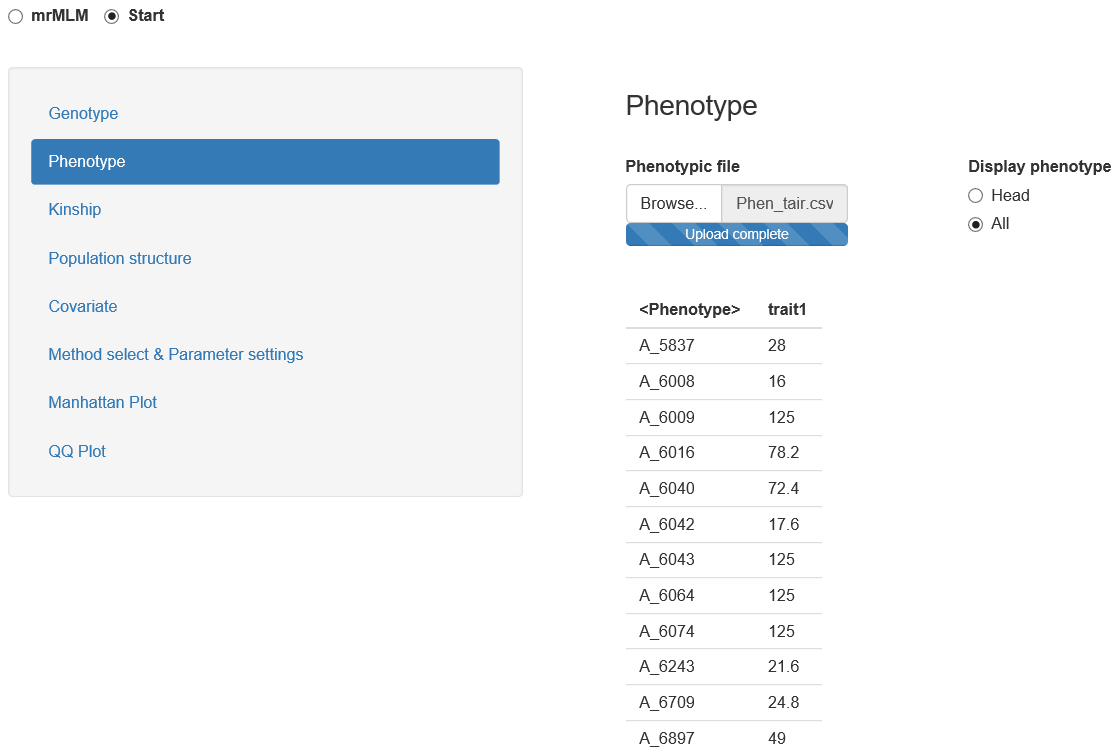


**Figure 4 Input Phenotypic dataset**


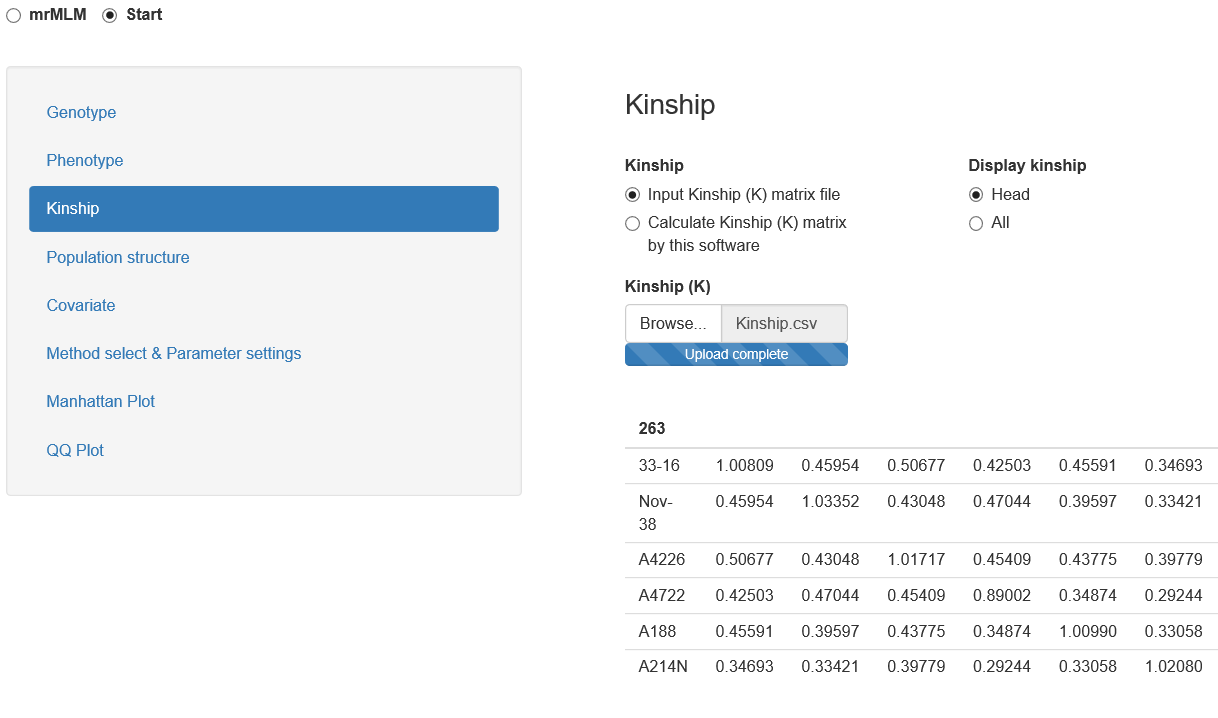


**Figure 5 Input kinship dataset**


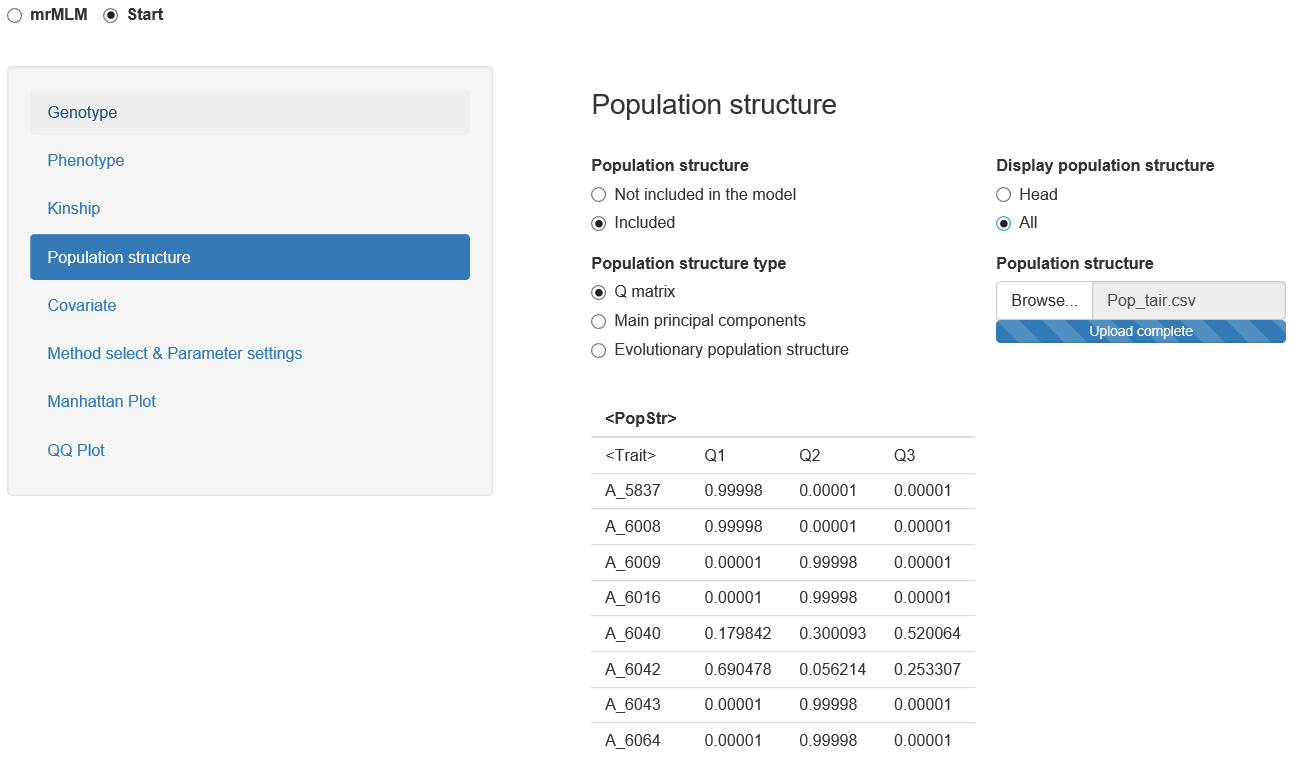


**Figure 6 Input Population Structure dataset**


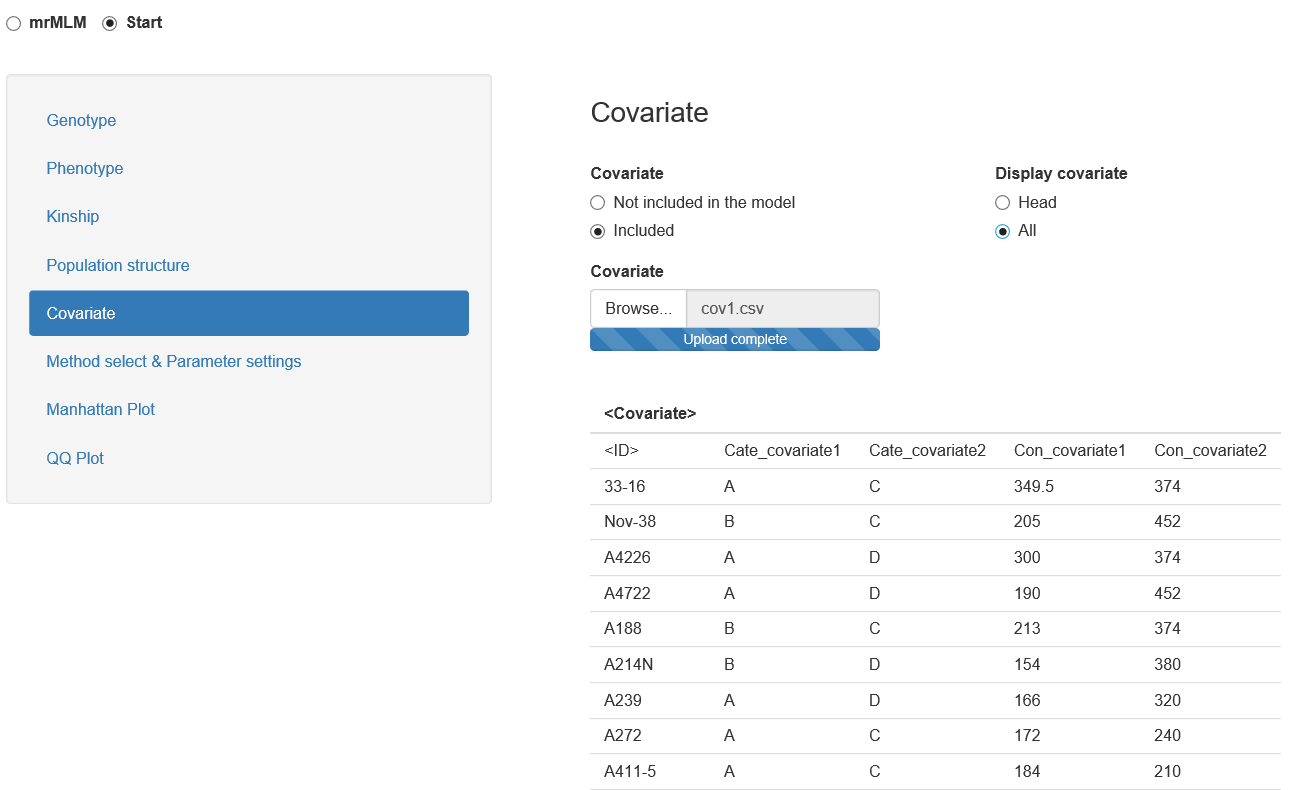


**Figure 7 Input Covariate dataset**

**3.3 Method select & Parameter setting (Figure 8)**

**Method selection:** There are six multi-locus GWAS methods available in the mrMLM.GUI. Users may select one to six methods.

**Search radius of candidate gene (kb) (mrMLM & FASTmrMLM):** This parameter is only for mrMLM and FASTmrMLM, indicating Search Radius (kb) in search of potentially associated QTN. If users set it as 20 kb, only one potentially associated QTN within the radius of 20 kb may be selected into multi-locus model.

**Likelihood function (FASTmrEMMA):** This parameter is only for FASTmrEMMA, including restricted maximum likelihood (REML) and maximum likelihood (ML).

**No. of potentially associated variables selected by LARS (pLARmEB):** This parameter is only for pLARmEB. If users set it as 50, 50 potentially associated variables can be selected from each chromosome. Users may change this number in real data analysis in order to obtain the best results.

**Bootstrap (pLARmEB):** This parameter is only for pLARmEB, including **FALSE** & **TRUE**. **FALSE** indicates only the analysis of real dataset; **TRUE** indicates the analyses of both real and four resampling datasets.

**Save path:** Save path in your computer in order to output the results in this path.

**Traits analyzed:** Traits analyzed may be from number *n*_1_ to number *n*_2_. For example, “1:3” indicates that users analyze the first to third traits.

**Draw plot (All the methods):** Including **FALSE** and **TRUE**. **FALSE** indicates no figure output; **TRUE** indicates the output of figures, including the Manhattan and QQ plots.

**Plot format (All the methods):** Including *.jpeg, *.png, *.tiff, and *.pdf for all the figure files.


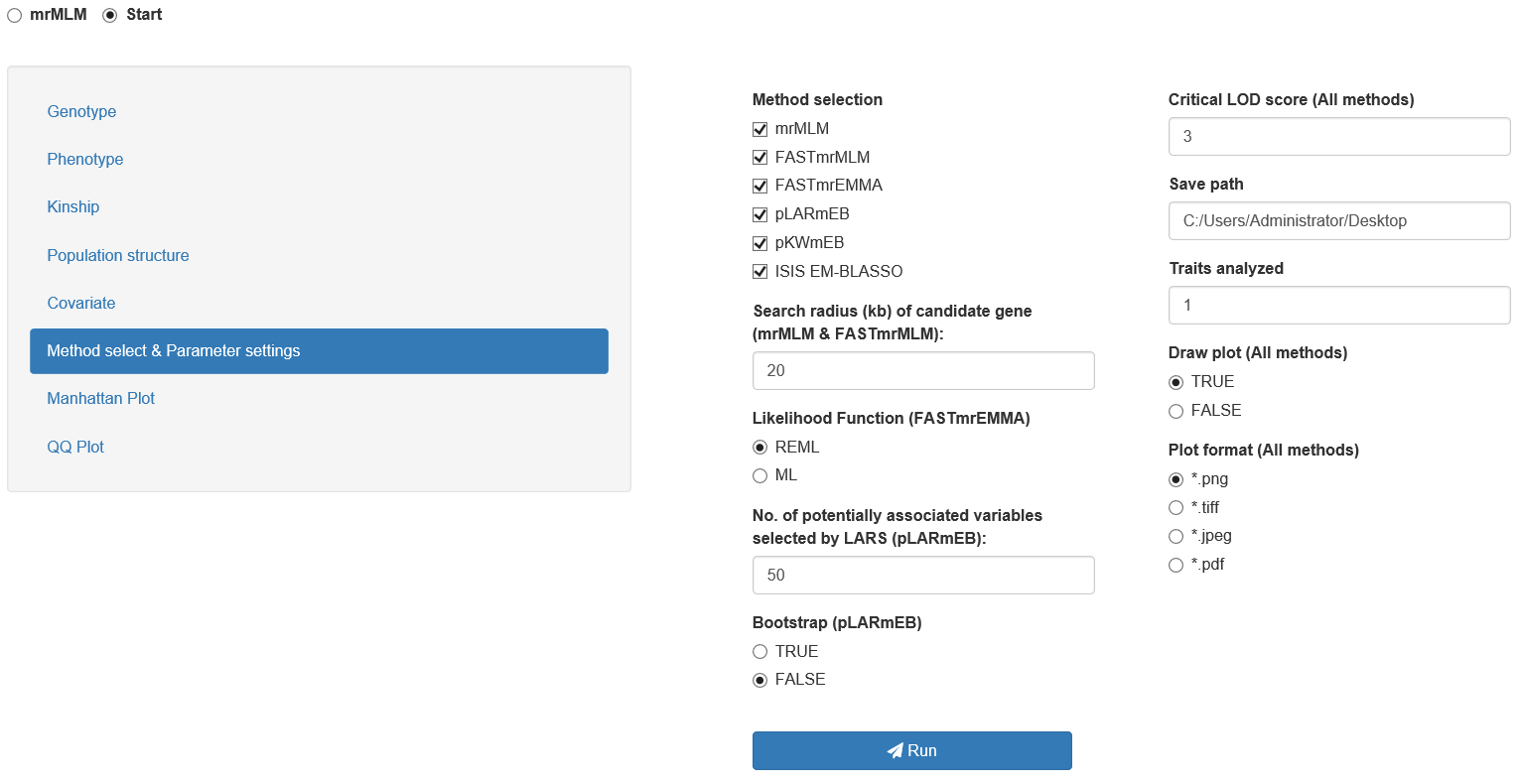


**Figure 8 Method select & Parameter setting**

**3.4 Run the software (Figure 9)**

After uploading all the needed files and setting all the parameters, users can run the software. The result files will be saved to the path that users set up.


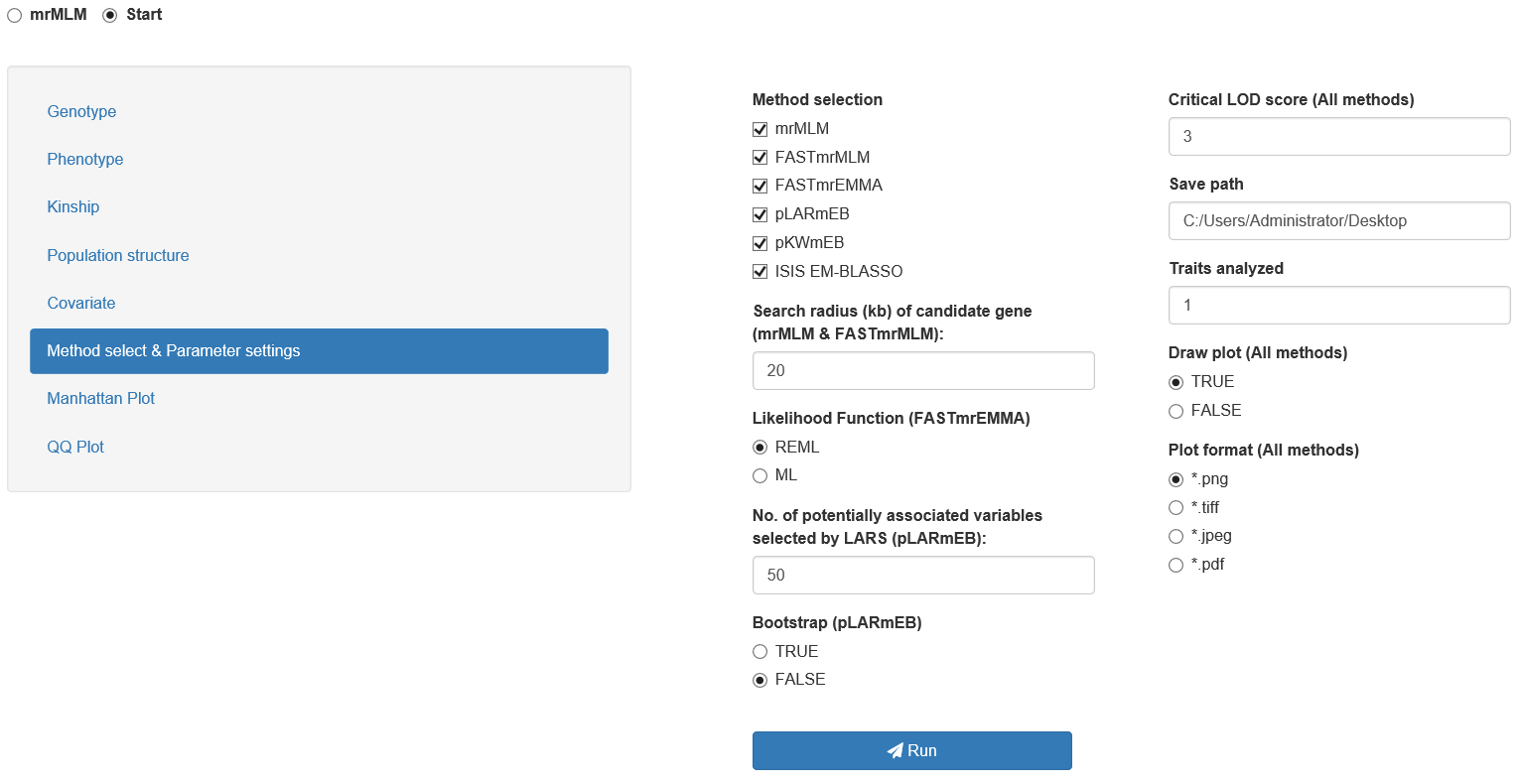


**Figure 9 Run the software mrMLM.GUI**

**4 Result**

Once the running of the software mrMLM.GUI v4.0.2 is ended, the “results” files will appear on the Directory, which was set up by users before running the software. The results for each trait include “*_intermediate result.csv”, “*_Final result.csv”, and the Manhattan and QQ plots.

In the *_intermediate result.csv file, there are thirteen columns, including Trait ID, Trait name, reference sequence number (rs#, marker name), chromosome, marker's position (bp) on the chromosome, SNP effect ($\gamma_{k}$, Effect) (mrMLM, FASTmrMLM, and FASTmrEMMA), -log_10_(P) (mrMLM, FASTmrMLM, FASTmrEMMA, and pKWmEB), and genotype for code 1.

In the Final result file, there are fourteen columns, including Trait ID, Trait name, method, reference sequence number (rs#, marker names), chromosome, marker's position (bp) in the chromosome, QTN effect, LOD score, -log_10_(P), the proportion of phenotypic variance explained by significant QTN (r^2^), minor allelic frequency, genotype for code 1, residual error variance, and total phenotypic variance.

In the Manhattan plot, each marker -log_10_(P) median among the -log_10_(P) values from the mrMLM, FASTmrMLM, FASTmrEMMA, and pKWmEB approaches is used to draw the Manhattan plot. If users do not select one of the above four approaches, the software program does not produce the Manhattan plot. All the dots in Manhattan plot are indicated by light colors. All the QTNs commonly identified by multiple approaches are indicated by the pink dots that are shown above dotted vertical lines, while all the QTNs identified by one single approach are indicated by the light color dots that are shown above dotted vertical lines (Figure 10). This plot is high-resolution. If the users want to change the plot resolution, please see the fifth section (Re-draw the plot according to user’s requirement).

The setups for the resolution of the Manhattan plot are default. If users select the format of *.pdf, the Figure width is 16 [with the unit of inches], Figure height is 4 [with the unit of inches], and Word resolution is 20 [with the unit of 1/72 inch, ppi]. If users select the other three format, Figure width is 28,000, Figure height is 7000 [with the unit of pixel (px)], Word resolution is 60 [with the unit of 1/72 inch, being pixels per inch (ppi)], and Figure resolution is 600 [with the unit of pixels per inch (ppi)].


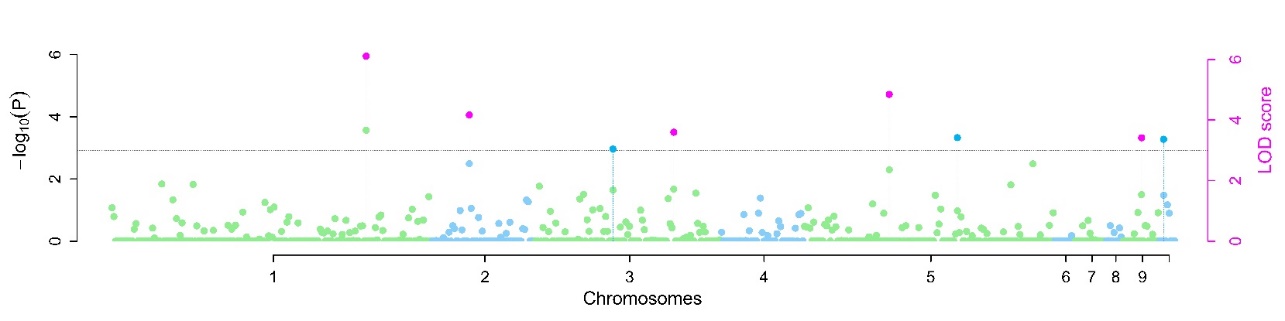


**Figure 10 Manhattan plot**

Using the P-values in Figure 10, it is easy to draw the QQ plot (Figure 11). If users do not select one of the above four approaches, the software program does not produce the QQ plot. The setups for the resolution of the QQ plot are default. If users select the format of *.pdf, the Figure width is 7 [with the unit of inches], Figure height is 7 [with the unit of inches], and Word resolution is 25 [with the unit of 1/72 inch, ppi]. If users select the other three format, Figure width is 10,000, Figure height is 10,000 [with the unit of pixel (px)], Word resolution is 60 [with the unit of 1/72 inch, being pixels per inch (ppi)], and Figure resolution is 600 [with the unit of pixels per inch (ppi)].





**Figure 11 QQ plot**

**5 Re-draw the plot according to user’s requirement**

Once users run the software package, users can obtain two result file, named ***_intermediate result.csv** and ***_Final result.csv**, which are used to redraw the Manhattan and QQ plots.

5.1 The Manhattan plot

To redraw the Manhattan plot, users first upload two Result files (*_intermediate result.csv and *_Final result.csv), and then set up the below parameters:

Manhattan plot format with four frequently used image formats: *.png, *.tiff, *.jpeg, and *.pdf;

If users select the *.png, *.tiff, or *.jpeg formats, users need to set up four parameters: 1) Figure width [with the unit of pixel (px)], 2) Figure height [with the unit of pixel (px)], 3) Word resolution [with the unit of 1/72 inch, being pixels per inch (ppi)], and 4) Figure resolution [with the unit of pixels per inch (ppi)].

If users select the *.pdf format, users need to set up three parameters: 1) Figure width [with the unit of inches], 2) Figure height [with the unit of inches], and 3) Word resolution [with the unit of 1/72 inch, ppi].

Here we give two setups for the plot resolution in the below table.

| Plot | Resolution | High resolution | | General resolution | |
| --- | --- | --- | --- | --- | --- |
|  |  | *.png, *.jpeg, & *.tiff | *.pdf | *.png, *.jpeg, & *.tiff | *.pdf |
| Manhattan | Figure width | 28,000 | 16 | 700 | 8 |
|  | Figure height | 7000 | 4 | 170 | 2 |
|  | Word resolution | 60 | 20 | 18 | 10 |
|  | Figure resolution | 600 |  | 72 |  |
| QQ | Figure width | 10,000 | 7 | 600 | 3 |
|  | Figure height | 10,000 | 7 | 600 | 3 |
|  | Word resolution | 60 | 25 | 20 | 12 |
|  | Figure resolution | 600 |  | 100 |  |

Size of all the three labels: The sizes of all the vertical and horizontal labels.

Width of all the three axes: The thickness of all the vertical and horizontal axes. When the resolution is changed from high into general, smaller thickness should be set up.

Length of tick marks: Length of axis tick marks.

Size of scale values: Size of scale values on axes.

Magnification of {-log_10_(P)}: Magnification of scale values of left vertical axis.

Magnification of {LOD score}: Magnification of scale values of right vertical axis.

Mark Genes or not: If users want to mark candidate or known genes in the

Manhattan plot, “TRUE” should be selected. If not, “FALSE” should be selected.

Under the “TRUE” situation, please input x axis, y axis, and gene name for each gene

in three text-input boxes. If multiple candidate or known genes are input, their

corresponding contents are simultaneously input, i.e., “15, 14.5” (y axis, two genes).

Note that only one color may be set up, such as “blue”, if multiple genes are marked.

Save path: Directory in your computer to save the figure.

Finally, click the “Draw Manhattan plot” button and the Manhattan plot will be produced in the Directory, which is set up by users.


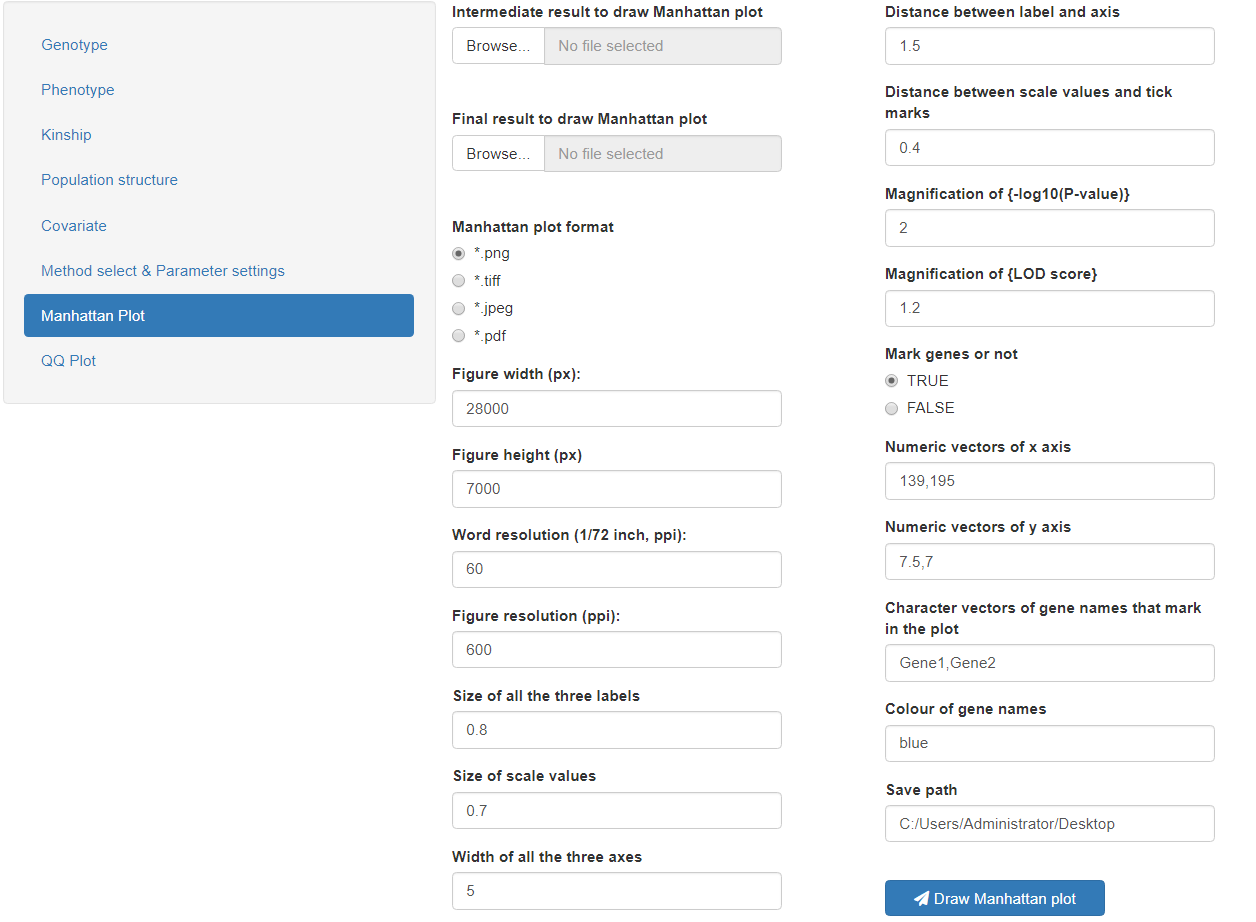


**Figure 12 Manhattan plot module**

5.2 The QQ plot

To redraw the QQ plot, users first upload one Result files (*_intermediate result.csv), and then set up the parameters, which are the same as those in the Manhattan plot, and **critical P-value of deleting points** (the default: 0.90). Finally, click the “Draw QQ plot” button and the plot will be produced in the Directory, which is set up by users.


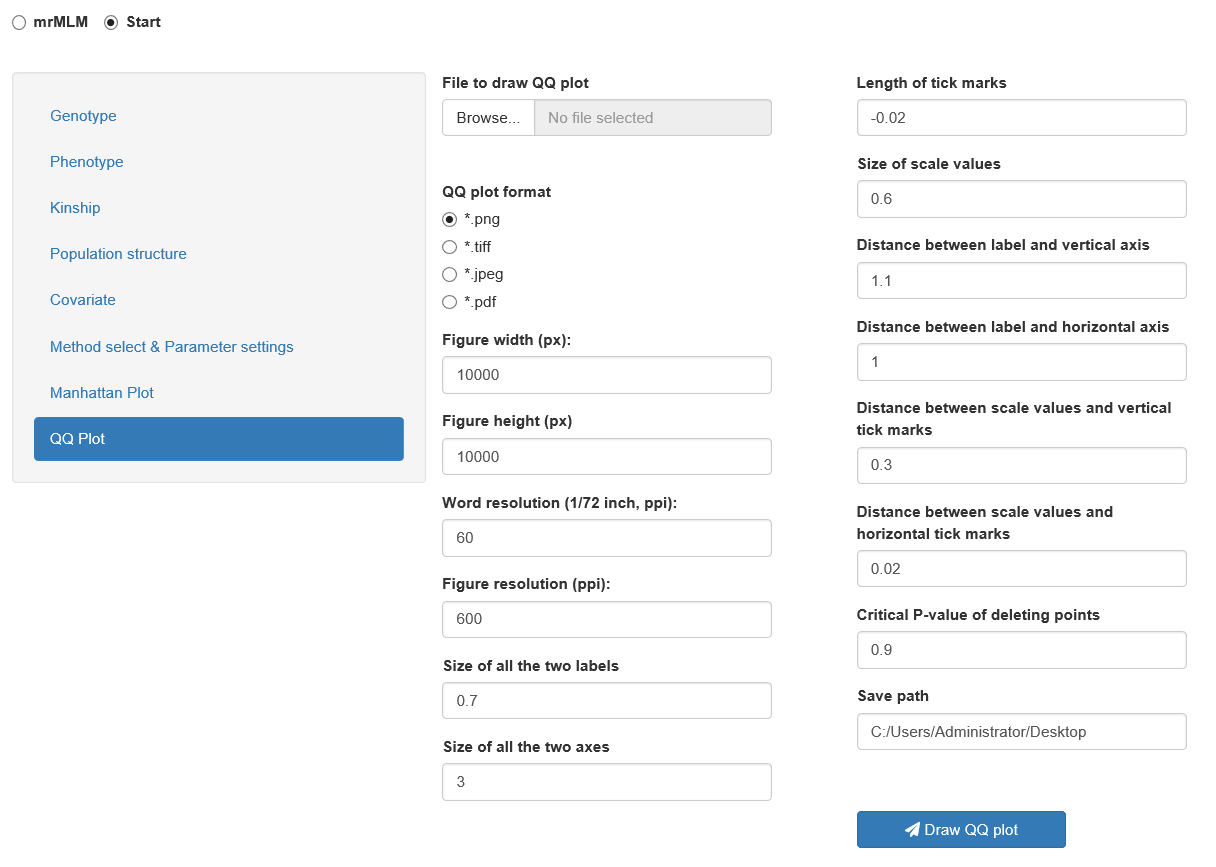


**Figure 13 QQ plot module**
