# Supplementary material for: mrMLM v4.0.2: An R Platform for Multi-locus Genome-wide Association Studies
Source: Genomics Proteomics Bioinformatics. 2020 Dec 18;18(4):481–7. doi: 10.1016/j.gpb.2020.06.006 (PMC8242264; doi:10.1016/j.gpb.2020.06.006)
Supplement: Supplementary File S5 — Real data analyses in rice, maize, and Simmental beef cattle [file mmc5.docx]

**File S5 Real data analyses in rice, maize, and Simmental beef cattle**

To test the performances of the software package mrMLM v4.0.2, three real datasets in rice [1], maize [2], and Simmental beef cattle [3] were downloaded from the Rice SNP-Seek Database (https://snp-seek.irri.org./_download.zul%3bjsessionid=B374247D2C6C9D84F6E69E77CE8C11F8), the Maizego (http://www.maizego.org/), and the Dryad Digital Repository (https://datadryad.org/stash/dataset/doi:10.5061/dryad.4qc06), respectively. In the above three datasets, the traits of interest were grain width, oil concentration, and kidney weight, respectively; the number of phenotypic accessions was 2262, 368, and 1136, respectively; the number of markers was 1.01, 1.06, and 0.67 million, respectively (File S3).

**Real data analyses in rice**

We re-analyzed the above rice dataset of the ref [1] on the second server (Intel(R) Xeon(R) Gold 6130 CPU @ 2.10GHz, 64 processors, and 629G memory). Population structure was calculated by package ADMIXTURE from rice 3K-RG core SNP (version 0.4, http://snp-seek.irri.org./_download.zul) with K = 9 [1], the first column was deleted in the GWAS, and Kinship matrix was calculated by package mrMLM v4.0.2 This dataset was re-analyzed by mrMLM, FASTmrMLM, FASTmrEMMA, pLARmEB, pKWmEB, and ISIS EM-BLASSO methods in the package mrMLM v4.0.2 (File S2). The default parameters in the above methods were used in this study.

Their total running times for the above six methods were 9.56, 3.37, 11.58, 5.09, 6.13, and 1.06 (hours), respectively. Clearly, ISIS EM-BLASSO is the least followed by FASTmrMLM, pLARmEB, pKWmEB, and mrMLM; the FASTmrEMMA is the maximum. The total numbers of QTNs significantly associated with rice grain width using the above six methods (mrMLM, FASTmrMLM, FASTmrEMMA, pLARmEB, pKWmEB, and ISIS EM-BLASSO) were 73, 77, 42, 59, 17, and 31, respectively (Table S2), indicating quite a number of QTNs (mean: 49.8) to be identified by the above multi-locus GWAS methods.

If the purpose of users is to mine candidate genes, the previously reported genes may be viewed as true genes. All the previously reported genes around the above QTNs are listed in Table S3. In addition, more important thing is to predict new candidate genes. In this case, we need to obtain all the genes around all the above QTNs and their annotations in *Oryza sativa* (<https://rapdb.dna.affrc.go.jp/download/>irgsp1.html) and *Arabidopsis thaliana* (<https://genome.jgi.doe.gov/portal/pages/>dynamicOrganismDownload.jsf?organism=Osativa). As a result, 136 genes related to seed developments had been found. All the potentially candidate genes were used to conduct KEGG analysis. Fifteen genes were associated with seed development (Table S4). We further used the datasets of gene expression levels in Nipponbare (http://rice. plantbiology.msu.edu/index.shtml), Minghui 63, and Zhenshan 97 (https://www.ncbi.nlm.nih.gov/geo/query/acc.cgi?acc=GSE19024) to mine candidate genes. As a result, *Os02g0115900* and *Os05g0182500* were predicted to be the most likely candidate genes (Table S4).

**Real data analyses in maize**

We re-analyzed the above maize dataset in the ref [2] on the third server (Intel(R) Xeon(R) CPU E5-2680 v2 @ 2.80GHz, 40 processors, and 504G memory). Population structure was calculated by package ADMIXTURE with K = 3 [2], the software automatically deleted the column whose sum is the smallest, and Kinship matrix was calculated by package mrMLM v4.0.2 This dataset was re-analyzed by mrMLM, FASTmrMLM, FASTmrEMMA, pLARmEB, pKWmEB, and ISIS EM-BLASSO methods in the package mrMLM v4.0.2 (File S2). The default parameters in the above methods were used in this study.

The total numbers of QTNs significantly associated with maize oil concentration using the above six methods were 42, 43, 31, 29, 17, and 6, respectively (Table S5), indicating quite a number of QTNs (mean: 28) to be identified by the above multi-locus GWAS methods.

In order to mine candidate genes that are significantly associated with oil concentration in maize, we obtained all the genes around all the above QTNs and got their annotations in *Zea mays* (<http://www.gramene.org/> and <http://ensembl.gramene.org/Zea_mays/Info/Index>) and *Arabidopsis thaliana* (<https://www.arabidopsis.org/>). All the candidate genes around the above QTNs are listed in Table S6. As a result, 23 genes were found to be associated with oil concentration. Among these genes, 13 were detected only by the software mrMLM, and 10 were detected commonly by the mrMLM software and in the ref [2].

**Real data analyses in** **Simmental beef cattle**

We re-analyzed the above Simmental beef cattle dataset in the ref [3] on the second server (Intel(R) Xeon(R) Gold 6130 CPU @ 2.10GHz, 64 processors, and 629 G memory). R function prcomp() was used to conduct principal component analysis. As shown in Figure 1, all the lines were clustered into five groups, in other words, there were five sub-populations in this association population. The five principal components were used to correct the effect of population structure on GWAS for kidney weight in Simmental beef cattle.

| 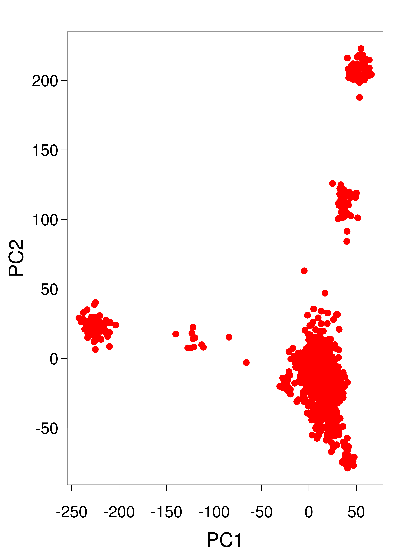 | **Figure 1** **Principal component analysis for the genotypes in Simmental beef cattle**  The dataset was derived from the ref [3]. |
| --- | --- |

The software mrMLM v4.0.2 was used to calculate Kinship matrix. In this dataset, there were four covariates, including gender (categorical variable), birth year (categorical variable), body weight before experiment (continuous variable), and the number of days during fattening period (continuous variable). The last two continuous covariates were transferred into categorical variables by clustering them into five and six clusters, respectively. This dataset was re-analyzed by mrMLM, FASTmrMLM, FASTmrEMMA, pLARmEB, pKWmEB, and ISIS EM-BLASSO in the software mrMLM v4.0.2 (File S2).

The total numbers of QTNs, detected by the above methods, for kidney weight in Simmental beef cattle were 4, 55, 167, 117, 8, and 48, respectively (Table S7), indicating quite a number of QTNs (mean: 66.5) to be identified by the above multi-locus GWAS methods.

In order to mine candidate genes that are significantly associated with kidney weight, we obtained all the genes around all the detected QTNs and got their annotations in *Bos taurus* (<http://oct2018.archive.ensembl.org/Bos_taurus/Info/Index?db=core>). All the candidate genes around the above QTNs are listed in Table S8. Among the previously reported genes, *MECOM* was identified commonly by mrMLM and in the ref [4], and *LCORL* and *NCAPG*, which are very important genes for kidney weight in cattle, were detected only by the mrMLM (Table S8).

**Influence of various factors on QTN detection using mrMLM v4.0.2**

In testing the performances of the software package mrMLM v4.0.2, the full and reduced real datasets in rice [1] were analyzed in four experiments. The first experiment was conducted on the first server (Intel(R) Xeon(R) CPU x5670@2.93GHz, 20 processors, and 141 G memory), the second experiment was conducted on the second server (Intel(R) Xeon(R) Gold 6130 CPU @ 2.10GHz, 64 processors, and 629 G memory), the third experiment was conducted on the third server (Intel(R) Xeon(R)CPU E5-2680 v2 @ 2.80GHz, 40 processors, and 504 G memory), and the fourth experiment was conducted on the fourth server (Intel(R) Xeon(R)CPU E5-2637 v2 @3.50GHz, 16 processors, and 504 G memory).

**References**

1. [Wang W](https://www.ncbi.nlm.nih.gov/pubmed/?term=Wang%20W%5BAuthor%5D&cauthor=true&cauthor_uid=29695866), [Mauleon R](https://www.ncbi.nlm.nih.gov/pubmed/?term=Mauleon%20R%5BAuthor%5D&cauthor=true&cauthor_uid=29695866), [Hu Z](https://www.ncbi.nlm.nih.gov/pubmed/?term=Hu%20Z%5BAuthor%5D&cauthor=true&cauthor_uid=29695866), [Chebotarov D](https://www.ncbi.nlm.nih.gov/pubmed/?term=Chebotarov%20D%5BAuthor%5D&cauthor=true&cauthor_uid=29695866), [Tai S](https://www.ncbi.nlm.nih.gov/pubmed/?term=Tai%20S%5BAuthor%5D&cauthor=true&cauthor_uid=29695866), [Wu Z](https://www.ncbi.nlm.nih.gov/pubmed/?term=Wu%20Z%5BAuthor%5D&cauthor=true&cauthor_uid=29695866), et al. Genomic variation in 3,010 diverse accessions of Asian cultivated rice. [Nature](https://www.ncbi.nlm.nih.gov/pubmed/?term=Genomic+variation+in+3%2C010+diverse+accessions+of+Asian+cultivated+rice) 2018;557:43–9.
2. [Li H](https://www.ncbi.nlm.nih.gov/pubmed/?term=Li%20H%5BAuthor%5D&cauthor=true&cauthor_uid=23242369), [Peng Z](https://www.ncbi.nlm.nih.gov/pubmed/?term=Peng%20Z%5BAuthor%5D&cauthor=true&cauthor_uid=23242369), [Yang X](https://www.ncbi.nlm.nih.gov/pubmed/?term=Yang%20X%5BAuthor%5D&cauthor=true&cauthor_uid=23242369), [Wang W](https://www.ncbi.nlm.nih.gov/pubmed/?term=Wang%20W%5BAuthor%5D&cauthor=true&cauthor_uid=23242369), [Fu J](https://www.ncbi.nlm.nih.gov/pubmed/?term=Fu%20J%5BAuthor%5D&cauthor=true&cauthor_uid=23242369), [Wang J](https://www.ncbi.nlm.nih.gov/pubmed/?term=Wang%20J%5BAuthor%5D&cauthor=true&cauthor_uid=23242369), et al. Genome-wide association study dissects the genetic architecture of oil biosynthesis in maize kernels. [Nat Genet](https://www.ncbi.nlm.nih.gov/pubmed/?term=Genome-wide+association+study+dissects+the+genetic+architecture+of+oil+biosynthesis+in+maize+kernels) 2013;45:43–50.
3. [Zhu B](https://www.ncbi.nlm.nih.gov/pubmed/?term=Zhu%20B%5BAuthor%5D&cauthor=true&cauthor_uid=27139889), [Zhu M](https://www.ncbi.nlm.nih.gov/pubmed/?term=Zhu%20M%5BAuthor%5D&cauthor=true&cauthor_uid=27139889), [Jiang J](https://www.ncbi.nlm.nih.gov/pubmed/?term=Jiang%20J%5BAuthor%5D&cauthor=true&cauthor_uid=27139889), [Niu H](https://www.ncbi.nlm.nih.gov/pubmed/?term=Niu%20H%5BAuthor%5D&cauthor=true&cauthor_uid=27139889), [Wang Y](https://www.ncbi.nlm.nih.gov/pubmed/?term=Wang%20Y%5BAuthor%5D&cauthor=true&cauthor_uid=27139889), [Wu Y](https://www.ncbi.nlm.nih.gov/pubmed/?term=Wu%20Y%5BAuthor%5D&cauthor=true&cauthor_uid=27139889), et al. The impact of variable degrees of freedom and scale parameters in Bayesian methods for genomic prediction in Chinese Simmental beef cattle. [PLoS One](https://www.ncbi.nlm.nih.gov/pubmed/?term=The+Impact+of+Variable+Degrees+of+Freedom+and+Scale+Parameters+in+Bayesian+Methods+for+Genomic+Prediction+in+Chinese+Simmental+Beef+Cattle) 2016;11:e0154118.
4. An B, Xia J, Chang T, Wang X, Miao J, Xu L, et al. Genome-wide association study identifies loci and candidate genes for internal organ weights in Simmental beef cattle. Physiol Genomics 2018;50:523–31.
