# Supplementary material for: mrMLM v4.0.2: An R Platform for Multi-locus Genome-wide Association Studies
Source: Genomics Proteomics Bioinformatics. 2020 Dec 18;18(4):481–7. doi: 10.1016/j.gpb.2020.06.006 (PMC8242264; doi:10.1016/j.gpb.2020.06.006)
Supplement: Supplementary File S6 — Monte Carlo simulation experiments [file mmc6.docx]

**File S6 Monte Carlo simulation experiments**

All the simulation datasets used in this study were downloaded from the Dryad Digital Repository ([http://dx.doi.org/10.5061/dryad.sk652) [1]](http://dx.doi.org/10.5061/dryad.sk652)%20%5b1%5d) and re-analyzed to validate the software package. Here we simply described them. Sample size was 199, the number of markers was 10,000, six QTNs were simulated on marker positions, and the number of replicates was 1000. For each simulated QTN, we counted the samples in which the LOD scores exceeded 3.0 for our multi-locus methods, and the P-value was less than 0.05/*m* for others. In the first, second, and third simulation experiments, the phenotypic values were simulated by the models $\mathbf{y}=\mathbf{\mu}+\sum_{k=1}^{6} \mathbf{x}_{k}b_{k}+\mathbf{\varepsilon}$, $\mathbf{y}=\mathbf{\mu}+\sum_{k=1}^{6} \mathbf{x}_{k}b_{k}+\mathbf{u}\text{+}\mathbf{\varepsilon}$ , and $\mathbf{y}=\mathbf{\mu}+\sum_{k=1}^{6} \mathbf{x}_{k}b_{k}+$ $\sum_{l=1}^{3} \left(\text{A}_{l}\#\text{B}_{l}\right)b_{ll}\text{+}\mathbf{\varepsilon}$, respectively, where polygenic effect $\mathbf{u}\text{\textasciitilde MVN}_{n}\text{(}0,2\times\mathbf{K}\text{)}$, residual effect $\mathbf{\varepsilon}\text{\textasciitilde MVN}_{n}\text{(}0,10\times\mathbf{I}\text{)}$, $b_{k}$ is QTN effect ($k=1,\cdots,6$), $b_{ll}$ is epistatic effect ($l=1,\cdots,3$),$\text{A}_{l}\#\text{B}_{l}$ is the incidence coefficient of epistatic effect, and $\mathbf{K}$ is the kinship matrix between a pair of individuals.

**References**

[1] Zhang J, Feng JY, Ni YL, Wen YJ, Niu Y, Tamba CL, et al. pLARmEB: integration of least angle regression with empirical Bayes for multilocus genome-wide association studies. Heredity 2017;118:517–24.
