# Supplementary material for: mrMLM v4.0.2: An R Platform for Multi-locus Genome-wide Association Studies
Source: Genomics Proteomics Bioinformatics. 2020 Dec 18;18(4):481–7. doi: 10.1016/j.gpb.2020.06.006 (PMC8242264; doi:10.1016/j.gpb.2020.06.006)
Supplement: Supplementary Figure S1 — Methodological comparison in mrMLM v4.0.2 [file mmc7.pdf]

Genetic model

ISIS EM-BLASSO $y = W\alpha + \sum_{\text{Each chr.}} x_i\beta_i + Zu + \varepsilon$

pLARmEB $y = W\alpha + \sum_{\text{All markers}} x_i\beta_i + \varepsilon$

Other four methods $y = W\alpha + x\beta + Zu + \varepsilon$

SNP effects for all the six methods: random

Model transformation

mrMLM: $y^* = U^T y$, where kinship matrix $K = UDU^T$;

FASTmrMLM: $y^* = R^{-1/2} U^T y$, where $R = \lambda_g D + I_n$ and λ_g is polygenic-to-residual variance ratio;

FASTmrEMMA, pLARmEB, and pKWmEB: $y^* = Cy$

where $C = Q_1 \Lambda^{-1/2} Q_1^T$, $B = \hat{\lambda}_g ZKZ^T + I_n = \left(Q_1 \Lambda^{1/2} Q_1^T\right)\left(Q_1 \Lambda^{1/2} Q_1^T\right)$, and $Var(y^*) = \sigma_e^2 \left(\hat{\lambda}_\beta X_C X_C^T + I_n\right)$.

Selection of potentially associated SNPs

mrMLM, FASTmrMLM (REML), FASTmrEMMA (ML & REML), and pKWmEB (Kruskal-Wallis test): single-locus genome scan under population structure and polygenic background controls;

pLARmEB: least angle regression is used to select potentially associated SNPs from all the markers on each chromosome;

ISIS EM-BLASSO: the modified SIS and SCAD methods are used to select potentially associated SNPs from all the markers on the genome.

True QTN identification in multi-locus model

All the selected SNPs are placed into one multi-locus genetic model.

Their effects are estimated by empirical Bayes or EM Bayesian LASSO (PMID: 20051978).

All the non-zero effects are further identified by likelihood ratio test for the detection of true QTNs with the LOD score ≥ 3.0 as significant.
