# Supplementary material for: mrMLM v4.0.2: An R Platform for Multi-locus Genome-wide Association Studies
Source: Genomics Proteomics Bioinformatics. 2020 Dec 18;18(4):481–7. doi: 10.1016/j.gpb.2020.06.006 (PMC8242264; doi:10.1016/j.gpb.2020.06.006)
Supplement: Supplementary Figure S2 — Comparison of powers in QTN detection between the new and existing methods in three simulation experiments A. The first simulation experiment. B. The second simulation experiment. C. The third simulation experiment. The new methods include our multi-locus GWAS methods, while the existing methods include GEMMA, EMMAX, and FarmCPU. QTN, quantitative trait nucleotide. [file mmc8.pdf]

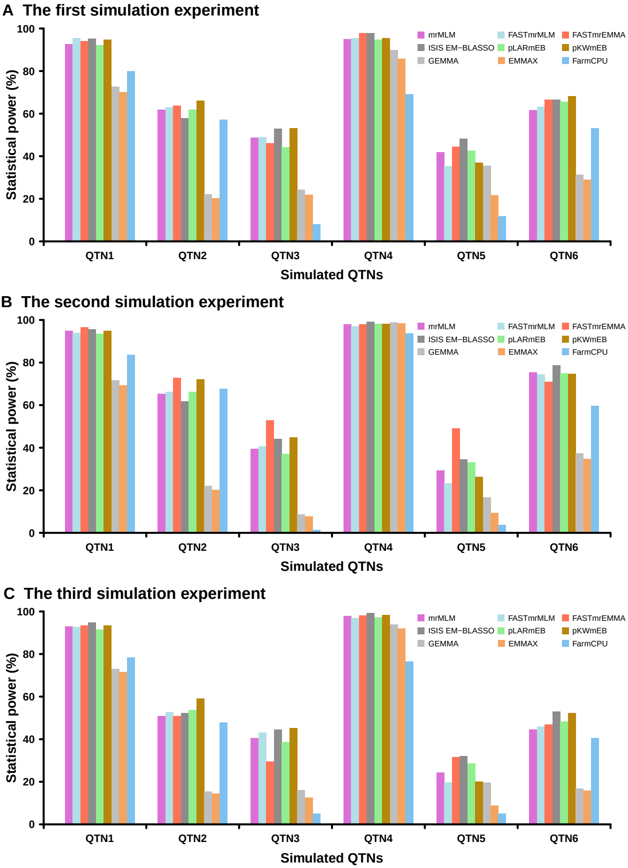
A The first simulation experiment
B The second simulation experiment
C The third simulation experiment
Statistical power (%)
Simulated QTNs
QTN1
QTN2
QTN3
QTN4
QTN5
QTN6
mrMLM
FASTmrMLM
FASTmrEMMA
ISIS EM−BLASSO
pLARmEB
pKWmEB
GEMMA
EMMAX
FarmCPU
0
20
40
60
80
100
