# Supplementary material for: mrMLM v4.0.2: An R Platform for Multi-locus Genome-wide Association Studies
Source: Genomics Proteomics Bioinformatics. 2020 Dec 18;18(4):481–7. doi: 10.1016/j.gpb.2020.06.006 (PMC8242264; doi:10.1016/j.gpb.2020.06.006)
Supplement: Supplementary Figure S4 — Comparison of false positive rates (%) in QTN detection between the new and existing methods in three simulation experiments A. The first simulation experiment. B. The second simulation experiment. C. The third simulation experiment. The new methods include our multi-locus GWAS methods, while the existing methods include GEMMA, EMMAX, and FarmCPU. QTN, quantitative trait nucleotide. [file mmc10.pdf]

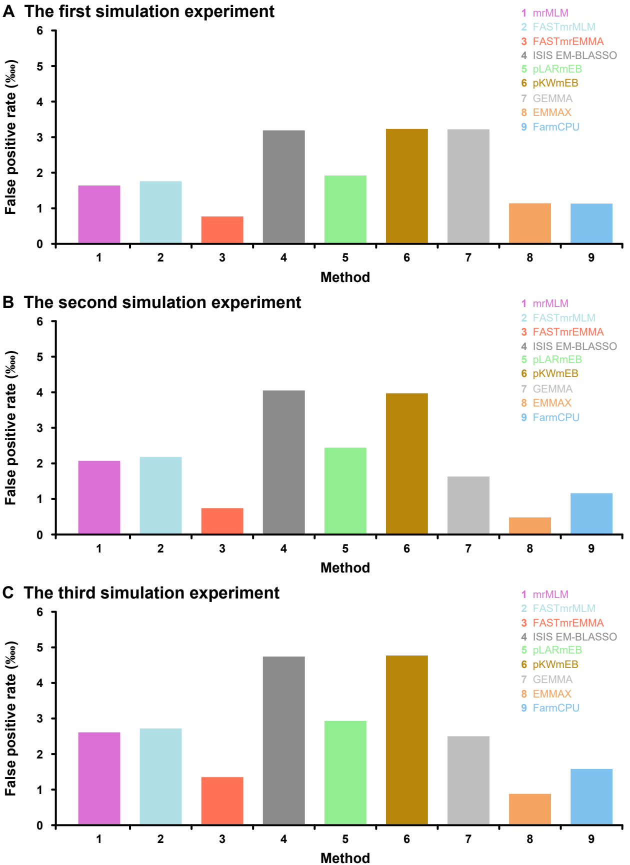

A The first simulation experiment
B The second simulation experiment
C The third simulation experiment
False positive rate (‱)
Method
0
1
2
3
4
5
6
7
8
9
1 mrMLM
2 FASTmrMLM
3 FASTmrEMMA
4 ISIS EM-BLASSO
5 pLARmEB
6 pKWmEB
7 GEMMA
8 EMMAX
9 FarmCPU
