# Supplementary material for: mrMLM v4.0.2: An R Platform for Multi-locus Genome-wide Association Studies
Source: Genomics Proteomics Bioinformatics. 2020 Dec 18;18(4):481–7. doi: 10.1016/j.gpb.2020.06.006 (PMC8242264; doi:10.1016/j.gpb.2020.06.006)
Supplement: Supplementary Table S1 — The speedup in parallel computing under various numbers of CPUs and various GWAS approaches [file mmc11.docx]

**Table S1 The speedup in parallel computing under various numbers of CPUs and various GWAS approaches**

| **Number of CPUs** | **Multi-locus GWAS methods** | | | | | |
| --- | --- | --- | --- | --- | --- | --- |
|  | **mrMLM** | **FASTmrMLM** | **FASTmrEMMA** | **pLARmEB** | **pKWmEB** | **ISIS EM-BLASSO** |
| 1 | 1.00 | 1.00 | 1.00 | 1.00 | 1.00 | 1.00 |
| 2 | 1.65 | 1.66 | 1.90 | 1.85 | 1.91 | 1.95 |
| 3 | 2.07 | 2.20 | 2.40 | 2.45 | 2.79 | 2.31 |
| 4 | 2.45 | 2.64 | 2.89 | 2.88 | 3.38 | 2.74 |
| 5 | 3.10 | 3.24 | 3.26 | 2.66 | 3.89 | 2.78 |
| 6 | 3.23 | 3.42 | 3.56 | 2.69 | 4.31 | 2.74 |
| 7 | 3.52 | 3.58 | 3.83 | 2.64 | 4.90 | 2.69 |
