# Supplementary material for: mrMLM v4.0.2: An R Platform for Multi-locus Genome-wide Association Studies
Source: Genomics Proteomics Bioinformatics. 2020 Dec 18;18(4):481–7. doi: 10.1016/j.gpb.2020.06.006 (PMC8242264; doi:10.1016/j.gpb.2020.06.006)
Supplement: Supplementary Table S2 — All the QTNs for grain width in rice detected by our multi-locus GWAS methods [file mmc12.docx]

**Table S2 All the QTNs for grain width in rice detected by our multi-locus GWAS methods**

| Chr | Position (bp) | QTN effect | LOD score | –Log_10_ *P* | r^2^ (%) | Method | Chr | Position (bp) | QTN effect | LOD score | –Log_10_ *P* | r^2^ (%) | Method |
| --- | --- | --- | --- | --- | --- | --- | --- | --- | --- | --- | --- | --- | --- |
| 1 | 6921155 | 0.0388 | 5.37 | 6.18 | 0.665 | mrMLM | 1 | 1831596 | -0.0354 | 3.19 | 3.90 | 0.1383 | FASTmrEMMA |
| 1 | 1831596 | -0.0331 | 4.79 | 5.57 | 0.5442 | mrMLM | 1 | 6491279 | -0.0798 | 5.38 | 6.20 | 0.836 | FASTmrEMMA |
| 1 | 23986872 | -0.0237 | 4.15 | 4.91 | 0.2814 | mrMLM | 1 | 10206576 | -0.0502 | 3.31 | 4.03 | 0.2181 | FASTmrEMMA |
| 1 | 41933338 | -0.0678 | 5.19 | 6.00 | 1.1884 | mrMLM | 1 | 39242548 | -0.0856 | 4.88 | 5.67 | 0.9135 | FASTmrEMMA |
| 1 | 19591075 | 0.0627 | 3.91 | 4.65 | 1.2254 | mrMLM | 1 | 41933338 | -6.38E-05 | 3.28 | 3.99 | 1.51E-07 | FASTmrEMMA |
| 2 | 33721702 | -0.0385 | 4.76 | 5.55 | 0.3686 | mrMLM | 2 | 9896989 | -0.093 | 6.57 | 7.42 | 0.6323 | FASTmrEMMA |
| 2 | 24640538 | 0.0269 | 3.45 | 4.17 | 0.2932 | mrMLM | 2 | 21908502 | -0.1073 | 5.95 | 6.78 | 1.4443 | FASTmrEMMA |
| 3 | 29849519 | 0.2325 | 13.02 | 14.02 | 1.5649 | mrMLM | 2 | 33773893 | -0.0859 | 3.15 | 3.86 | 1.0211 | FASTmrEMMA |
| 3 | 16733441 | -0.0475 | 12.00 | 12.98 | 1.2026 | mrMLM | 3 | 7230027 | -0.0679 | 4.21 | 4.97 | 0.5041 | FASTmrEMMA |
| 3 | 4919241 | 0.0512 | 3.70 | 4.43 | 0.9376 | mrMLM | 3 | 16733441 | -0.0603 | 5.01 | 5.81 | 0.4777 | FASTmrEMMA |
| 3 | 35769449 | 0.025 | 4.65 | 5.44 | 0.349 | mrMLM | 3 | 25332258 | 0.0628 | 4.70 | 5.48 | 0.478 | FASTmrEMMA |
| 3 | 7230027 | -0.0442 | 4.69 | 5.47 | 0.8871 | mrMLM | 3 | 35769449 | 0.0415 | 3.93 | 4.68 | 0.2303 | FASTmrEMMA |
| 3 | 26707321 | -0.0272 | 3.78 | 4.52 | 0.3492 | mrMLM | 4 | 1563119 | 0.0882 | 8.07 | 8.96 | 0.5889 | FASTmrEMMA |
| 4 | 837855 | 0.0538 | 4.99 | 5.78 | 0.7654 | mrMLM | 4 | 31705285 | -0.0662 | 4.20 | 4.96 | 0.414 | FASTmrEMMA |
| 4 | 32080864 | 0.0371 | 3.91 | 4.66 | 0.7407 | mrMLM | 4 | 32080864 | 0.0869 | 5.86 | 6.69 | 0.641 | FASTmrEMMA |
| 4 | 31705285 | -0.0335 | 4.03 | 4.78 | 0.4558 | mrMLM | 5 | 4833479 | -0.0724 | 4.25 | 5.02 | 0.5812 | FASTmrEMMA |
| 4 | 2404846 | 0.0392 | 3.56 | 4.29 | 0.4954 | mrMLM | 5 | 5361276 | -0.1108 | 3.29 | 4.00 | 1.595 | FASTmrEMMA |
| 4 | 10435637 | 0.0539 | 3.65 | 4.39 | 0.8709 | mrMLM | 5 | 5376243 | 0.2849 | 10.81 | 11.77 | 6.7119 | FASTmrEMMA |
| 4 | 22092126 | 0.0464 | 3.85 | 4.59 | 0.5928 | mrMLM | 5 | 5383914 | -0.1003 | 3.34 | 4.05 | 0.9649 | FASTmrEMMA |
| 5 | 5308999 | 0.0999 | 21.51 | 22.61 | 3.4461 | mrMLM | 5 | 27897307 | 0.0675 | 5.16 | 5.96 | 0.307 | FASTmrEMMA |
| 5 | 5007414 | -0.0662 | 12.60 | 13.59 | 2.011 | mrMLM | 6 | 1179404 | -0.0524 | 5.91 | 6.74 | 0.3495 | FASTmrEMMA |
| 5 | 5422688 | -0.0334 | 4.93 | 5.73 | 0.6056 | mrMLM | 6 | 27500790 | 0.0918 | 3.57 | 4.29 | 0.2549 | FASTmrEMMA |
| 5 | 27902614 | -0.1072 | 10.87 | 11.82 | 1.3452 | mrMLM | 7 | 16169282 | -0.0706 | 5.85 | 6.68 | 0.4404 | FASTmrEMMA |
| 5 | 5656974 | -0.0902 | 4.50 | 5.28 | 0.4653 | mrMLM | 7 | 19316334 | -0.0673 | 5.92 | 6.75 | 0.6084 | FASTmrEMMA |
| 5 | 5606561 | -0.0406 | 4.57 | 5.34 | 0.8252 | mrMLM | 7 | 21323336 | 0.0395 | 3.36 | 4.07 | 0.1957 | FASTmrEMMA |
| 5 | 762855 | -0.0453 | 4.03 | 4.78 | 0.7378 | mrMLM | 7 | 21666972 | 0.0822 | 4.78 | 5.57 | 0.9378 | FASTmrEMMA |
| 5 | 3627700 | 0.0376 | 3.95 | 4.70 | 0.6338 | mrMLM | 7 | 28321075 | 0.1372 | 8.42 | 9.32 | 0.9812 | FASTmrEMMA |
| 5 | 13136371 | -0.0563 | 7.23 | 8.10 | 1.4307 | mrMLM | 7 | 29606768 | 0.0523 | 3.34 | 4.06 | 0.2785 | FASTmrEMMA |
| 5 | 23667256 | -0.0454 | 3.66 | 4.39 | 0.5525 | mrMLM | 8 | 861782 | -0.058 | 4.86 | 5.65 | 0.3535 | FASTmrEMMA |
| 6 | 6346438 | 0.0883 | 4.78 | 5.57 | 0.3203 | mrMLM | 8 | 17843449 | -0.0618 | 4.42 | 5.19 | 0.5114 | FASTmrEMMA |
| 6 | 1179404 | -0.0273 | 4.56 | 5.34 | 0.4377 | mrMLM | 8 | 26217209 | 0.0521 | 4.85 | 5.64 | 0.2712 | FASTmrEMMA |
| 6 | 17265592 | -0.0384 | 5.06 | 5.86 | 0.8582 | mrMLM | 9 | 4595714 | -0.071 | 4.29 | 5.05 | 0.5662 | FASTmrEMMA |
| 6 | 30550157 | 0.0343 | 3.24 | 3.95 | 0.6941 | mrMLM | 9 | 6751029 | 0.0418 | 3.04 | 3.74 | 0.2434 | FASTmrEMMA |
| 7 | 29606768 | 0.0361 | 3.68 | 4.42 | 0.7402 | mrMLM | 9 | 8525240 | -0.048 | 4.07 | 4.83 | 0.2603 | FASTmrEMMA |
| 7 | 23624287 | 0.0368 | 5.21 | 6.01 | 0.4902 | mrMLM | 9 | 21359117 | -0.1137 | 9.10 | 10.02 | 1.2987 | FASTmrEMMA |
| 7 | 1579705 | -0.0523 | 4.70 | 5.48 | 0.7191 | mrMLM | 10 | 9280914 | -0.0837 | 4.70 | 5.48 | 0.447 | FASTmrEMMA |
| 7 | 21780181 | 0.0471 | 5.85 | 6.67 | 1.3002 | mrMLM | 10 | 19947905 | -0.1627 | 10.67 | 11.62 | 1.149 | FASTmrEMMA |
| 7 | 28456965 | 0.0412 | 3.85 | 4.60 | 0.8719 | mrMLM | 11 | 6915344 | 0.065 | 6.74 | 7.60 | 0.5872 | FASTmrEMMA |
| 8 | 26504638 | 0.1537 | 12.33 | 13.31 | 0.924 | mrMLM | 11 | 8857734 | 0.0529 | 3.57 | 4.30 | 0.1645 | FASTmrEMMA |
| 8 | 26249593 | 0.0654 | 3.03 | 3.73 | 0.2659 | mrMLM | 11 | 15133268 | -0.0885 | 6.48 | 7.33 | 0.4558 | FASTmrEMMA |
| 8 | 1660498 | 0.0526 | 3.05 | 3.75 | 1.6254 | mrMLM | 11 | 19526177 | -0.1052 | 6.69 | 7.54 | 0.4356 | FASTmrEMMA |
| 8 | 26019239 | -0.0414 | 3.58 | 4.31 | 0.5862 | mrMLM | 11 | 27020790 | 0.0506 | 4.59 | 5.37 | 0.2833 | FASTmrEMMA |
| 8 | 24755914 | -0.0297 | 3.50 | 4.22 | 0.4746 | mrMLM | 1 | 1831596 | -0.0183 | 3.26 | 3.97 | 0.1475 | pLARmEB |
| 8 | 5967659 | 0.0305 | 3.11 | 3.81 | 0.3142 | mrMLM | 1 | 26469542 | -0.0537 | 5.27 | 6.08 | 0.2973 | pLARmEB |
| 8 | 7641402 | -0.0295 | 3.07 | 3.77 | 0.496 | mrMLM | 1 | 31772747 | -0.0516 | 7.05 | 7.91 | 0.4934 | pLARmEB |
| 8 | 3881704 | -0.0577 | 6.20 | 7.04 | 1.3385 | mrMLM | 1 | 34597034 | 0.033 | 4.37 | 5.14 | 0.3379 | pLARmEB |
| 9 | 11745151 | -0.0688 | 3.50 | 4.23 | 0.2423 | mrMLM | 1 | 40027771 | 0.0589 | 4.10 | 4.85 | 0.258 | pLARmEB |
| 9 | 21393671 | -0.082 | 11.22 | 12.18 | 3.054 | mrMLM | 1 | 41933338 | -0.0451 | 3.90 | 4.64 | 0.4652 | pLARmEB |
| 9 | 15024826 | -0.0589 | 5.20 | 6.01 | 1.5083 | mrMLM | 2 | 11022186 | 0.0413 | 4.73 | 5.51 | 0.4998 | pLARmEB |
| 9 | 4905187 | 0.0424 | 4.31 | 5.08 | 0.8076 | mrMLM | 2 | 20311039 | -0.0175 | 4.03 | 4.78 | 0.1476 | pLARmEB |
| 9 | 10309830 | 0.0429 | 5.20 | 6.00 | 0.6999 | mrMLM | 2 | 33773893 | -0.0411 | 5.45 | 6.27 | 0.8569 | pLARmEB |
| 9 | 5861022 | 0.0551 | 4.91 | 5.71 | 0.7963 | mrMLM | 3 | 3676174 | -0.0705 | 5.25 | 6.05 | 0.5537 | pLARmEB |
| 9 | 21658026 | -0.0366 | 4.84 | 5.63 | 0.7688 | mrMLM | 3 | 5242456 | 0.0778 | 5.86 | 6.69 | 0.5017 | pLARmEB |
| 9 | 10501606 | -0.0474 | 4.35 | 5.12 | 0.9328 | mrMLM | 3 | 6865287 | 0.0251 | 5.98 | 6.81 | 0.2917 | pLARmEB |
| 9 | 6765802 | 0.0399 | 3.27 | 3.98 | 0.8269 | mrMLM | 3 | 16733441 | -0.0281 | 6.23 | 7.07 | 0.3728 | pLARmEB |
| 9 | 8525240 | -0.0294 | 3.52 | 4.25 | 0.4968 | mrMLM | 3 | 25456496 | 0.0311 | 3.17 | 3.87 | 0.1134 | pLARmEB |
| 9 | 7015224 | -0.0629 | 7.65 | 8.53 | 1.4863 | mrMLM | 3 | 35268655 | 0.0328 | 3.96 | 4.71 | 0.4187 | pLARmEB |
| 9 | 7648345 | 0.0305 | 3.02 | 3.71 | 0.5332 | mrMLM | 4 | 280312 | -0.065 | 5.06 | 5.86 | 0.8112 | pLARmEB |
| 10 | 19946757 | -0.0942 | 16.40 | 17.4 | 1.5924 | mrMLM | 4 | 5115500 | 0.0579 | 5.16 | 5.97 | 0.8066 | pLARmEB |
| 10 | 9282515 | -0.0368 | 3.90 | 4.64 | 0.514 | mrMLM | 4 | 13764697 | 0.0533 | 4.97 | 5.77 | 0.8885 | pLARmEB |
| 10 | 14652991 | 0.0433 | 3.51 | 4.24 | 1.0358 | mrMLM | 4 | 20536944 | -0.0231 | 3.08 | 3.78 | 0.244 | pLARmEB |
| 11 | 26988796 | 0.0284 | 5.10 | 5.90 | 0.397 | mrMLM | 4 | 31705285 | -0.0303 | 5.80 | 6.63 | 0.3291 | pLARmEB |
| 11 | 24873792 | -0.0287 | 4.65 | 5.43 | 0.4436 | mrMLM | 4 | 32238154 | 0.0425 | 4.56 | 5.34 | 0.4813 | pLARmEB |
| 11 | 19526177 | -0.0552 | 5.42 | 6.24 | 0.9821 | mrMLM | 5 | 972649 | -0.0431 | 3.32 | 4.04 | 0.8471 | pLARmEB |
| 11 | 693197 | -0.0452 | 5.57 | 6.39 | 0.9263 | mrMLM | 5 | 4859223 | -0.0402 | 8.18 | 9.08 | 0.7821 | pLARmEB |
| 11 | 8857734 | 0.0447 | 7.23 | 8.10 | 0.3815 | mrMLM | 5 | 5361276 | -0.0507 | 6.81 | 7.67 | 1.293 | pLARmEB |
| 11 | 10933886 | -0.0453 | 6.67 | 7.52 | 1.2151 | mrMLM | 5 | 5371949 | -0.0443 | 6.11 | 6.95 | 1.0271 | pLARmEB |
| 11 | 22303236 | -0.0223 | 3.72 | 4.45 | 0.2924 | mrMLM | 5 | 5376243 | 0.0771 | 11.60 | 12.57 | 1.8483 | pLARmEB |
| 11 | 22817547 | 0.0478 | 7.58 | 8.46 | 1.309 | mrMLM | 5 | 27902614 | -0.084 | 8.38 | 9.28 | 0.7336 | pLARmEB |
| 12 | 15690082 | -0.0502 | 4.19 | 4.95 | 0.9448 | mrMLM | 6 | 27500790 | 0.0593 | 3.36 | 4.08 | 1.2362 | pLARmEB |
| 12 | 9405542 | 0.0542 | 4.36 | 5.12 | 0.881 | mrMLM | 6 | 29387919 | -0.0352 | 3.20 | 3.90 | 0.3742 | pLARmEB |
| 12 | 19906987 | 0.0501 | 4.93 | 5.73 | 1.4848 | mrMLM | 7 | 9422683 | 0.0578 | 4.27 | 5.04 | 0.3554 | pLARmEB |
| 12 | 21802207 | -0.0547 | 6.83 | 7.69 | 1.0939 | mrMLM | 7 | 16169282 | -0.0324 | 5.53 | 6.35 | 0.4693 | pLARmEB |
| 1 | 2090441 | 0.0276 | 3.64 | 4.37 | 0.3867 | FASTmrMLM | 7 | 18257805 | 0.022 | 4.03 | 4.78 | 0.1868 | pLARmEB |
| 1 | 6921155 | 0.0323 | 5.67 | 6.49 | 0.4608 | FASTmrMLM | 7 | 21685804 | 0.0726 | 6.32 | 7.17 | 0.382 | pLARmEB |
| 1 | 10206576 | -0.0265 | 3.20 | 3.91 | 0.2369 | FASTmrMLM | 7 | 23624287 | 0.0323 | 4.73 | 5.51 | 0.3354 | pLARmEB |
| 1 | 29644733 | 0.0743 | 3.45 | 4.17 | 0.3047 | FASTmrMLM | 7 | 27677635 | -0.022 | 3.27 | 3.98 | 0.241 | pLARmEB |
| 1 | 39410519 | -0.0377 | 3.72 | 4.46 | 0.8305 | FASTmrMLM | 7 | 29606768 | 0.0289 | 5.35 | 6.16 | 0.4213 | pLARmEB |
| 1 | 40315934 | 0.0201 | 3.75 | 4.48 | 0.2392 | FASTmrMLM | 8 | 7641402 | -0.0222 | 3.28 | 3.99 | 0.2482 | pLARmEB |
| 1 | 41275767 | -0.0308 | 7.59 | 8.47 | 0.5615 | FASTmrMLM | 8 | 15609316 | 0.0381 | 6.52 | 7.37 | 0.5221 | pLARmEB |
| 1 | 41933338 | -0.058 | 5.34 | 6.15 | 0.868 | FASTmrMLM | 8 | 19624757 | 0.0539 | 3.26 | 3.97 | 0.2701 | pLARmEB |
| 2 | 762634 | 0.0153 | 3.48 | 4.21 | 0.1167 | FASTmrMLM | 8 | 26380813 | 0.1099 | 6.03 | 6.86 | 0.3428 | pLARmEB |
| 2 | 3174456 | -0.0347 | 3.37 | 4.08 | 0.519 | FASTmrMLM | 8 | 26504638 | 0.0986 | 4.25 | 5.01 | 0.3373 | pLARmEB |
| 2 | 10989478 | -0.0305 | 5.17 | 5.98 | 0.265 | FASTmrMLM | 8 | 27222965 | -0.0378 | 4.00 | 4.75 | 0.2868 | pLARmEB |
| 2 | 20946677 | -0.023 | 3.56 | 4.29 | 0.1157 | FASTmrMLM | 9 | 5823793 | 0.0453 | 4.18 | 4.94 | 0.2962 | pLARmEB |
| 2 | 21908502 | -0.0205 | 3.22 | 3.93 | 0.2358 | FASTmrMLM | 9 | 8525240 | -0.0172 | 3.03 | 3.72 | 0.151 | pLARmEB |
| 2 | 33725359 | -0.0404 | 4.17 | 4.93 | 0.464 | FASTmrMLM | 9 | 10309830 | 0.0349 | 3.69 | 4.42 | 0.4127 | pLARmEB |
| 2 | 35542100 | -0.0513 | 4.11 | 4.86 | 0.233 | FASTmrMLM | 9 | 21359117 | -0.0455 | 6.44 | 7.28 | 0.7894 | pLARmEB |
| 3 | 5242456 | 0.0864 | 7.04 | 7.90 | 0.6962 | FASTmrMLM | 9 | 21382154 | -0.0453 | 4.22 | 4.99 | 0.4309 | pLARmEB |
| 3 | 12488693 | 0.0351 | 3.46 | 4.19 | 0.2162 | FASTmrMLM | 9 | 21658026 | -0.0335 | 6.52 | 7.37 | 0.5733 | pLARmEB |
| 3 | 16733441 | -0.0322 | 7.06 | 7.93 | 0.5526 | FASTmrMLM | 10 | 9280914 | -0.0497 | 7.87 | 8.76 | 0.5155 | pLARmEB |
| 3 | 25320136 | 0.0246 | 5.54 | 6.36 | 0.3184 | FASTmrMLM | 10 | 19947905 | -0.0651 | 8.55 | 9.46 | 0.6941 | pLARmEB |
| 3 | 35141614 | -0.011 | 3.29 | 4.01 | 0.0571 | FASTmrMLM | 11 | 2557609 | -0.0254 | 4.38 | 5.15 | 0.3144 | pLARmEB |
| 3 | 35268655 | 0.0279 | 4.26 | 5.03 | 0.3424 | FASTmrMLM | 11 | 6920089 | 0.0302 | 4.35 | 5.11 | 0.3416 | pLARmEB |
| 4 | 315092 | 0.024 | 3.32 | 4.04 | 0.1306 | FASTmrMLM | 11 | 8050958 | -0.0486 | 4.43 | 5.20 | 0.7206 | pLARmEB |
| 4 | 4577211 | -0.0285 | 5.71 | 6.53 | 0.4701 | FASTmrMLM | 11 | 19526177 | -0.0451 | 5.37 | 6.18 | 0.5829 | pLARmEB |
| 4 | 19235815 | -0.032 | 4.88 | 5.67 | 0.6049 | FASTmrMLM | 11 | 19643504 | -0.028 | 4.11 | 4.87 | 0.2789 | pLARmEB |
| 4 | 31416786 | 0.019 | 3.46 | 4.18 | 0.1798 | FASTmrMLM | 11 | 21817990 | -0.0544 | 4.36 | 5.13 | 0.3093 | pLARmEB |
| 4 | 31705285 | -0.029 | 5.70 | 6.52 | 0.341 | FASTmrMLM | 11 | 24714951 | -0.0225 | 3.52 | 4.24 | 0.2382 | pLARmEB |
| 5 | 762855 | -0.0289 | 6.24 | 7.08 | 0.2999 | FASTmrMLM | 11 | 28796114 | -0.0197 | 3.05 | 3.75 | 0.1653 | pLARmEB |
| 5 | 913567 | -0.0437 | 4.83 | 5.62 | 0.914 | FASTmrMLM | 12 | 365399 | -0.044 | 4.68 | 5.46 | 0.3382 | pLARmEB |
| 5 | 4859223 | -0.0293 | 4.42 | 5.20 | 0.4699 | FASTmrMLM | 1 | 6491279 | -0.0316 | 3.41 | 4.13 | 3.8122 | pKWmEB |
| 5 | 5376243 | 0.1292 | 31.74 | 32.92 | 5.857 | FASTmrMLM | 2 | 7524480 | 0.0526 | 4.70 | 5.48 | 0.684 | pKWmEB |
| 5 | 14033709 | -0.0426 | 5.72 | 6.54 | 0.7804 | FASTmrMLM | 3 | 5242456 | 0.0797 | 3.60 | 4.33 | 2.4457 | pKWmEB |
| 5 | 23667256 | -0.0309 | 3.68 | 4.41 | 0.2553 | FASTmrMLM | 3 | 35769449 | 0.0299 | 4.11 | 4.87 | 1.1918 | pKWmEB |
| 6 | 1179404 | -0.0202 | 4.56 | 5.33 | 0.2405 | FASTmrMLM | 4 | 13764697 | 0.0482 | 3.84 | 4.59 | 2.2151 | pKWmEB |
| 6 | 27500790 | 0.0606 | 5.38 | 6.19 | 1.4533 | FASTmrMLM | 4 | 22310001 | -0.0435 | 3.28 | 3.99 | 2.2751 | pKWmEB |
| 6 | 29387919 | -0.0574 | 6.14 | 6.97 | 1.1207 | FASTmrMLM | 5 | 5371949 | -0.0662 | 11.63 | 12.59 | 5.2432 | pKWmEB |
| 7 | 19316334 | -0.0276 | 4.33 | 5.10 | 0.4483 | FASTmrMLM | 5 | 5361276 | -0.0477 | 3.74 | 4.48 | 5.0705 | pKWmEB |
| 7 | 21323336 | 0.0257 | 6.96 | 7.82 | 0.3887 | FASTmrMLM | 5 | 5343770 | 0.0827 | 3.45 | 4.17 | 6.6359 | pKWmEB |
| 7 | 23624287 | 0.0361 | 4.91 | 5.70 | 0.4704 | FASTmrMLM | 5 | 5291557 | -0.0747 | 5.09 | 5.89 | 0.2877 | pKWmEB |
| 7 | 27595785 | -0.0363 | 4.14 | 4.90 | 0.2785 | FASTmrMLM | 7 | 28334910 | 0.0454 | 6.25 | 7.09 | 1.503 | pKWmEB |
| 7 | 28321075 | 0.0475 | 5.03 | 5.83 | 0.535 | FASTmrMLM | 7 | 22895807 | 0.0484 | 3.42 | 4.14 | 2.7726 | pKWmEB |
| 7 | 29606768 | 0.0334 | 6.92 | 7.78 | 0.6319 | FASTmrMLM | 8 | 26504638 | 0.1378 | 4.42 | 5.19 | 1.3213 | pKWmEB |
| 8 | 1660498 | 0.04 | 3.63 | 4.36 | 0.9417 | FASTmrMLM | 9 | 21353073 | -0.0818 | 6.74 | 7.60 | 1.9548 | pKWmEB |
| 8 | 2410918 | -0.0218 | 4.59 | 5.37 | 0.2472 | FASTmrMLM | 10 | 19947905 | -0.0707 | 7.00 | 7.87 | 2.5975 | pKWmEB |
| 8 | 3881704 | -0.0258 | 3.25 | 3.96 | 0.2679 | FASTmrMLM | 11 | 27020790 | 0.0279 | 4.25 | 5.02 | 0.9692 | pKWmEB |
| 8 | 4880887 | 0.0179 | 3.49 | 4.21 | 0.1133 | FASTmrMLM | 12 | 21802207 | -0.0324 | 3.28 | 4.00 | 1.5278 | pKWmEB |
| 8 | 7568998 | 0.0453 | 6.31 | 7.15 | 0.5046 | FASTmrMLM | 1 | 3696298 | 0.0579 | 5.27 | 6.07 | 0.8724 | ISIS EM-BLASSO |
| 8 | 17392114 | 0.0417 | 6.16 | 7.00 | 0.7368 | FASTmrMLM | 1 | 6919215 | 0.0367 | 3.40 | 4.12 | 0.5815 | ISIS EM-BLASSO |
| 8 | 26249593 | 0.0471 | 3.42 | 4.14 | 0.1375 | FASTmrMLM | 3 | 3676174 | -0.0916 | 6.64 | 7.49 | 1.0518 | ISIS EM-BLASSO |
| 8 | 26292835 | 0.0573 | 3.80 | 4.54 | 0.8933 | FASTmrMLM | 3 | 5242456 | 0.0871 | 4.65 | 5.43 | 0.7074 | ISIS EM-BLASSO |
| 9 | 5861022 | 0.0537 | 5.72 | 6.54 | 0.7553 | FASTmrMLM | 3 | 25332258 | 0.0247 | 3.88 | 4.62 | 0.3156 | ISIS EM-BLASSO |
| 9 | 7015224 | -0.0398 | 5.39 | 6.20 | 0.5951 | FASTmrMLM | 4 | 8669122 | 0.0876 | 4.71 | 5.50 | 1.201 | ISIS EM-BLASSO |
| 9 | 7648345 | 0.0321 | 5.16 | 5.97 | 0.5894 | FASTmrMLM | 4 | 22310001 | -0.0411 | 3.64 | 4.37 | 0.7156 | ISIS EM-BLASSO |
| 9 | 8525240 | -0.02 | 4.54 | 5.32 | 0.2305 | FASTmrMLM | 4 | 31880075 | -0.0522 | 3.08 | 3.78 | 0.3618 | ISIS EM-BLASSO |
| 9 | 8900138 | -0.028 | 5.93 | 6.77 | 0.4167 | FASTmrMLM | 4 | 32080864 | 0.0341 | 3.19 | 3.90 | 0.6282 | ISIS EM-BLASSO |
| 9 | 10213296 | 0.023 | 4.57 | 5.35 | 0.2864 | FASTmrMLM | 5 | 4831052 | -0.0367 | 5.49 | 6.30 | 0.7348 | ISIS EM-BLASSO |
| 9 | 15024826 | -0.0412 | 4.93 | 5.72 | 0.7395 | FASTmrMLM | 5 | 5186176 | 0.0572 | 3.07 | 3.77 | 1.1965 | ISIS EM-BLASSO |
| 9 | 20251821 | 0.0412 | 5.91 | 6.74 | 0.3481 | FASTmrMLM | 5 | 5300234 | -0.0694 | 4.64 | 5.42 | 0.4589 | ISIS EM-BLASSO |
| 9 | 20559656 | -0.028 | 4.10 | 4.85 | 0.2477 | FASTmrMLM | 5 | 5361276 | -0.0678 | 11.63 | 12.60 | 2.6106 | ISIS EM-BLASSO |
| 9 | 21338733 | -0.0411 | 4.05 | 4.81 | 0.7589 | FASTmrMLM | 5 | 5371949 | -0.0607 | 9.68 | 10.62 | 2.1738 | ISIS EM-BLASSO |
| 9 | 22359906 | -0.029 | 3.65 | 4.38 | 0.4688 | FASTmrMLM | 5 | 5376243 | 0.07 | 7.01 | 7.88 | 1.7167 | ISIS EM-BLASSO |
| 10 | 3943372 | -0.0195 | 3.47 | 4.19 | 0.2111 | FASTmrMLM | 6 | 21315943 | 0.0297 | 3.09 | 3.80 | 0.3887 | ISIS EM-BLASSO |
| 10 | 4649756 | -0.0451 | 4.61 | 5.39 | 0.5017 | FASTmrMLM | 6 | 27500790 | 0.0499 | 3.74 | 4.48 | 0.9871 | ISIS EM-BLASSO |
| 10 | 19451104 | -0.0482 | 5.42 | 6.24 | 1.0079 | FASTmrMLM | 6 | 29387919 | -0.0442 | 3.13 | 3.83 | 0.6646 | ISIS EM-BLASSO |
| 10 | 19947905 | -0.0769 | 10.37 | 11.32 | 1.0915 | FASTmrMLM | 7 | 16169282 | -0.0308 | 4.29 | 5.05 | 0.4787 | ISIS EM-BLASSO |
| 11 | 693197 | -0.0271 | 3.65 | 4.38 | 0.3327 | FASTmrMLM | 7 | 21285184 | -0.051 | 3.96 | 4.71 | 1.0014 | ISIS EM-BLASSO |
| 11 | 7058901 | 0.0338 | 6.82 | 7.68 | 0.5245 | FASTmrMLM | 7 | 21666972 | 0.0375 | 5.20 | 6.01 | 0.8275 | ISIS EM-BLASSO |
| 11 | 8050958 | -0.0441 | 4.63 | 5.41 | 0.6697 | FASTmrMLM | 7 | 23951267 | -0.0477 | 3.74 | 4.48 | 0.6759 | ISIS EM-BLASSO |
| 11 | 8857734 | 0.0244 | 3.87 | 4.62 | 0.1139 | FASTmrMLM | 7 | 28321075 | 0.0493 | 5.81 | 6.63 | 0.5757 | ISIS EM-BLASSO |
| 11 | 10933886 | -0.0376 | 9.62 | 10.55 | 0.8381 | FASTmrMLM | 8 | 2410918 | -0.0218 | 3.06 | 3.76 | 0.2477 | ISIS EM-BLASSO |
| 11 | 19456239 | -0.0433 | 7.38 | 8.25 | 0.7129 | FASTmrMLM | 8 | 26504638 | 0.113 | 4.63 | 5.41 | 0.4993 | ISIS EM-BLASSO |
| 11 | 19643504 | -0.034 | 7.22 | 8.09 | 0.4641 | FASTmrMLM | 9 | 21393671 | -0.0579 | 4.87 | 5.66 | 1.5211 | ISIS EM-BLASSO |
| 11 | 21815738 | 0.0322 | 3.10 | 3.81 | 0.3844 | FASTmrMLM | 10 | 19946757 | -0.0609 | 6.19 | 7.03 | 0.6668 | ISIS EM-BLASSO |
| 11 | 23360520 | 0.036 | 3.30 | 4.01 | 0.4149 | FASTmrMLM | 11 | 6920089 | 0.0373 | 5.86 | 6.69 | 0.5874 | ISIS EM-BLASSO |
| 12 | 9405542 | 0.0397 | 3.78 | 4.52 | 0.4726 | FASTmrMLM | 11 | 11034491 | -0.0282 | 3.35 | 4.07 | 0.2883 | ISIS EM-BLASSO |
| 12 | 18270626 | -0.0318 | 3.59 | 4.32 | 0.4891 | FASTmrMLM | 12 | 365399 | -0.0431 | 3.73 | 4.47 | 0.365 | ISIS EM-BLASSO |
| 12 | 19906987 | 0.0279 | 3.55 | 4.28 | 0.4592 | FASTmrMLM | 12 | 21802207 | -0.0364 | 3.69 | 4.42 | 0.4837 | ISIS EM-BLASSO |
| 12 | 21802207 | -0.0434 | 7.89 | 8.78 | 0.6892 | FASTmrMLM |  |  |  |  |  |  |  |
