# Supplementary material for: mrMLM v4.0.2: An R Platform for Multi-locus Genome-wide Association Studies
Source: Genomics Proteomics Bioinformatics. 2020 Dec 18;18(4):481–7. doi: 10.1016/j.gpb.2020.06.006 (PMC8242264; doi:10.1016/j.gpb.2020.06.006)
Supplement: Supplementary Table S3 — Previously reported genes for grain width in rice around the QTNs identified by our multi-locus GWAS methods [file mmc13.docx]

**Table S3 Previously reported genes for grain width in rice around the QTNs identified by our multi-locus GWAS methods**

| Chr | Marker  position (nt) | Multi-locus GWAS | | | | Comparative genomics analysis | | | |
| --- | --- | --- | --- | --- | --- | --- | --- | --- | --- |
|  |  | **QTN effect** | **LOD score** | **r^2^ (%)** | **Method** | **RAP locus** | **Gene** | **Functional annotation** | **PMID** |
| 1 | 23986872 | -0.0237 | 4.15 | 0.28 | 1 | Os01g0625900 | *OsOFP2* | seed size, grain width | 26706069 |
| 2 | 7524480 | 0.0526 | 4.70 | 0.68 | 5 | Os02g0234200 | *FUWA* | grain shape, grain width | 26043067 |
| 3 | 3676174 | -0.0916~-0.0705 | 5.25~6.64 | 0.55~1.05 | 4, 6 | Os03g0175800 | *BG1* | grain size, grain width | 26283354 |
| 4 | 31880075 | -0.0522 | 3.08 | 0.36 | 6 | Os04g0645100 | *flo2* | grain size, grain width | 20889913 |
| 5 | 762855 | -0.0453~-0.0289 | 4.03~6.24 | 0.30~0.74 | 1, 2 | Os05g0115800 | *OsMKP1* | grain size, grain width | 29588389 |
| 5 | 3627700 | 0.0376 | 3.95 | 0.63 | 1 | Os05g0158500 | *GS5* | grain size, grain width | 25711711 |
| 5 | **5361276** | **-0.1108~-0.0477** | **3.29~11.63** | **1.29~5.07** | **3, 4, 5, 6** | **Os05g0187500** | ***GW5*** | **grain size, grain width** | **28394310** |
| 6 | 1179404 | -0.0524~-0.0202 | 4.56~5.90 | 0.24~0.44 | 1, 2, 3 | Os06g0130400 | *OsACS6* | grain size, grain width | 26792122 |
| 7 | 22895807 | 0.0484 | 3.42 | 2.77 | 5 | Os07g0580500 | *OsBZR1* | seed size, grain width | 25754973 |
| 7 | 23951267 | -0.0477 | 3.74 | 0.68 | 6 | Os07g0603300 | *qGL7* | grain shape, grain width | 20184774 |
| 8 | 26019239 | -0.0414 | 3.58 | 0.59 | 1 | Os08g0537800 | *WTG1* | grain size, grain width | 28621888 |
| 8 | 27222965 | -0.0378 | 4.00 | 0.29 | 4 | Os08g0562500 | *SLG* | grain width | 27252468 |
| 9 | **21338733** | **-0.0411** | **4.05** | **0.76** | **2** | **Os09g0540800** | ***OsFD1*** | **transcription factors** | **16293693** |
| 2 | 20311039 | -0.0175 | 4.03 | 0.15 | 4 | Os02g0554000 | *SDG725* | grain size | 22136623 |
| 2 | 24640538 | 0.0269 | 3.45 | 0.29 | 1 | Os02g0614100 | *OsNST1* | grain size | 21383162 |
| 2 | 33721702 | -0.0385 | 4.76 | 0.37 | 1 | Os02g0787300 | *OsMKK4* | grain size | 24320692 |
| 3 | 5242456 | 0.0778~0.0871 | 3.60~7.04 | 0.50~2.45 | 2, 4, 5, 6 | Os03g0215400 | *OsMADS1* | grain shape, grain size | 29487282 |
| 3 | 6865287 | 0.0251 | 5.98 | 0.29 | 4 | Os03g0236900 | *OsAPC6* | grain size | 20091079 |
| 3 | 7230027 | -0.0679~-0.0442 | 4.21~4.69 | 0.50~0.89 | 1, 3 | Os03g0254400 | *OspPLAIIIα* | grain size, seed size | 26290597 |
| 3 | 16733441 | -0.0603~-0.0281 | 5.014~12.00 | 0.37~1.20 | 1, 2, 3, 4 | Os03g0407400 | *GS3* | grain size, grain width | 16453132 |
| 3 | 35141614 | -0.0110 | 3.30 | 0.06 | 2 | Os03g0837300 | *OsNaPRT1* | grain size | 21805338 |
| 8 | 1660498 | 0.0400~0.0526 | 3.05~3.63 | 0.94~1.63 | 1, 2 | Os08g0137100 | *OsFIE2* | grain size | 27764161 |
| 11 | 19643504 | -0.0340~-0.0280 | 4.11~7.22 | 0.28~0.46 | 2, 4 | Os11g0540500 | *IKU2* | grain width (Arabidopsis) | 16293693 |

*Note:* Method 1, 2, 3, 4, 5, and 6 represent mrMLM, FASTmrMLM, FASTmrEMMA, pLARmEB, pKWmEB, and ISIS EM-BLASSO, respectively. The genes with bold type were also detected in a previous study [28]. Twenty-two genes were found at http://www.ricedata.cn/, and one gene (*IKU2*) from *Arabidopsis* was obtained from [28] (PMID: 16293693). QTN, quantitative trait nucleotide; LOD, log of odds; QTN, QTN. RAP, Rice Annotation Project.
