# Supplementary material for: mrMLM v4.0.2: An R Platform for Multi-locus Genome-wide Association Studies
Source: Genomics Proteomics Bioinformatics. 2020 Dec 18;18(4):481–7. doi: 10.1016/j.gpb.2020.06.006 (PMC8242264; doi:10.1016/j.gpb.2020.06.006)
Supplement: Supplementary Table S4 — Fifteen new candidate genes for rice grain size and development detected by our multi-locus GWAS methods [file mmc14.docx]

**Table S4 Fifteen new candidate genes for rice grain size and development detected by our multi-locus GWAS methods**

| Chr | Position (bp) | Multi-locus genome-wide association studies | | | | KEGG analysis | | | | | Gene expressional analysis | |
| --- | --- | --- | --- | --- | --- | --- | --- | --- | --- | --- | --- | --- |
|  |  | **QTN effect** | **LOD** | **r^2^ (%)** | **Method** | **ID** | ***P* value** | **Functional annotation** | **PMID** | **Candidate gene** | **Nipponbare** | **Average expressional level** |
| 2 | **762634** | **0.02** | **3.48** | **0.12** | **2** | **hsa04141** | **0.0153** | **Protein processing in endoplasmic reticulum** | **15659439** | **Os02g0115900** | **696.23, 187.01, 421.91, 748.59** | **12.26, 11.93, 10.60** |
| 5 | **4859223** | **-0.05~-0.03** | **4.42~8.18** | **0.47~0.78** | **2, 4** | **P00060** | **0.019** | **Ubiquitin proteasome pathway** | **17417637** | **Os05g0182500** | **105.84, 61.56, 28.82, 45.0** | **10.51, 8.53, 8.63** |
| 1 | 41275767 | -0.03 | 7.59 | 0.56 | 2 | hsa00100 | 0.0005 | Steroid biosynthesis | 22438020 | Os01g0940000 | 0, 0.30, 0, 0 | 3.99, 4.78, 3.26 |
| 4 | 4577211 | -0.03 | 5.71 | 0.47 | 2 | hsa00980 | 4.69E-06 | Metabolism of xenobiotics by cytochrome P450 | 24082112 | Os04g0167800 | 0, 0, 0, 0 | 3.80, 2.78, 3.01 |
| 4 | 5115500 | 0.06 | 5.16 | 0.81 | 4 | hsa00980 | 4.69E-06 | Metabolism of xenobiotics by cytochrome P450 | 24082112 | Os04g0174100 | 0, 0, 0, 0 |  |
| 4 | 22310001 | -0.04~-0.04 | 3.28~3.64 | 0.72~2.28 | 5, 6 | hsa00600 | 0.0217 | Sphingolipid metabolism | 27454201 | Os04g0447400 | 0.49, 0.58, 0, 0.15 | 3.90, 2.70, 3.09 |
| 5 | 4833479 | -0.07 | 4.25 | 0.58 | 3 | hsa00562 | 0.0479 | Inositol phosphate metabolism | 17961936 | Os05g0180600 | 6.05, 9.70, 5.28, 4.97 | 5.96, 4.23, 4.27 |
| 5 | 5291557 | -0.07 | 5.09 | 0.29 | 5 | hsa00520 | 0.0231 | Amino sugar and nucleotide sugar metabolism. | 21383162 | Os05g0187100 | 11.42, 3.29, 0.27, 0.66 | 8.65, 6.72, 6.89 |
| 5 | 5656974 | -0.09 | 4.5 | 0.47 | 1 | hsa04120 | 0.0479 | Ubiquitin mediated proteolysis | 17417637 | Os05g0193900 | 0.79, 1.16, 0, 0 |  |
| 8 | 7568998 | 0.05 | 6.31 | 0.50 | 2 | P00060 | 0.019 | Ubiquitin proteasome pathway | 17417637 | Os08g0224700 | 41.33, 29.70, 16.74, 22.93 | 9.39, 7.93, 7.98 |
| 9 | 4595714 | -0.07 | 4.29 | 0.57 | 3 | hsa00100 | 0.0005 | Steroid biosynthesis | 22438020 | Os09g0262000 | 0, 0, 0, 0 | 3.24, 2.79, 2.77 |
| 9 | 5823793 | 0.05 | 4.18 | 0.30 | 4 | hsa00562 | 0.0479 | Inositol phosphate metabolism | 17961936 | Os09g0278300 | 0.86, 2.23, 0.52, 1.27 |  |
| 9 | 7015224 | -0.06~-0.04 | 5.39~7.65 | 0.60~1.49 | 1, 2 | hsa04120 | 0.0479 | Ubiquitin mediated proteolysis | 17417637 | Os09g0294300 | 2.81, 0.34, 0.94, 0.76 | 6.39, 3.62, 2.98 |
| 9 | 20251821 | 0.04 | 5.91 | 0.35 | 2 | hsa00562 | 0.0479 | Inositol phosphate metabolism | 17961936 | Os09g0518700 | 20.25, 51.81, 4.73, 9.16 | 9.37, 8.76, 9.28 |
| 10 | 9280914 | -0.08~-0.05 | 4.7 | 0.45~0.52 | 3, 4 | hsa00600 | 0.02167 | Sphingolipid metabolism | 27454201 | Os10g0330600 | 0, 0, 0, 0 | 3.29, 2.71, 2.68 |

*Note:* The six methods mrMLM, FASTmrMLM, FASTmrEMMA, pLARmEB, pKWmEB, and ISIS EM-BLASSO were marked by numbers 1 to 6, respectively. The numbers in the Nipponbare column were gene expression levels at four tissues and stages (seed-5 days after pollination (DAP); embryo-25 DAP; endosperm-25 DAP; seed-10 DAP), in which their average expression levels were 28.40, 26.73, 43.20 and 33.30 (FPKM), respectively ([http://rice.plantbiology.msu.edu/index.shtml](http://rice.plantbiology.msu.edu/index.shtml.)). The numbers in the column of average expressional level were average expressional levels of each gene at three stages (7, 14 and 21 DAP) between Minghui 63 and Zhenshan 97, and the average expression levels for all the genes were 5.63 (7 DAP), 4.76 (14 DAP) and 4.92 (21 DAP) (<https://www.ncbi.nlm.nih.gov>/geo/query/acc.cgi?acc=GSE19024). The two candidate genes with bold type are the most likely candidate genes.
