# Supplementary material for: mrMLM v4.0.2: An R Platform for Multi-locus Genome-wide Association Studies
Source: Genomics Proteomics Bioinformatics. 2020 Dec 18;18(4):481–7. doi: 10.1016/j.gpb.2020.06.006 (PMC8242264; doi:10.1016/j.gpb.2020.06.006)
Supplement: Supplementary Table S5 — All the QTNs for oil concentration in maize detected by our multi-locus GWAS methods [file mmc15.docx]

**Table S5 All the QTNs for oil concentration in maize detected by our multi-locus GWAS methods**

| Chr | Position (bp) | QTN effect | LOD score | –Log_10_ *P* | r^2^ (%) | Method | Chr | Position (bp) | QTN effect | LOD score | –Log_10_ *P* | r^2^ (%) | Method |
| --- | --- | --- | --- | --- | --- | --- | --- | --- | --- | --- | --- | --- | --- |
| 1 | 52762430 | -0.326 | 6.08 | 6.9185 | 0.593 | mrMLM | 10 | 113390827 | 0.055 | 4.14 | 4.9022 | 0.0987 | FASTmrMLM |
| 1 | 198416421 | 0.1638 | 7.73 | 8.6171 | 1.026 | mrMLM | 1 | 61716135 | 0.1268 | 3.21 | 3.9156 | 0.1484 | FASTmrEMMA |
| 1 | 296768029 | -0.197 | 4.10 | 4.8600 | 0.4604 | mrMLM | 2 | 24388940 | -0.2221 | 4.54 | 5.3196 | 0.4615 | FASTmrEMMA |
| 1 | 13536744 | -0.1207 | 4.56 | 5.3426 | 0.4347 | mrMLM | 2 | 53680157 | 0.4481 | 7.49 | 8.3641 | 0.8778 | FASTmrEMMA |
| 1 | 35740220 | 0.093 | 3.99 | 4.7383 | 0.3329 | mrMLM | 2 | 150020854 | 0.2362 | 3.16 | 3.8606 | 0.347 | FASTmrEMMA |
| 1 | 245479447 | -0.0731 | 3.90 | 4.6413 | 0.181 | mrMLM | 2 | 189219763 | 0.1956 | 3.78 | 4.5169 | 0.3031 | FASTmrEMMA |
| 1 | 292354425 | 0.1129 | 5.38 | 6.1910 | 0.3631 | mrMLM | 3 | 217876667 | 0.3502 | 8.07 | 8.9675 | 0.796 | FASTmrEMMA |
| 1 | 104610891 | -0.1088 | 6.41 | 7.2514 | 0.4119 | mrMLM | 4 | 5012443 | -0.2349 | 5.91 | 6.7352 | 0.5382 | FASTmrEMMA |
| 1 | 14847409 | -0.1221 | 5.18 | 5.9781 | 0.5568 | mrMLM | 4 | 6601755 | -0.4893 | 6.04 | 6.8765 | 0.8407 | FASTmrEMMA |
| 2 | 53680401 | -0.1785 | 5.96 | 6.7865 | 0.5254 | mrMLM | 4 | 47531927 | 0.2101 | 5.18 | 5.9800 | 0.393 | FASTmrEMMA |
| 2 | 24390872 | -0.1288 | 7.28 | 8.1531 | 0.6334 | mrMLM | 4 | 231411384 | -0.4372 | 6.24 | 7.0816 | 0.9882 | FASTmrEMMA |
| 2 | 183922016 | -0.0778 | 3.17 | 3.8711 | 0.2057 | mrMLM | 5 | 4769561 | -0.2764 | 8.17 | 9.0620 | 0.6749 | FASTmrEMMA |
| 2 | 150704340 | 0.073 | 3.61 | 4.3425 | 0.207 | mrMLM | 5 | 15186727 | -0.6589 | 11.60 | 12.5647 | 1.6394 | FASTmrEMMA |
| 2 | 193735059 | 0.1495 | 4.31 | 5.0750 | 0.599 | mrMLM | 5 | 25619033 | 0.2995 | 3.22 | 3.9292 | 0.3995 | FASTmrEMMA |
| 2 | 211393932 | 0.1247 | 9.75 | 10.6872 | 0.6045 | mrMLM | 5 | 40921864 | 0.3075 | 5.51 | 6.3254 | 0.3488 | FASTmrEMMA |
| 3 | 220509353 | -0.2918 | 17.22 | 18.2725 | 1.8379 | mrMLM | 5 | 150074309 | 0.2264 | 3.46 | 4.1852 | 0.4655 | FASTmrEMMA |
| 3 | 222753455 | 0.1035 | 4.59 | 5.3713 | 0.3783 | mrMLM | 5 | 179939948 | -0.2669 | 6.62 | 7.4685 | 0.5573 | FASTmrEMMA |
| 3 | 181564906 | -0.0915 | 3.79 | 4.5341 | 0.2992 | mrMLM | 6 | 97113705 | 0.355 | 8.22 | 9.1142 | 0.8974 | FASTmrEMMA |
| 3 | 29473154 | -0.1277 | 4.55 | 5.3279 | 0.4028 | mrMLM | 6 | 104865718 | -0.8046 | 18.64 | 19.7117 | 3.4443 | FASTmrEMMA |
| 3 | 222482863 | -0.1351 | 5.96 | 6.7957 | 0.5602 | mrMLM | 6 | 163018858 | -0.2897 | 4.59 | 5.3642 | 0.6121 | FASTmrEMMA |
| 4 | 216687458 | 0.1526 | 8.89 | 9.8035 | 0.6063 | mrMLM | 7 | 9198079 | -0.3048 | 8.09 | 8.9820 | 0.6776 | FASTmrEMMA |
| 5 | 188656939 | -0.1897 | 4.40 | 5.1658 | 0.6172 | mrMLM | 8 | 22170626 | -0.586 | 7.22 | 8.0886 | 0.9214 | FASTmrEMMA |
| 5 | 150074309 | 0.1039 | 4.60 | 5.3763 | 0.42 | mrMLM | 8 | 157465327 | -0.3783 | 13.62 | 14.6237 | 1.3356 | FASTmrEMMA |
| 5 | 51435319 | 0.1353 | 8.97 | 9.8811 | 0.6889 | mrMLM | 8 | 169138120 | -0.2304 | 4.50 | 5.2707 | 0.378 | FASTmrEMMA |
| 5 | 1362747 | -0.1493 | 9.35 | 10.2759 | 0.6602 | mrMLM | 8 | 171597499 | 0.1769 | 4.32 | 5.0838 | 0.295 | FASTmrEMMA |
| 5 | 193656423 | 0.1007 | 5.00 | 5.7928 | 0.3686 | mrMLM | 8 | 173031488 | 0.3357 | 7.58 | 8.4586 | 1.1015 | FASTmrEMMA |
| 6 | 104858202 | -0.2489 | 8.25 | 9.1473 | 1.1242 | mrMLM | 9 | 15614702 | -0.2808 | 7.49 | 8.3653 | 0.7429 | FASTmrEMMA |
| 7 | 173251838 | -0.8954 | 37.84 | 39.0638 | 5.132 | mrMLM | 9 | 153562715 | 0.3369 | 10.41 | 11.3599 | 1.0341 | FASTmrEMMA |
| 7 | 8846936 | 0.0863 | 6.54 | 7.3859 | 0.2828 | mrMLM | 10 | 24578257 | -0.4537 | 8.11 | 9.0032 | 0.9837 | FASTmrEMMA |
| 7 | 6146507 | -0.1329 | 6.03 | 6.8640 | 0.4554 | mrMLM | 10 | 26483664 | -0.5464 | 11.95 | 12.9252 | 1.7625 | FASTmrEMMA |
| 7 | 155392927 | 0.1477 | 9.08 | 9.9989 | 0.6005 | mrMLM | 10 | 102370515 | -0.2628 | 5.67 | 6.4864 | 0.5725 | FASTmrEMMA |
| 7 | 133531802 | -0.108 | 4.19 | 4.9516 | 0.2551 | mrMLM | 10 | 117214050 | -0.2643 | 5.41 | 6.2252 | 0.582 | FASTmrEMMA |
| 7 | 92391957 | -0.1302 | 6.56 | 7.4081 | 0.4095 | mrMLM | 1 | 7966534 | -0.3313 | 7.48 | 8.3550 | 0.0533 | pLARmEB |
| 8 | 125298375 | -0.4993 | 20.45 | 21.5345 | 1.494 | mrMLM | 1 | 8657361 | 0.1048 | 4.85 | 5.6438 | 0.036 | pLARmEB |
| 8 | 75605439 | -0.151 | 5.66 | 6.4783 | 0.3037 | mrMLM | 1 | 16349283 | -0.2684 | 6.26 | 7.1012 | 0.0791 | pLARmEB |
| 8 | 29146171 | -0.1522 | 7.14 | 8.0074 | 0.6325 | mrMLM | 1 | 17668962 | -0.2078 | 3.71 | 4.4454 | 0.0371 | pLARmEB |
| 8 | 111625183 | -0.1496 | 7.04 | 7.9051 | 0.6626 | mrMLM | 1 | 55071145 | -0.1667 | 3.98 | 4.7293 | 0.0379 | pLARmEB |
| 8 | 138882711 | -0.1112 | 8.15 | 9.0495 | 0.468 | mrMLM | 2 | 24390872 | -0.1069 | 5.44 | 6.2539 | 0.0446 | pLARmEB |
| 9 | 153836357 | -0.1498 | 11.89 | 12.8598 | 0.8055 | mrMLM | 2 | 149341223 | -0.4782 | 8.58 | 9.4854 | 0.0914 | pLARmEB |
| 9 | 153562715 | 0.1084 | 4.20 | 4.9619 | 0.4488 | mrMLM | 2 | 149517374 | -0.1826 | 4.42 | 5.1932 | 0.0515 | pLARmEB |
| 10 | 102319748 | -0.1384 | 8.57 | 9.4812 | 0.5323 | mrMLM | 2 | 212526845 | -0.87 | 12.53 | 13.5159 | 0.3677 | pLARmEB |
| 10 | 88614957 | -0.1147 | 5.11 | 5.9147 | 0.2557 | mrMLM | 3 | 171029218 | 0.1341 | 3.35 | 4.0672 | 0.0252 | pLARmEB |
| 1 | 162267119 | 0.0751 | 5.06 | 5.8547 | 0.1646 | FASTmrMLM | 3 | 202363278 | -0.5809 | 6.08 | 6.9167 | 0.2068 | pLARmEB |
| 1 | 171060219 | -0.1986 | 6.01 | 6.8460 | 0.316 | FASTmrMLM | 3 | 222596913 | 0.0668 | 3.34 | 4.0579 | 0.0111 | pLARmEB |
| 1 | 198416421 | 0.0982 | 8.33 | 9.2319 | 0.3687 | FASTmrMLM | 4 | 185387136 | -0.1498 | 3.73 | 4.4691 | 0.0453 | pLARmEB |
| 1 | 231186353 | 0.0603 | 4.33 | 5.0947 | 0.1376 | FASTmrMLM | 5 | 4769561 | -0.1229 | 6.73 | 7.5841 | 0.0546 | pLARmEB |
| 1 | 248149904 | 0.2619 | 7.78 | 8.6693 | 0.5496 | FASTmrMLM | 5 | 25615959 | 0.1039 | 3.50 | 4.2260 | 0.0208 | pLARmEB |
| 1 | 293269574 | -0.0795 | 4.37 | 5.1400 | 0.1526 | FASTmrMLM | 6 | 163012904 | -0.1388 | 8.99 | 9.9031 | 0.0657 | pLARmEB |
| 2 | 2551778 | -0.2423 | 6.43 | 7.2737 | 0.3518 | FASTmrMLM | 7 | 17889236 | 0.1112 | 4.13 | 4.8836 | 0.0493 | pLARmEB |
| 2 | 19283564 | -0.0675 | 4.46 | 5.2335 | 0.1532 | FASTmrMLM | 7 | 109329336 | -0.2769 | 6.11 | 6.9516 | 0.0782 | pLARmEB |
| 2 | 149517635 | -0.3293 | 14.17 | 15.1817 | 0.9115 | FASTmrMLM | 7 | 141513616 | 0.1239 | 4.89 | 5.6773 | 0.0388 | pLARmEB |
| 2 | 172555792 | -0.0807 | 5.93 | 6.7645 | 0.2504 | FASTmrMLM | 8 | 38489776 | -0.2909 | 3.45 | 4.1757 | 0.0829 | pLARmEB |
| 2 | 183922016 | -0.1204 | 11.09 | 12.0471 | 0.4927 | FASTmrMLM | 8 | 100960678 | -0.5827 | 12.32 | 13.3057 | 0.2782 | pLARmEB |
| 2 | 193735059 | 0.1194 | 5.77 | 6.5930 | 0.3819 | FASTmrMLM | 8 | 142177238 | -0.4392 | 8.99 | 9.9023 | 0.0603 | pLARmEB |
| 2 | 212636843 | 0.0783 | 6.60 | 7.4532 | 0.2347 | FASTmrMLM | 8 | 157463010 | 0.1253 | 6.46 | 7.3045 | 0.0401 | pLARmEB |
| 2 | 212744513 | -0.0918 | 5.70 | 6.5183 | 0.2988 | FASTmrMLM | 8 | 169138120 | -0.1091 | 3.81 | 4.5512 | 0.0347 | pLARmEB |
| 3 | 150169183 | -0.0874 | 8.99 | 9.9018 | 0.248 | FASTmrMLM | 9 | 10320276 | -0.475 | 10.36 | 11.3056 | 0.0902 | pLARmEB |
| 3 | 217633424 | 0.0802 | 4.59 | 5.3655 | 0.2513 | FASTmrMLM | 9 | 15614693 | -0.0602 | 3.68 | 4.4146 | 0.014 | pLARmEB |
| 3 | 221689683 | 0.0881 | 8.10 | 8.9990 | 0.2173 | FASTmrMLM | 9 | 17648206 | -0.1434 | 4.77 | 5.5576 | 0.0409 | pLARmEB |
| 4 | 62868426 | -0.1606 | 5.01 | 5.8115 | 0.227 | FASTmrMLM | 9 | 26601762 | 0.1785 | 8.59 | 9.4979 | 0.0633 | pLARmEB |
| 5 | 4769561 | -0.0726 | 5.26 | 6.0706 | 0.1863 | FASTmrMLM | 9 | 93146938 | 0.1198 | 5.40 | 6.2102 | 0.0415 | pLARmEB |
| 5 | 30995288 | -0.0523 | 3.98 | 4.7340 | 0.104 | FASTmrMLM | 1 | 248149904 | 0.2631 | 3.89 | 4.6399 | 1.5504 | pKWmEB |
| 5 | 171274922 | 0.2473 | 4.69 | 5.4779 | 0.1078 | FASTmrMLM | 1 | 268158349 | -0.1328 | 4.01 | 4.7611 | 0.322 | pKWmEB |
| 5 | 196508497 | 0.0678 | 3.21 | 3.9190 | 0.0923 | FASTmrMLM | 1 | 292354425 | 0.1453 | 4.59 | 5.3731 | 0.9693 | pKWmEB |
| 6 | 104865718 | -0.2528 | 18.25 | 19.3195 | 1.3604 | FASTmrMLM | 1 | 162267119 | 0.152 | 5.32 | 6.1251 | 0.717 | pKWmEB |
| 7 | 5138788 | -0.1107 | 6.10 | 6.9316 | 0.4547 | FASTmrMLM | 2 | 149517374 | -0.1973 | 3.47 | 4.1975 | 0.9209 | pKWmEB |
| 7 | 17889236 | 0.0967 | 3.91 | 4.6575 | 0.365 | FASTmrMLM | 2 | 2805975 | 0.116 | 3.23 | 3.9420 | 0.955 | pKWmEB |
| 7 | 141513616 | 0.0675 | 3.23 | 3.9423 | 0.1125 | FASTmrMLM | 4 | 5306611 | 0.2592 | 7.14 | 8.0042 | 0.887 | pKWmEB |
| 7 | 173251838 | -0.4825 | 13.80 | 14.8026 | 1.49 | FASTmrMLM | 5 | 4769561 | -0.1628 | 7.90 | 8.7899 | 0.9587 | pKWmEB |
| 8 | 21818669 | -0.1363 | 4.10 | 4.8576 | 0.2132 | FASTmrMLM | 5 | 75890500 | -0.1166 | 4.50 | 5.2793 | 0.6919 | pKWmEB |
| 8 | 26347033 | -0.2665 | 10.95 | 11.9060 | 0.5973 | FASTmrMLM | 6 | 104865718 | -0.1752 | 3.74 | 4.4825 | 1.7929 | pKWmEB |
| 8 | 38412621 | -0.2182 | 8.90 | 9.8175 | 0.6345 | FASTmrMLM | 8 | 141649337 | 0.2186 | 9.09 | 10.0127 | 2.388 | pKWmEB |
| 8 | 118681100 | 0.1413 | 6.00 | 6.8291 | 0.2292 | FASTmrMLM | 8 | 26346491 | -0.4393 | 5.05 | 5.8529 | 2.938 | pKWmEB |
| 8 | 125317148 | 0.0892 | 3.85 | 4.5919 | 0.0823 | FASTmrMLM | 8 | 21818669 | -0.1941 | 3.08 | 3.7769 | 1.5137 | pKWmEB |
| 8 | 148482834 | 0.2478 | 10.03 | 10.9702 | 0.3678 | FASTmrMLM | 8 | 160374255 | -0.2746 | 7.28 | 8.1484 | 0.8709 | pKWmEB |
| 8 | 158369778 | 0.1012 | 9.49 | 10.4222 | 0.3971 | FASTmrMLM | 10 | 23787517 | -0.8471 | 12.79 | 13.7840 | 8.9586 | pKWmEB |
| 8 | 160374255 | -0.3099 | 18.86 | 19.9356 | 0.9202 | FASTmrMLM | 10 | 16487724 | -0.2464 | 3.41 | 4.1351 | 1.2385 | pKWmEB |
| 9 | 3477249 | 0.0691 | 4.70 | 5.4858 | 0.1436 | FASTmrMLM | 10 | 141946713 | -0.2304 | 3.71 | 4.4495 | 0.9887 | pKWmEB |
| 9 | 15614702 | -1.00E-04 | 3.02 | 3.7152 | 4.12E-07 | FASTmrMLM | 6 | 102199028 | -0.4623 | 4.82 | 5.6067 | 1.455 | ISIS EM-BLASSO |
| 9 | 133900817 | -0.0518 | 3.35 | 4.0666 | 0.0909 | FASTmrMLM | 6 | 104865718 | -0.3231 | 7.51 | 8.3865 | 2.2225 | ISIS EM-BLASSO |
| 9 | 153836357 | -0.0364 | 3.68 | 4.4130 | 0.0475 | FASTmrMLM | 7 | 109329336 | -0.3232 | 3.64 | 4.3757 | 1.0412 | ISIS EM-BLASSO |
| 10 | 23787517 | -0.6508 | 21.92 | 23.0190 | 2.7114 | FASTmrMLM | 8 | 26346491 | -0.859 | 14.16 | 15.1713 | 5.3208 | ISIS EM-BLASSO |
| 10 | 45433753 | 0.1734 | 9.21 | 10.1282 | 0.4541 | FASTmrMLM | 8 | 100960678 | -0.5971 | 4.86 | 5.6475 | 2.8563 | ISIS EM-BLASSO |
| 10 | 102366558 | -0.0553 | 3.93 | 4.6768 | 0.0898 | FASTmrMLM | 10 | 23787517 | -0.91 | 8.79 | 9.7053 | 5.3004 | ISIS EM-BLASSO |
