# Supplementary material for: mrMLM v4.0.2: An R Platform for Multi-locus Genome-wide Association Studies
Source: Genomics Proteomics Bioinformatics. 2020 Dec 18;18(4):481–7. doi: 10.1016/j.gpb.2020.06.006 (PMC8242264; doi:10.1016/j.gpb.2020.06.006)
Supplement: Supplementary Table S6 — Comparison of seed oil related genes in maize identified by the software mrMLM in this study with those in the ref [29]. [file mmc16.docx]

**Table S6 Comparison of seed oil related genes in maize identified by the software mrMLM in this study with those in the ref [29]**

| Chr | QTN position (bp) | GWAS | | | |  | Comparative genomics analysis | | | |
| --- | --- | --- | --- | --- | --- | --- | --- | --- | --- | --- |
|  |  | **Effect** | **LOD or *P* value** | **r^2^ (%)** | **Method ^†^** |  | **Locus name** | **Gene and its functional annotation** | **Distance (kb) ^‡^** | **PMID** |
| QTNs detected simultaneously by the software mrMLM and Li et al. (2013) Nat Genet 45(1):43-50. | | | | | | | | | | |
| 1 | 16349283 | -0.27 | 6.26 | 0.08 | 4 |  | *GRMZM2G080524* | Epoxide hydrolase, *EH* | 21 | 22303198, 23505340 |
| 1 | 248149904 | 0.26 | 7.78 | 0.55 | 2 |  | *GRMZM2G110298* | Acyl carrier protein, *ACP* | 0 | 3383847, 24186730, 15279774 |
| 2 | 53680401 | -0.18 | 5.96 | 0.53 | 3 |  | *GRMZM2G134432* | Phosphatidylinosito 3 kinase, *PI3Ks.a* | 678 | 3383847, 12177484 |
| 2 | 149341223~149517635 | -0.48~-0.18 | 4.42~14.17 | 0.05~0.91 | 2,4 |  | *GRMZM2G079236* | Long-chain Acyl-CoA synthetase, *LACS* | 0 | 3383847, 12366803, 17934760 |
| 4 | 6601755 | -0.49 | 6.04 | 0.84 | 3 |  | *GRMZM2G099666* | Acyl-coenzyme A oxidase, *ACX2* | 193 |  |
| 6 | 104858202~104865718 | -0.80~-0.32 | 3.74~18.25 | 1.12~3.44 | 1~3,5,6 |  | *GRMZM2G169089* | Diglyceride acyltransferase, *DGAT1-2* | 0 | 3383847, 21245192, 18631243, 9835636 |
| 7 | 109329336 | -0.32~-0.28 | 3.64~6.11 | 0.08~1.04 | 4,6 |  | *GRMZM2G092550* | Phosphatidylinositol 3 kinase, *PI3Ks.b* | 4 | 3383847, 12177484 |
| 8 | 38412621~38489776 | -0.29~-0.22 | 3.45~8.90 | 0.08~0.63 | 2,4 |  | *GRMZM2G003022* | COPII-coated vesicles, *COPII* | 31 | 3383847, 12677402 |
| 10 | 16487724 | -0.25 | 3.41 | 1.23 | 5 |  | *GRMZM2G169240* | Fatty acid desaturase-1, *FAD2* | 2582 | 3383847, 21245192, 7907506, 11297732, 9500987 |
| 10 | 117214050 | -0.2643 | 5.41 | 0.58 | 3 |  | *GRMZM5G828253* | Oxidoreductase activity, cytochrome P450, *CYPOR* | 313 | 8378325, 19619160 |
| QTNs detected only by the software mrMLM, candidate genes were identified by key words like: fatty acid, oil and triacylglycerol biosynthesis in the MaizeGDB (https://www.maizegdb.org/). | | | | | | | | | | |
| 1 | 13536744 | -0.12 | 4.56 | 0.43 | 1 |  | *GRMZM2G031790* | 3-ketoacyl-CoA synthase 2, *KCS2* | 589 | 28510842 |
| 1 | 296768029 | -0.20 | 4.10 | 0.46 | 1 |  | *GRMZM2G369815* | Seed fatty acid reducer 4, *SFAR4* | 609 | 30967882 |
| 2 | 211393932 | 0.12 | 9.75 | 0.60 | 1 |  | *GRMZM2G124335* | Fatty acid biosynthesis 1, *FAB1* | 2 | 18978071 |
| 2 | 24388940 | -0.22 | 4.54 | 0.46 | 3 |  | *GRMZM2G078373* | Sphingolipid delta4-desaturase, *DES-1-LIKE* | 840 | 28642782 |
| 4 | 216687458 | 0.15 | 8.89 | 0.61 | 1 |  | *GRMZM2G091715* | Acyl carrier protein 4, *ACP4* | 19 | 30704481 |
| 5 | 1362747 | -0.15 | 9.35 | 0.66 | 1 |  | *GRMZM2G019866* | Acyl carrier protein 1, *ACP1* | 548 | 16226259 |
| 6 | 97113705 | 0.36 | 8.22 | 0.90 | 3 |  | *GRMZM2G322892* | Triacylglycerol lipase 2, *LIP2* | 268 | 12671095 |
| 6 | 102199028 | -0.46 | 4.82 | 1.46 | 6 |  | *GRMZM2G079308* | Fatty acyl-ACP thioesterases B, *FATB* | 950 | 31001857 |
| 7 | 8846936 | 0.09 | 6.54 | 0.28 | 1 |  | *GRMZM2G174766* | Fatty acid desaturase 2, *FAD2* | 928 | 16449229 |
| 7 | 141513616 | 0.07~0.12 | 3.23~4.89 | 0.04~0.11 | 2,4 |  | *GRMZM2G020740* | 3-ketoacyl-CoA synthase 4, *KCS4* | 502 | 22023480 |
| 7 | 92391957 | -0.13 | 6.56 | 0.41 | 1 |  | *GRMZM2G129453* | Delta(8)-fatty-acid desaturase 2, *SLD2* | 549 | 25893869 |
| 9 | 10320276 | -0.48 | 10.36 | 0.09 | 4 |  | *GRMZM2G012863* | 3-ketoacyl-acyl carrier protein synthase Ⅰ, *KASI* | 897 | 10521705 |
| 9 | 17648206 | -0.14 | 4.77 | 0.04 | 4 |  | *GRMZM5G864319* | Peroxisomal acyl-coenzyme A oxidase 1, *ACX1* | 327 |  |
| QTNs detected only by Li et al. (2013) Nat Genet 45(1):43-50. | | | | | | | | | | |
| 3 | 166664152 |  | 4.6e-07 |  |  |  | *GRMZM2G176542* | Triglyceride lipases, *TAGL* | 0 | 3383847, 24309818, 9370311 |
| 3 | 167431166 |  | 1.1e-06 |  |  |  | *GRMZM2G118423* | Oxidoreductase activity, Cytochrome P450, *CYPOR* | 45 | 8378325, 19619160 |
| 3 | 178136002 |  | 8.5e-07 |  |  |  | *GRMZM2G083195* | Glycerol-phosphate acyltransferase, *GPAT* | 46 | 3383847, 20551224, 14684887, 25950803 |
| 4 | 32810884 |  | 9.4e-07 |  |  |  | *GRMZM5G847159* | Oxidoreductase activity, cytochrome P450, *CYPOR* | 1 | 8378325, 19619160 |

*Note*: ^†^, 1, 2, 3, 4, 5, and 6 represent mrMLM, FASTmrMLM, FASTmrEMMA, pLARmEB, pKWmEB, and ISIS EM-BLASSO, respectively. ^‡^, distance (kb) between QTNs and gene.
