# Supplementary material for: mrMLM v4.0.2: An R Platform for Multi-locus Genome-wide Association Studies
Source: Genomics Proteomics Bioinformatics. 2020 Dec 18;18(4):481–7. doi: 10.1016/j.gpb.2020.06.006 (PMC8242264; doi:10.1016/j.gpb.2020.06.006)
Supplement: Supplementary Table S7 — All the QTNs for kidney weight in Simmental beef cattle detected by our multi-locus GWAS methods [file mmc17.docx]

**Table S7 All the QTNs for kidney weight in Simmental beef cattle detected by our multi-locus GWAS methods**

| Chr | Position (bp) | QTN effect | LOD score | –Log_10_ *P* | r^2^ (%) | Method | Chr | Position (bp) | QTN effect | LOD score | –L og_10_ *P* | r^2^ (%) | Method |
| --- | --- | --- | --- | --- | --- | --- | --- | --- | --- | --- | --- | --- | --- |
| 10 | 87848389 | -0.0539 | 7.03 | 7.9 | 6.5537 | mrMLM | 23 | 21702537 | -0.0034 | 14.1 | 15.11 | 0.0022 | FASTmrEMMA |
| 11 | 33921550 | 0.0305 | 3.76 | 4.5 | 2.3121 | mrMLM | 23 | 24692423 | -0.0051 | 4.02 | 4.77 | 0.0085 | FASTmrEMMA |
| 19 | 26569324 | 0.0367 | 3.12 | 3.82 | 2.9988 | mrMLM | 24 | 2010941 | -0.0025 | 3.24 | 3.95 | 0.0011 | FASTmrEMMA |
| 26 | 36522514 | 0.0395 | 3.27 | 3.99 | 3.8983 | mrMLM | 24 | 9740339 | 8.00E-04 | 3.19 | 3.9 | 2.00E-04 | FASTmrEMMA |
| 1 | 106358966 | 0.019 | 4.76 | 5.55 | 0.8435 | FASTmrMLM | 24 | 25697647 | -7.00E-04 | 3.12 | 3.82 | 1.69E-05 | FASTmrEMMA |
| 1 | 110649323 | -0.0268 | 5.79 | 6.61 | 1.6839 | FASTmrMLM | 24 | 25768771 | -7.00E-04 | 3.14 | 3.84 | 1.52E-05 | FASTmrEMMA |
| 2 | 2254298 | 0.0187 | 5.96 | 6.8 | 0.8176 | FASTmrMLM | 24 | 25951960 | -3.00E-04 | 4.16 | 4.92 | 1.62E-06 | FASTmrEMMA |
| 2 | 18246330 | 0.0107 | 3.12 | 3.83 | 0.2469 | FASTmrMLM | 24 | 58552772 | 9.00E-04 | 13.31 | 14.31 | 1.80E-05 | FASTmrEMMA |
| 3 | 115147732 | 0.0204 | 8.2 | 9.1 | 0.436 | FASTmrMLM | 25 | 7598966 | 0.0078 | 4.75 | 5.54 | 0.0211 | FASTmrEMMA |
| 4 | 2835171 | -0.0131 | 7.15 | 8.02 | 0.3461 | FASTmrMLM | 26 | 21564772 | 0.0099 | 18.28 | 19.35 | 0.0293 | FASTmrEMMA |
| 4 | 43362940 | 0.0221 | 5.99 | 6.82 | 0.9088 | FASTmrMLM | 26 | 27826727 | -0.0024 | 4.71 | 5.5 | 5.00E-04 | FASTmrEMMA |
| 4 | 61093205 | 0.0139 | 3.8 | 4.54 | 0.2639 | FASTmrMLM | 27 | 3852629 | 0.0011 | 6.67 | 7.52 | 4.00E-04 | FASTmrEMMA |
| 4 | 111413395 | 0.0155 | 4.71 | 5.49 | 0.5003 | FASTmrMLM | 27 | 3853699 | 0.0014 | 8.75 | 9.66 | 7.00E-04 | FASTmrEMMA |
| 4 | 120615269 | -0.0201 | 3.98 | 4.73 | 0.3849 | FASTmrMLM | 27 | 14236927 | -8.00E-04 | 8.83 | 9.74 | 1.86E-05 | FASTmrEMMA |
| 5 | 13463428 | 0.0029 | 6.14 | 6.98 | 0.0163 | FASTmrMLM | 27 | 16625147 | -0.0019 | 5.31 | 6.12 | 6.00E-04 | FASTmrEMMA |
| 5 | 19436069 | -0.0123 | 3.56 | 4.29 | 0.3455 | FASTmrMLM | 27 | 16832909 | -0.0012 | 3.36 | 4.08 | 1.00E-04 | FASTmrEMMA |
| 5 | 120338298 | -0.0094 | 3.09 | 3.79 | 0.1952 | FASTmrMLM | 27 | 16834042 | -0.0015 | 6.47 | 7.32 | 3.00E-04 | FASTmrEMMA |
| 6 | 21123441 | -0.032 | 6.6 | 7.45 | 1.159 | FASTmrMLM | 27 | 18546318 | -0.005 | 5.26 | 6.07 | 0.0092 | FASTmrEMMA |
| 6 | 25471760 | 0.0105 | 5.08 | 5.88 | 0.1698 | FASTmrMLM | 27 | 22739277 | -0.0024 | 3.02 | 3.71 | 0.002 | FASTmrEMMA |
| 6 | 42110373 | 0.0067 | 3.65 | 4.39 | 0.0349 | FASTmrMLM | 27 | 22757505 | -0.0035 | 4.23 | 5 | 0.0041 | FASTmrEMMA |
| 7 | 20296966 | -0.0225 | 4.77 | 5.55 | 0.8617 | FASTmrMLM | 27 | 38287694 | 0.003 | 12.16 | 13.14 | 0.0021 | FASTmrEMMA |
| 7 | 72721038 | -0.022 | 3.98 | 4.73 | 1.1011 | FASTmrMLM | 27 | 42890396 | -0.0024 | 4.43 | 5.2 | 8.00E-04 | FASTmrEMMA |
| 8 | 27592207 | -0.0165 | 4.83 | 5.62 | 0.553 | FASTmrMLM | 27 | 43733075 | 0.0019 | 7.44 | 8.32 | 0.001 | FASTmrEMMA |
| 8 | 107120944 | 0.0258 | 3.82 | 4.56 | 1.0643 | FASTmrMLM | 28 | 3532094 | -0.0029 | 5.1 | 5.9 | 0.001 | FASTmrEMMA |
| 10 | 5526827 | 0.0195 | 4.35 | 5.12 | 0.6004 | FASTmrMLM | 29 | 17566806 | 0.0062 | 5.67 | 6.49 | 0.0129 | FASTmrEMMA |
| 10 | 21317870 | -0.0336 | 5.84 | 6.66 | 1.5648 | FASTmrMLM | 29 | 49511085 | 0.0151 | 4.33 | 5.1 | 0.0772 | FASTmrEMMA |
| 10 | 87848389 | -0.0373 | 10 | 10.94 | 2.9108 | FASTmrMLM | 1 | 9427932 | 0.0088 | 5.72 | 6.54 | 0.1315 | pLARmEB |
| 10 | 90978450 | -0.0184 | 8.59 | 9.5 | 0.7477 | FASTmrMLM | 1 | 34164923 | -0.0085 | 3.84 | 4.59 | 0.1793 | pLARmEB |
| 11 | 50973309 | -0.0016 | 3.87 | 4.62 | 0.0052 | FASTmrMLM | 1 | 65752195 | 0.001 | 4.98 | 5.77 | 0.0014 | pLARmEB |
| 11 | 66048270 | 0.003 | 3.38 | 4.1 | 0.0189 | FASTmrMLM | 1 | 120987043 | 0.0032 | 9.00 | 9.91 | 0.0206 | pLARmEB |
| 11 | 91627517 | 0.0186 | 3.25 | 3.96 | 0.2778 | FASTmrMLM | 1 | 133085461 | 0.0036 | 4.53 | 5.30 | 0.0339 | pLARmEB |
| 12 | 30043755 | 4.00E-04 | 4.13 | 4.89 | 4.00E-04 | FASTmrMLM | 1 | 155322206 | -0.0034 | 8.02 | 8.92 | 0.0312 | pLARmEB |
| 12 | 59135006 | -0.0303 | 5.01 | 5.8 | 1.8107 | FASTmrMLM | 2 | 11903885 | 0.0051 | 13.03 | 14.03 | 0.0703 | pLARmEB |
| 12 | 84524504 | 0.0253 | 10.2 | 11.14 | 1.4713 | FASTmrMLM | 2 | 20169565 | 0.0011 | 6.37 | 7.21 | 7.00E-04 | pLARmEB |
| 13 | 42320076 | -0.0243 | 6.4 | 7.25 | 1.2289 | FASTmrMLM | 2 | 52417495 | -7.00E-04 | 15.21 | 16.24 | 2.00E-04 | pLARmEB |
| 13 | 60570356 | 0.0257 | 4.47 | 5.24 | 1.4059 | FASTmrMLM | 3 | 25794439 | -2.04E-05 | 4.34 | 5.11 | 1.34E-07 | pLARmEB |
| 14 | 10829335 | 0.0137 | 3.02 | 3.71 | 0.2039 | FASTmrMLM | 3 | 35135984 | 0.0113 | 6.37 | 7.22 | 0.322 | pLARmEB |
| 14 | 21917782 | 0.0079 | 3.29 | 4.01 | 0.0856 | FASTmrMLM | 3 | 37282673 | 0.0028 | 9.11 | 10.03 | 0.0188 | pLARmEB |
| 15 | 8576846 | 0.0233 | 4.6 | 5.38 | 1.2459 | FASTmrMLM | 3 | 51935982 | 6.00E-04 | 6.07 | 6.90 | 5.00E-04 | pLARmEB |
| 15 | 68472538 | -5.00E-04 | 3.85 | 4.6 | 7.00E-04 | FASTmrMLM | 3 | 55957304 | 0.0021 | 4.91 | 5.71 | 0.0029 | pLARmEB |
| 16 | 14813526 | -0.0271 | 4.09 | 4.85 | 1.7082 | FASTmrMLM | 3 | 57416268 | -0.0024 | 6.19 | 7.03 | 0.007 | pLARmEB |
| 17 | 32625001 | 0.0329 | 5.17 | 5.98 | 2.2679 | FASTmrMLM | 3 | 93556351 | -0.0037 | 7.99 | 8.88 | 0.0356 | pLARmEB |
| 17 | 51676255 | 0.0187 | 5.79 | 6.62 | 0.6449 | FASTmrMLM | 3 | 120403408 | 0.005 | 8.97 | 9.89 | 0.0592 | pLARmEB |
| 18 | 38317858 | 0.0269 | 5.64 | 6.46 | 1.6866 | FASTmrMLM | 4 | 2845004 | -0.0052 | 8.42 | 9.33 | 0.0707 | pLARmEB |
| 18 | 54604727 | -0.0027 | 3.3 | 4.02 | 0.0151 | FASTmrMLM | 4 | 5006344 | 6.00E-04 | 7.06 | 7.93 | 3.00E-04 | pLARmEB |
| 19 | 60859466 | -0.0131 | 5.24 | 6.04 | 0.4042 | FASTmrMLM | 4 | 26662279 | -0.0011 | 13.27 | 14.26 | 0.0015 | pLARmEB |
| 20 | 44864240 | 0.0288 | 9.71 | 10.64 | 1.9296 | FASTmrMLM | 4 | 30681746 | 0.004 | 10.39 | 11.34 | 0.0257 | pLARmEB |
| 22 | 21951237 | -0.0139 | 5.09 | 5.89 | 0.3223 | FASTmrMLM | 4 | 47490095 | -9.00E-04 | 3.27 | 3.98 | 9.00E-04 | pLARmEB |
| 23 | 14902969 | -0.0241 | 4.95 | 5.74 | 1.3311 | FASTmrMLM | 4 | 50267564 | 0.0043 | 15.45 | 16.48 | 0.017 | pLARmEB |
| 23 | 21336942 | 0.0564 | 3.23 | 3.93 | 2.2647 | FASTmrMLM | 4 | 55527318 | -2.00E-04 | 39.35 | 40.57 | 1.25E-05 | pLARmEB |
| 24 | 45436667 | -0.0076 | 4.61 | 5.39 | 0.1349 | FASTmrMLM | 4 | 89075410 | 0.0037 | 21.17 | 22.27 | 0.0282 | pLARmEB |
| 26 | 21564772 | 0.0231 | 5.2 | 6 | 1.2334 | FASTmrMLM | 4 | 111413395 | 0.0087 | 7.20 | 8.07 | 0.1873 | pLARmEB |
| 26 | 26369697 | -0.0201 | 8.78 | 9.7 | 0.5686 | FASTmrMLM | 4 | 118912501 | -7.00E-04 | 13.98 | 14.99 | 1.00E-04 | pLARmEB |
| 27 | 792244 | 0.0099 | 3.54 | 4.26 | 0.1449 | FASTmrMLM | 5 | 15061346 | 0.0066 | 12.50 | 13.49 | 0.0665 | pLARmEB |
| 27 | 8476228 | 0.0101 | 3.36 | 4.08 | 0.2049 | FASTmrMLM | 5 | 15116972 | 0.005 | 14.83 | 15.86 | 0.037 | pLARmEB |
| 27 | 39546339 | 0.0132 | 4.65 | 5.43 | 0.3908 | FASTmrMLM | 5 | 23903481 | 0.0043 | 7.19 | 8.06 | 0.0454 | pLARmEB |
| 28 | 34780664 | 0.0224 | 5.56 | 6.38 | 1.1451 | FASTmrMLM | 5 | 38190841 | -0.0023 | 18.36 | 19.43 | 0.0146 | pLARmEB |
| 28 | 36504079 | 9.04E-05 | 3.52 | 4.25 | 1.37E-05 | FASTmrMLM | 5 | 47042381 | 4.00E-04 | 4.53 | 5.31 | 3.00E-04 | pLARmEB |
| 29 | 43539616 | 0.0193 | 5.25 | 6.05 | 0.8177 | FASTmrMLM | 5 | 48121153 | 0.0063 | 5.37 | 6.18 | 0.1051 | pLARmEB |
| 1 | 12953453 | -0.0023 | 7.6 | 8.48 | 0.0012 | FASTmrEMMA | 5 | 67286371 | -0.0011 | 10.12 | 11.06 | 4.00E-04 | pLARmEB |
| 1 | 27730401 | 8.00E-04 | 3.29 | 4.01 | 1.47E-05 | FASTmrEMMA | 5 | 68166725 | 0.0031 | 7.18 | 8.05 | 0.0013 | pLARmEB |
| 1 | 110520102 | 0.0013 | 3.33 | 4.05 | 4.00E-04 | FASTmrEMMA | 5 | 104413139 | 2.00E-04 | 8.07 | 8.97 | 1.00E-04 | pLARmEB |
| 1 | 133085461 | 0.006 | 6.2 | 7.04 | 0.0078 | FASTmrEMMA | 5 | 118127379 | 0.0013 | 6.26 | 7.10 | 0.0035 | pLARmEB |
| 1 | 133086487 | 0.0053 | 5.22 | 6.02 | 0.0062 | FASTmrEMMA | 5 | 118397277 | 4.00E-04 | 10.62 | 11.57 | 3.00E-04 | pLARmEB |
| 1 | 138305770 | -0.0014 | 7.19 | 8.06 | 1.00E-04 | FASTmrEMMA | 6 | 21123441 | -0.0064 | 9.49 | 10.42 | 0.0543 | pLARmEB |
| 1 | 141823000 | 5.00E-04 | 9.31 | 10.23 | 4.07E-06 | FASTmrEMMA | 6 | 39387542 | 0.0026 | 12.00 | 12.98 | 0.0057 | pLARmEB |
| 1 | 141826766 | 5.00E-04 | 9.58 | 10.51 | 5.70E-06 | FASTmrEMMA | 6 | 39410541 | 0.005 | 25.96 | 27.10 | 0.0426 | pLARmEB |
| 1 | 148538451 | -5.00E-04 | 6.1 | 6.93 | 5.71E-06 | FASTmrEMMA | 6 | 44967444 | 0.0012 | 14.16 | 15.17 | 0.0039 | pLARmEB |
| 1 | 150311512 | -0.0032 | 3.44 | 4.16 | 0.0028 | FASTmrEMMA | 6 | 72068201 | -0.0015 | 4.25 | 5.02 | 0.0049 | pLARmEB |
| 1 | 150317595 | -0.0029 | 4.11 | 4.87 | 0.0023 | FASTmrEMMA | 7 | 28834853 | -9.00E-04 | 5.89 | 6.72 | 9.00E-04 | pLARmEB |
| 1 | 155312956 | -4.00E-04 | 10.42 | 11.37 | 2.30E-06 | FASTmrEMMA | 7 | 53107671 | 0.0032 | 7.77 | 8.65 | 0.028 | pLARmEB |
| 2 | 18246330 | 0.01 | 8.3 | 9.19 | 0.0333 | FASTmrEMMA | 7 | 71033098 | -0.003 | 8.24 | 9.14 | 0.0185 | pLARmEB |
| 2 | 46723266 | -0.001 | 5.77 | 6.6 | 2.00E-04 | FASTmrEMMA | 7 | 83599948 | -0.001 | 17.26 | 18.31 | 3.00E-04 | pLARmEB |
| 2 | 54235770 | 0.0415 | 11.71 | 12.68 | 0.407 | FASTmrEMMA | 7 | 89744240 | -0.0018 | 3.02 | 3.72 | 0.0032 | pLARmEB |
| 2 | 81187013 | -3.00E-04 | 3 | 3.7 | 9.00E-07 | FASTmrEMMA | 7 | 98954416 | 8.00E-04 | 19.50 | 20.58 | 8.00E-04 | pLARmEB |
| 2 | 85037178 | 0.004 | 6.2 | 7.04 | 0.0046 | FASTmrEMMA | 8 | 27592207 | -0.0106 | 3.80 | 4.54 | 0.2705 | pLARmEB |
| 2 | 136271704 | 0.0028 | 5.3 | 6.11 | 0.0011 | FASTmrEMMA | 8 | 38000683 | -0.0014 | 25.25 | 26.39 | 0.0022 | pLARmEB |
| 2 | 136535374 | 0.0022 | 4.81 | 5.6 | 7.00E-04 | FASTmrEMMA | 9 | 41431307 | 0.0022 | 7.01 | 7.87 | 0.0061 | pLARmEB |
| 3 | 2245496 | -0.001 | 3.14 | 3.85 | 1.94E-05 | FASTmrEMMA | 9 | 82856321 | -0.0018 | 16.51 | 17.55 | 0.0079 | pLARmEB |
| 3 | 22281672 | -0.0043 | 3.98 | 4.73 | 0.0056 | FASTmrEMMA | 10 | 10622667 | 0.0039 | 15.35 | 16.38 | 0.0214 | pLARmEB |
| 3 | 32247139 | -8.00E-04 | 10.52 | 11.47 | 6.49E-05 | FASTmrEMMA | 10 | 21317870 | -0.0093 | 4.74 | 5.52 | 0.1408 | pLARmEB |
| 3 | 32260522 | -0.0117 | 5.07 | 5.86 | 0.0472 | FASTmrEMMA | 10 | 87848389 | -0.0165 | 11.05 | 12.01 | 0.6724 | pLARmEB |
| 3 | 35135984 | 0.0042 | 4.27 | 5.04 | 0.0038 | FASTmrEMMA | 10 | 94890549 | 0.0016 | 6.54 | 7.39 | 9.00E-04 | pLARmEB |
| 3 | 35570341 | 0.004 | 10.39 | 11.34 | 0.0034 | FASTmrEMMA | 11 | 105397592 | 0.0047 | 8.18 | 9.07 | 0.0274 | pLARmEB |
| 3 | 35635939 | -0.001 | 13.5 | 14.5 | 2.10E-05 | FASTmrEMMA | 12 | 8001704 | 0.0011 | 22.41 | 23.52 | 0.0032 | pLARmEB |
| 3 | 60755786 | -0.0015 | 6.18 | 7.02 | 2.00E-04 | FASTmrEMMA | 12 | 45303004 | -0.0051 | 6.63 | 7.48 | 0.0482 | pLARmEB |
| 3 | 80430626 | -0.0047 | 7.2 | 8.07 | 0.0067 | FASTmrEMMA | 12 | 52455037 | -7.00E-04 | 8.34 | 9.24 | 2.00E-04 | pLARmEB |
| 3 | 90262349 | 0.0013 | 4.6 | 5.38 | 2.00E-04 | FASTmrEMMA | 12 | 56168000 | -8.00E-04 | 9.08 | 10.00 | 2.00E-04 | pLARmEB |
| 3 | 93556351 | -0.0016 | 5.33 | 6.13 | 9.00E-04 | FASTmrEMMA | 12 | 59135006 | -0.0075 | 4.83 | 5.62 | 0.1318 | pLARmEB |
| 3 | 101850519 | 5.00E-04 | 3.34 | 4.06 | 5.02E-06 | FASTmrEMMA | 12 | 81211856 | 0.0011 | 4.56 | 5.34 | 0.0014 | pLARmEB |
| 3 | 115147732 | 0.0067 | 8.16 | 9.06 | 0.0143 | FASTmrEMMA | 13 | 10102104 | 0.0068 | 16.85 | 17.89 | 0.1196 | pLARmEB |
| 4 | 5589375 | -4.00E-04 | 3.24 | 3.95 | 2.11E-06 | FASTmrEMMA | 13 | 39533788 | 0.0022 | 5.06 | 5.86 | 0.0136 | pLARmEB |
| 4 | 5767201 | 0.0036 | 3.8 | 4.54 | 0.0021 | FASTmrEMMA | 13 | 43482413 | -7.00E-04 | 6.34 | 7.18 | 1.00E-04 | pLARmEB |
| 4 | 11587232 | 0.0038 | 6.17 | 7.01 | 0.002 | FASTmrEMMA | 13 | 68196929 | 0.0026 | 3.78 | 4.52 | 0.0185 | pLARmEB |
| 4 | 23580709 | -0.0089 | 6.08 | 6.91 | 0.0296 | FASTmrEMMA | 14 | 7154835 | 0.0019 | 4.41 | 5.18 | 0.005 | pLARmEB |
| 4 | 31233839 | -0.0013 | 3.43 | 4.15 | 4.00E-04 | FASTmrEMMA | 14 | 59442036 | 0.0013 | 12.47 | 13.45 | 7.00E-04 | pLARmEB |
| 4 | 31790534 | -0.0062 | 10.89 | 11.84 | 0.0101 | FASTmrEMMA | 14 | 80350162 | 0.0073 | 8.46 | 9.37 | 0.1345 | pLARmEB |
| 4 | 72633007 | 5.00E-04 | 3.58 | 4.31 | 3.38E-06 | FASTmrEMMA | 15 | 8835116 | -0.0016 | 4.66 | 5.44 | 0.0016 | pLARmEB |
| 4 | 89058238 | 0.0055 | 9.03 | 9.94 | 0.0101 | FASTmrEMMA | 15 | 32268220 | -0.001 | 5.31 | 6.12 | 7.00E-04 | pLARmEB |
| 4 | 89075410 | 0.0061 | 7.99 | 8.89 | 0.0132 | FASTmrEMMA | 15 | 40521052 | -0.0015 | 10.09 | 11.03 | 0.0038 | pLARmEB |
| 4 | 104775809 | -0.0012 | 5.97 | 6.81 | 8.05E-05 | FASTmrEMMA | 15 | 48963568 | 9.00E-04 | 7.34 | 8.22 | 8.00E-04 | pLARmEB |
| 4 | 106194302 | -0.0019 | 5.19 | 5.99 | 3.00E-04 | FASTmrEMMA | 16 | 1595222 | -0.0025 | 6.04 | 6.87 | 0.0062 | pLARmEB |
| 4 | 111413395 | 0.0056 | 4.09 | 4.84 | 0.0102 | FASTmrEMMA | 16 | 4452514 | -0.0043 | 9.01 | 9.93 | 0.0298 | pLARmEB |
| 4 | 118912501 | -6.00E-04 | 4.08 | 4.84 | 7.46E-06 | FASTmrEMMA | 16 | 14813526 | -0.0102 | 16.91 | 17.96 | 0.2855 | pLARmEB |
| 5 | 1559287 | 6.00E-04 | 9.66 | 10.59 | 9.33E-05 | FASTmrEMMA | 16 | 17770213 | -9.00E-04 | 10.24 | 11.18 | 4.00E-04 | pLARmEB |
| 5 | 13075314 | -0.0012 | 3.59 | 4.32 | 5.00E-04 | FASTmrEMMA | 16 | 23268022 | 0.0012 | 4.16 | 4.92 | 2.00E-04 | pLARmEB |
| 5 | 38001237 | -0.0028 | 5.01 | 5.81 | 0.0027 | FASTmrEMMA | 16 | 59383104 | 2.00E-04 | 4.24 | 5.00 | 2.68E-05 | pLARmEB |
| 5 | 88048251 | 7.00E-04 | 7.5 | 8.38 | 2.00E-04 | FASTmrEMMA | 16 | 60496060 | -7.00E-04 | 3.23 | 3.94 | 4.00E-04 | pLARmEB |
| 5 | 88054351 | 3.00E-04 | 6.47 | 7.32 | 2.48E-05 | FASTmrEMMA | 16 | 60942210 | 0.0068 | 10.17 | 11.11 | 0.1089 | pLARmEB |
| 5 | 112293257 | 2.00E-04 | 4.13 | 4.89 | 6.92E-06 | FASTmrEMMA | 16 | 69766481 | -0.0023 | 3.08 | 3.78 | 0.014 | pLARmEB |
| 5 | 118399856 | 6.00E-04 | 8.75 | 9.66 | 2.02E-05 | FASTmrEMMA | 16 | 72291455 | 0.0023 | 3.41 | 4.13 | 0.009 | pLARmEB |
| 6 | 21105453 | -0.0054 | 8.1 | 8.99 | 0.0083 | FASTmrEMMA | 17 | 32625001 | 0.0058 | 25.36 | 26.49 | 0.0825 | pLARmEB |
| 6 | 39410541 | 0.0031 | 14.92 | 15.94 | 0.0011 | FASTmrEMMA | 17 | 32661304 | 0.0022 | 9.99 | 10.93 | 0.0129 | pLARmEB |
| 6 | 71870305 | -0.0011 | 5.35 | 6.16 | 4.35E-05 | FASTmrEMMA | 17 | 69892739 | 8.00E-04 | 7.67 | 8.55 | 2.00E-04 | pLARmEB |
| 6 | 72076018 | -0.0027 | 4.24 | 5 | 0.0012 | FASTmrEMMA | 18 | 13016845 | 4.00E-04 | 3.82 | 4.56 | 1.00E-04 | pLARmEB |
| 6 | 72841953 | 0.0025 | 10.19 | 11.13 | 8.00E-04 | FASTmrEMMA | 18 | 25754843 | -0.0107 | 3.62 | 4.35 | 0.2923 | pLARmEB |
| 6 | 100620519 | -8.00E-04 | 9.32 | 10.25 | 1.54E-05 | FASTmrEMMA | 18 | 38317858 | 0.0089 | 35.11 | 36.32 | 0.2164 | pLARmEB |
| 6 | 100626375 | -9.00E-04 | 5.56 | 6.38 | 1.75E-05 | FASTmrEMMA | 18 | 39961400 | 0.0051 | 4.23 | 5.00 | 0.0534 | pLARmEB |
| 6 | 113157379 | -0.0036 | 3.1 | 3.81 | 0.0034 | FASTmrEMMA | 19 | 33155236 | 7.00E-04 | 16.55 | 17.59 | 1.00E-04 | pLARmEB |
| 7 | 30916696 | -9.00E-04 | 8.8 | 9.72 | 7.45E-05 | FASTmrEMMA | 19 | 52265051 | 0.003 | 5.65 | 6.47 | 0.0217 | pLARmEB |
| 7 | 36236133 | -0.001 | 3.38 | 4.1 | 3.00E-04 | FASTmrEMMA | 19 | 57708681 | -8.48E-05 | 4.58 | 5.36 | 1.84E-06 | pLARmEB |
| 7 | 36268647 | -0.0014 | 3.77 | 4.51 | 6.00E-04 | FASTmrEMMA | 20 | 10328240 | -5.00E-04 | 10.75 | 11.70 | 5.30E-05 | pLARmEB |
| 7 | 53124827 | 0.0021 | 12.75 | 13.73 | 0.0011 | FASTmrEMMA | 20 | 24932795 | -0.0015 | 4.64 | 5.42 | 0.0047 | pLARmEB |
| 7 | 65539778 | 3.00E-04 | 6.12 | 6.96 | 1.12E-06 | FASTmrEMMA | 20 | 37391207 | -0.0013 | 3.94 | 4.68 | 0.003 | pLARmEB |
| 7 | 70532694 | -8.00E-04 | 3.85 | 4.59 | 1.16E-05 | FASTmrEMMA | 20 | 71511933 | -0.006 | 3.52 | 4.25 | 0.0542 | pLARmEB |
| 7 | 70779399 | -5.00E-04 | 3.41 | 4.13 | 8.15E-06 | FASTmrEMMA | 21 | 5370856 | -0.0061 | 8.32 | 9.22 | 0.0876 | pLARmEB |
| 7 | 70816154 | -5.00E-04 | 4.81 | 5.6 | 7.13E-06 | FASTmrEMMA | 21 | 27386386 | -0.0013 | 3.98 | 4.73 | 5.00E-04 | pLARmEB |
| 8 | 8522529 | 0.0011 | 3.4 | 4.11 | 2.00E-04 | FASTmrEMMA | 21 | 31277031 | 0.0028 | 15.41 | 16.44 | 0.0205 | pLARmEB |
| 8 | 38200914 | 0.0027 | 3.8 | 4.55 | 0.0011 | FASTmrEMMA | 21 | 45767354 | -0.0038 | 4.63 | 5.41 | 0.0242 | pLARmEB |
| 8 | 38210062 | 0.0024 | 5.94 | 6.78 | 8.00E-04 | FASTmrEMMA | 21 | 69841951 | 5.00E-04 | 9.18 | 10.11 | 6.20E-05 | pLARmEB |
| 8 | 40422559 | -0.0015 | 5 | 5.79 | 7.00E-04 | FASTmrEMMA | 22 | 43700547 | 0.0013 | 14.64 | 15.66 | 0.0046 | pLARmEB |
| 8 | 40425857 | 4.00E-04 | 5.47 | 6.28 | 4.33E-05 | FASTmrEMMA | 22 | 56907006 | 0.0059 | 20.97 | 22.07 | 0.0464 | pLARmEB |
| 8 | 54150455 | -5.00E-04 | 4.02 | 4.77 | 6.41E-06 | FASTmrEMMA | 22 | 57693714 | 0.001 | 6.61 | 7.46 | 0.001 | pLARmEB |
| 8 | 98559973 | 8.00E-04 | 8.34 | 9.24 | 2.13E-05 | FASTmrEMMA | 22 | 59128692 | 0.0047 | 4.29 | 5.06 | 0.0299 | pLARmEB |
| 9 | 26042975 | -0.0014 | 3.32 | 4.04 | 3.00E-04 | FASTmrEMMA | 24 | 25951960 | -4.00E-04 | 8.21 | 9.11 | 3.54E-05 | pLARmEB |
| 9 | 32079319 | -6.00E-04 | 8.67 | 9.58 | 1.17E-05 | FASTmrEMMA | 24 | 27838377 | -0.0073 | 10.64 | 11.59 | 0.111 | pLARmEB |
| 9 | 85391712 | 0.0034 | 3.42 | 4.14 | 0.0037 | FASTmrEMMA | 24 | 34515639 | 0.0045 | 3.24 | 3.95 | 0.0068 | pLARmEB |
| 9 | 98048267 | 0.0048 | 7.33 | 8.2 | 0.006 | FASTmrEMMA | 26 | 27651115 | -0.001 | 3.07 | 3.77 | 0.0026 | pLARmEB |
| 10 | 55918765 | 0.0013 | 3.16 | 3.87 | 6.00E-04 | FASTmrEMMA | 27 | 16835142 | -0.0031 | 9.50 | 10.43 | 0.0124 | pLARmEB |
| 10 | 56798705 | -6.00E-04 | 6.84 | 7.7 | 7.02E-06 | FASTmrEMMA | 27 | 22604390 | -0.005 | 5.97 | 6.80 | 0.067 | pLARmEB |
| 10 | 57724034 | 0.035 | 4.21 | 4.98 | 0.2061 | FASTmrEMMA | 27 | 37937057 | -0.0033 | 4.11 | 4.87 | 0.0243 | pLARmEB |
| 10 | 94890549 | 8.00E-04 | 4.81 | 5.6 | 1.30E-05 | FASTmrEMMA | 27 | 42241046 | 0.0021 | 11.29 | 12.25 | 0.0113 | pLARmEB |
| 10 | 102677040 | 0.016 | 3.93 | 4.68 | 0.0629 | FASTmrEMMA | 29 | 49713592 | -0.0058 | 3.65 | 4.38 | 0.0923 | pLARmEB |
| 11 | 48806854 | -0.0074 | 6.94 | 7.8 | 0.0185 | FASTmrEMMA | 2 | 52417495 | -0.0013 | 3.31 | 4.02 | 4.849 | pKWmEB |
| 11 | 48808618 | -0.0081 | 6.17 | 7.01 | 0.0218 | FASTmrEMMA | 3 | 6582830 | -0.0284 | 3.25 | 3.96 | 2.3517 | pKWmEB |
| 11 | 87229489 | -6.00E-04 | 4.35 | 5.12 | 3.43E-05 | FASTmrEMMA | 5 | 87945266 | 0.0266 | 5.06 | 5.86 | 1.3354 | pKWmEB |
| 11 | 103285126 | -0.0031 | 4.36 | 5.13 | 0.0034 | FASTmrEMMA | 7 | 98954416 | 0.0261 | 3.44 | 4.16 | 2.2349 | pKWmEB |
| 11 | 103289035 | -0.0058 | 3.43 | 4.16 | 0.0115 | FASTmrEMMA | 12 | 2694960 | -0.0225 | 3.23 | 3.94 | 3.7222 | pKWmEB |
| 11 | 105397592 | 0.0224 | 3.32 | 4.04 | 0.1571 | FASTmrEMMA | 15 | 68722924 | -0.0269 | 3.09 | 3.79 | 4.6118 | pKWmEB |
| 11 | 106085417 | -0.0017 | 4.11 | 4.86 | 2.00E-04 | FASTmrEMMA | 16 | 14557973 | 0.025 | 3.32 | 4.03 | 1.7466 | pKWmEB |
| 12 | 2693770 | -0.0034 | 6.54 | 7.39 | 0.0033 | FASTmrEMMA | 23 | 21336942 | 0.0174 | 3.25 | 3.96 | 5.0578 | pKWmEB |
| 12 | 2694960 | -0.005 | 4.22 | 4.98 | 0.006 | FASTmrEMMA | 1 | 9427932 | 0.0232 | 3.43 | 4.16 | 0.7465 | ISIS EM-BLASSO |
| 12 | 2706809 | -0.0044 | 3.71 | 4.45 | 0.0058 | FASTmrEMMA | 1 | 98943363 | -0.0291 | 4.29 | 5.06 | 1.359 | ISIS EM-BLASSO |
| 12 | 45303004 | -0.0019 | 3.99 | 4.74 | 5.00E-04 | FASTmrEMMA | 2 | 46721161 | -0.0342 | 6.5 | 7.35 | 2.3939 | ISIS EM-BLASSO |
| 12 | 52534782 | -2.00E-04 | 3.96 | 4.71 | 6.90E-07 | FASTmrEMMA | 3 | 32260522 | -0.0236 | 5.66 | 6.48 | 0.8113 | ISIS EM-BLASSO |
| 12 | 57008805 | 0.0032 | 3.28 | 4 | 0.0016 | FASTmrEMMA | 3 | 44060152 | 0.0255 | 4.85 | 5.64 | 1.4004 | ISIS EM-BLASSO |
| 12 | 78378824 | -0.0044 | 6.21 | 7.05 | 0.0065 | FASTmrEMMA | 3 | 79036558 | 0.0207 | 3.51 | 4.24 | 0.9599 | ISIS EM-BLASSO |
| 12 | 84524504 | 0.006 | 5.99 | 6.83 | 0.0085 | FASTmrEMMA | 3 | 120936971 | 0.0221 | 4.48 | 5.26 | 1.0596 | ISIS EM-BLASSO |
| 12 | 84530638 | 0.0046 | 9.96 | 10.89 | 0.0048 | FASTmrEMMA | 4 | 23622809 | -0.0231 | 4.79 | 5.57 | 1.0662 | ISIS EM-BLASSO |
| 13 | 2914469 | 0.0015 | 7.57 | 8.45 | 4.00E-04 | FASTmrEMMA | 4 | 82581345 | 0.0203 | 3.35 | 4.06 | 0.8658 | ISIS EM-BLASSO |
| 13 | 39533788 | 0.005 | 4.16 | 4.92 | 0.0068 | FASTmrEMMA | 4 | 101451691 | 0.0246 | 3.03 | 3.73 | 1.241 | ISIS EM-BLASSO |
| 13 | 59620161 | -0.0017 | 3.52 | 4.25 | 0.001 | FASTmrEMMA | 4 | 103251884 | 0.0252 | 5.87 | 6.7 | 1.3963 | ISIS EM-BLASSO |
| 13 | 60014103 | -7.00E-04 | 6.08 | 6.92 | 8.30E-06 | FASTmrEMMA | 5 | 19436069 | -0.0246 | 6.66 | 7.52 | 1.3361 | ISIS EM-BLASSO |
| 13 | 60574380 | 0.0066 | 6.89 | 7.75 | 0.0108 | FASTmrEMMA | 5 | 87945266 | 0.0279 | 8.29 | 9.19 | 0.7976 | ISIS EM-BLASSO |
| 13 | 72588925 | -0.0015 | 3.74 | 4.48 | 2.00E-04 | FASTmrEMMA | 5 | 120339577 | -0.0345 | 7.86 | 8.75 | 2.618 | ISIS EM-BLASSO |
| 14 | 49785058 | 7.00E-04 | 5.52 | 6.34 | 1.34E-05 | FASTmrEMMA | 7 | 98954416 | 0.0304 | 4.18 | 4.94 | 0.9864 | ISIS EM-BLASSO |
| 15 | 17658204 | -0.0026 | 3.28 | 3.99 | 0.0013 | FASTmrEMMA | 8 | 27592207 | -0.0243 | 6.9 | 7.76 | 1.15 | ISIS EM-BLASSO |
| 15 | 68722924 | -0.0024 | 3.43 | 4.15 | 7.00E-04 | FASTmrEMMA | 8 | 30093176 | -0.0179 | 3.44 | 4.16 | 0.3918 | ISIS EM-BLASSO |
| 16 | 1595222 | -0.0013 | 4.51 | 5.28 | 1.00E-04 | FASTmrEMMA | 10 | 57724034 | 0.028 | 4.67 | 5.45 | 0.1219 | ISIS EM-BLASSO |
| 16 | 11506312 | -0.002 | 5.27 | 6.08 | 0.001 | FASTmrEMMA | 10 | 60006026 | 0.0155 | 4.01 | 4.76 | 0.5108 | ISIS EM-BLASSO |
| 16 | 14813526 | -0.0089 | 4.81 | 5.6 | 0.022 | FASTmrEMMA | 10 | 87848389 | -0.0307 | 4.46 | 5.23 | 1.8986 | ISIS EM-BLASSO |
| 16 | 26192996 | 0.0055 | 6.78 | 7.64 | 0.0105 | FASTmrEMMA | 11 | 13638961 | 0.0134 | 3.07 | 3.77 | 0.3922 | ISIS EM-BLASSO |
| 16 | 55784564 | -0.005 | 3.54 | 4.26 | 0.0075 | FASTmrEMMA | 11 | 105557304 | 0.0261 | 3.65 | 4.38 | 1.1643 | ISIS EM-BLASSO |
| 16 | 58558880 | -0.0043 | 3.58 | 4.31 | 0.006 | FASTmrEMMA | 12 | 2694960 | -0.028 | 5.67 | 6.5 | 1.6648 | ISIS EM-BLASSO |
| 16 | 58565978 | -0.0043 | 3.68 | 4.42 | 0.0064 | FASTmrEMMA | 12 | 57008805 | 0.0294 | 4.53 | 5.3 | 1.4951 | ISIS EM-BLASSO |
| 16 | 61791136 | -0.0055 | 10.15 | 11.1 | 0.0077 | FASTmrEMMA | 13 | 10102104 | 0.0173 | 3.06 | 3.76 | 0.6406 | ISIS EM-BLASSO |
| 16 | 73840001 | 0.0024 | 11.64 | 12.61 | 0.0017 | FASTmrEMMA | 13 | 60570356 | 0.0217 | 3.38 | 4.1 | 0.9572 | ISIS EM-BLASSO |
| 16 | 73859947 | 3.00E-04 | 14.72 | 15.74 | 2.08E-05 | FASTmrEMMA | 14 | 61307574 | 0.0234 | 3.49 | 4.22 | 1.1752 | ISIS EM-BLASSO |
| 17 | 52566415 | -0.0024 | 7.11 | 7.98 | 0.001 | FASTmrEMMA | 15 | 42998912 | -0.0208 | 4.24 | 5.01 | 0.9563 | ISIS EM-BLASSO |
| 17 | 53620065 | 0.0018 | 6.04 | 6.87 | 0.001 | FASTmrEMMA | 15 | 61936585 | 0.0162 | 3.24 | 3.95 | 0.5773 | ISIS EM-BLASSO |
| 17 | 53755199 | 0.0014 | 4.16 | 4.92 | 4.00E-04 | FASTmrEMMA | 16 | 14813526 | -0.023 | 4.17 | 4.93 | 1.1822 | ISIS EM-BLASSO |
| 17 | 60241679 | -0.0054 | 7.38 | 8.26 | 0.0094 | FASTmrEMMA | 16 | 26192996 | 0.0182 | 3.91 | 4.65 | 0.5214 | ISIS EM-BLASSO |
| 18 | 38315068 | 0.0043 | 25.58 | 26.71 | 0.0057 | FASTmrEMMA | 17 | 19876360 | 0.0277 | 4.38 | 5.15 | 1.4099 | ISIS EM-BLASSO |
| 18 | 38317858 | 0.0075 | 32.76 | 33.95 | 0.0174 | FASTmrEMMA | 17 | 43400994 | 0.0211 | 3.78 | 4.52 | 0.8969 | ISIS EM-BLASSO |
| 18 | 39908826 | 0.0018 | 8.6 | 9.5 | 5.00E-04 | FASTmrEMMA | 17 | 51676255 | 0.0138 | 3.19 | 3.9 | 0.3373 | ISIS EM-BLASSO |
| 18 | 39961400 | 0.0014 | 6.2 | 7.04 | 3.00E-04 | FASTmrEMMA | 17 | 53620065 | 0.023 | 3.94 | 4.69 | 1.143 | ISIS EM-BLASSO |
| 18 | 51399522 | 0.0013 | 4.66 | 5.44 | 6.64E-05 | FASTmrEMMA | 18 | 41347362 | -0.0044 | 4.08 | 4.84 | 0.0325 | ISIS EM-BLASSO |
| 19 | 57385189 | -0.0019 | 8.68 | 9.58 | 3.00E-04 | FASTmrEMMA | 20 | 44864240 | 0.0062 | 4.67 | 5.45 | 0.0868 | ISIS EM-BLASSO |
| 20 | 24932795 | -0.0035 | 5.25 | 6.06 | 0.0021 | FASTmrEMMA | 21 | 30978657 | -0.0257 | 6.59 | 7.44 | 0.7558 | ISIS EM-BLASSO |
| 20 | 37391207 | -0.0017 | 8.67 | 9.57 | 4.00E-04 | FASTmrEMMA | 22 | 8023152 | 0.015 | 4.09 | 4.85 | 0.4859 | ISIS EM-BLASSO |
| 20 | 52784792 | -0.0021 | 3.32 | 4.04 | 6.00E-04 | FASTmrEMMA | 22 | 21951237 | -0.0145 | 3.01 | 3.7 | 0.3374 | ISIS EM-BLASSO |
| 21 | 30983811 | -0.0058 | 3.44 | 4.16 | 0.0114 | FASTmrEMMA | 24 | 27838377 | -0.0365 | 5.49 | 6.3 | 2.2718 | ISIS EM-BLASSO |
| 21 | 45748303 | -0.0024 | 3.14 | 3.84 | 0.001 | FASTmrEMMA | 25 | 16334342 | -0.026 | 5.57 | 6.39 | 1.3828 | ISIS EM-BLASSO |
| 21 | 45767354 | -0.002 | 3.6 | 4.33 | 5.00E-04 | FASTmrEMMA | 27 | 8476228 | 0.0157 | 4.71 | 5.49 | 0.4761 | ISIS EM-BLASSO |
| 22 | 19073643 | -9.00E-04 | 6.21 | 7.05 | 3.52E-05 | FASTmrEMMA | 27 | 22604390 | -0.0187 | 3.06 | 3.76 | 0.7701 | ISIS EM-BLASSO |
| 22 | 21953136 | 8.00E-04 | 4.37 | 5.14 | 2.00E-04 | FASTmrEMMA | 27 | 38286763 | -0.0327 | 8.73 | 9.64 | 0.8199 | ISIS EM-BLASSO |
| 22 | 43829445 | 0.0056 | 4.05 | 4.8 | 0.0087 | FASTmrEMMA | 27 | 43737541 | 0.0216 | 3.61 | 4.34 | 0.5826 | ISIS EM-BLASSO |
| 22 | 56906352 | 9.00E-04 | 3.46 | 4.18 | 7.32E-05 | FASTmrEMMA | 28 | 45890977 | 0.0226 | 4.85 | 5.64 | 0.9327 | ISIS EM-BLASSO |
| 22 | 57693714 | 0.0013 | 6.65 | 7.5 | 1.00E-04 | FASTmrEMMA | 29 | 49511085 | 0.0224 | 5.53 | 6.34 | 1.0817 | ISIS EM-BLASSO |
| 23 | 13428133 | 0.0076 | 14.6 | 15.62 | 0.0191 | FASTmrEMMA |  |  |  |  |  |  |  |
