# Supplementary material for: mrMLM v4.0.2: An R Platform for Multi-locus Genome-wide Association Studies
Source: Genomics Proteomics Bioinformatics. 2020 Dec 18;18(4):481–7. doi: 10.1016/j.gpb.2020.06.006 (PMC8242264; doi:10.1016/j.gpb.2020.06.006)
Supplement: Supplementary Table S8 — Previously reported genes for kidney weight in Simmental beef cattle around the QTNs identified by our multi-locus GWAS methods [file mmc18.docx]

**Table S8 Previously reported genes for kidney weight in Simmental beef cattle around the QTNs identified by our multi-locus GWAS methods**

| Chr | Position (bp) | Multi-locus GWAS | | | |  | Comparative genomics analysis | | |
| --- | --- | --- | --- | --- | --- | --- | --- | --- | --- |
|  |  | **QTN effect** | **LOD score** | **r^2^ (%)** | **Method** |  | **Candidate gene and its functional annotation** | **Distance (kb) ‡** | **PMID** |
| 1 | 98943363 | -0.0291 | 4.29 | 1.3590 | 6 |  | MDS1 and EVI1 complex locus; *MECOM* | 147 | 25950803 |
| 6 | 39387542 | 0.0026 | 12.0 | 0.0057 | 4 |  | non-SMC condensin I complex subunit G; *NCAPG* | 575 | 27892541 |
| 6 | 39410541 | 0.0031~0.0050 | 14.92~25.96 | 0.0011~0.0426 | 3,4 |  | ligand dependent nuclear receptor corepressor like; *LCORL* | 418 | 27892541 |

*Note:* †, 1, 2, 3, 4, 5, and 6 represent mrMLM, FASTmrMLM, FASTmrEMMA, pLARmEB, pKWmEB, and ISIS EM-BLASSO, respectively. ‡, distance (kb) between QTNs and gene.
