# Supplementary material for: mrMLM v4.0.2: An R Platform for Multi-locus Genome-wide Association Studies
Source: Genomics Proteomics Bioinformatics. 2020 Dec 18;18(4):481–7. doi: 10.1016/j.gpb.2020.06.006 (PMC8242264; doi:10.1016/j.gpb.2020.06.006)
Supplement: Supplementary Table S9 — Comparison of power (%), mean squared error (MSE), and false positive rate (FPR, %) for nine GWAS methods in the first simulation experiment [file mmc19.docx]

**Table S9 Comparison of power (%), mean squared error (MSE), and false positive rate (FPR, %) for nine GWAS methods in the first simulation experiment**

| Method^*^ | QTL_1_ | |  | QTL_2_ | |  | QTL_3_ | |  | QTL_4_ | |  | QTL_5_ | |  | QTL_6_ | | FPR(%) |
| --- | --- | --- | --- | --- | --- | --- | --- | --- | --- | --- | --- | --- | --- | --- | --- | --- | --- | --- |
|  | **Power** | **MSE** |  | **Power** | **MSE** |  | **Power** | **MSE** |  | **Power** | **MSE** |  | **Power** | **MSE** |  | **Power** | **MSE** |  |
| mrMLM | 92.6 | 0.0703 |  | 61.9 | 0.0663 |  | 48.7 | 0.1413 |  | 95.0 | 0.0867 |  | 42.0 | 0.1316 |  | 61.7 | 0.0638 | 0.0167 |
| FASTmrMLM | 95.5 | 0.0839 |  | 63.1 | 0.0533 |  | 48.9 | 0.1115 |  | 95.6 | 0.1092 |  | 35.4 | 0.0856 |  | 63.3 | 0.0465 | 0.0179 |
| FASTmrEMMA | 94.2 | 0.3111 |  | 63.9 | 0.2011 |  | 46.2 | 0.3516 |  | 97.9 | 0.4394 |  | 44.6 | 0.2952 |  | 66.7 | 0.1920 | 0.0080 |
| ISIS EBLASSO | 95.3 | 0.0897 |  | 58 | 0.0622 |  | 53.0 | 0.0930 |  | 97.8 | 0.1190 |  | 48.3 | 0.0784 |  | 66.6 | 0.0556 | 0.0322 |
| pLARmEB | 92.3 | 0.0821 |  | 61.8 | 0.0561 |  | 44.2 | 0.0930 |  | 94.8 | 0.1128 |  | 42.6 | 0.0927 |  | 65.6 | 0.0531 | 0.0195 |
| pKWmEB | 94.8 | 0.0898 |  | 66.1 | 0.0580 |  | 53.3 | 0.0943 |  | 95.6 | 0.1177 |  | 37.1 | 0.0801 |  | 68.3 | 0.0519 | 0.0326 |
| GEMMA | 72.8 | 0.2309 |  | 22.1 | 0.6634 |  | 24.2 | 0.7049 |  | 89.9 | 0.1766 |  | 35.6 | 0.9128 |  | 31.4 | 0.5704 | 0.0325 |
| EMMAX | 70.2 | 0.2284 |  | 20.4 | 0.6754 |  | 21.9 | 0.7091 |  | 85.8 | 0.1834 |  | 21.7 | 1.0052 |  | 28.9 | 0.5856 | 0.0117 |
| FarmCPU | 79.9 | 0.1457 |  | 57.2 | 0.0370 |  | 8.20 | 0.0590 |  | 69.2 | 0.2882 |  | 11.8 | 0.1297 |  | 53.1 | 0.0341 | 0.0116 |

*Note*: *, all the results were re-calculated using our mrMLM v4.0.2, including mrMLM, FASTmrMLM, FASTmrEMMA, ISIS EBLASSO, pLARmEB, and pKWmEB, which were published in the refs [16–21]. Note that the results of pLARmEB in the ref [20] aren’t consistent with those in the published paper, because there is one mistake in selecting potentially associated markers in the Monte Carlo simulation experiments of the ref [20]. The same is true for the later Tables.
