# Supplementary material for: mrMLM v4.0.2: An R Platform for Multi-locus Genome-wide Association Studies
Source: Genomics Proteomics Bioinformatics. 2020 Dec 18;18(4):481–7. doi: 10.1016/j.gpb.2020.06.006 (PMC8242264; doi:10.1016/j.gpb.2020.06.006)
Supplement: Supplementary Table S10 — Comparison of power (%), MSE, and FPR (%) for nine GWAS methods in the second simulation experiment [file mmc20.docx]

**Table S10 Comparison of power (%), MSE, and FPR (%) for nine GWAS methods in the second simulation experiment**

| Method^*^ | QTL_1_ | |  | QTL_2_ | |  | QTL_3_ | |  | QTL_4_ | |  | QTL_5_ | |  | QTL_6_ | | FPR(%) |
| --- | --- | --- | --- | --- | --- | --- | --- | --- | --- | --- | --- | --- | --- | --- | --- | --- | --- | --- |
|  | **Power** | **MSE** |  | **Power** | **MSE** |  | **Power** | **MSE** |  | **Power** | **MSE** |  | **Power** | **MSE** |  | **Power** | **MSE** |  |
| mrMLM | 94.8 | 0.0694 |  | 65.3 | 0.0481 |  | 39.4 | 0.0959 |  | 98.0 | 0.1176 |  | 29.3 | 0.0929 |  | 75.4 | 0.0850 | 0.0210 |
| FASTmrMLM | 93.9 | 0.1118 |  | 66.2 | 0.0664 |  | 40.6 | 0.0586 |  | 96.9 | 0.1107 |  | 23.1 | 0.0837 |  | 74.4 | 0.0566 | 0.0221 |
| FASTmrEMMA | 96.5 | 0.3620 |  | 72.8 | 0.2394 |  | 52.7 | 0.3342 |  | 98.0 | 0.4690 |  | 49.1 | 0.2961 |  | 70.8 | 0.2435 | 0.0077 |
| ISIS EBLASSO | 95.5 | 0.1187 |  | 61.8 | 0.0665 |  | 44.0 | 0.0662 |  | 99.0 | 0.1184 |  | 34.5 | 0.0641 |  | 78.6 | 0.0704 | 0.0408 |
| pLARmEB | 93.4 | 0.1098 |  | 66.2 | 0.0682 |  | 37.0 | 0.0638 |  | 98.1 | 0.1095 |  | 33.0 | 0.0615 |  | 74.9 | 0.0598 | 0.0247 |
| pKWmEB | 94.8 | 0.1202 |  | 72.1 | 0.0757 |  | 44.9 | 0.0634 |  | 98.2 | 0.1175 |  | 26.3 | 0.0568 |  | 74.6 | 0.0614 | 0.0400 |
| GEMMA | 71.7 | 0.1852 |  | 22.1 | 0.7121 |  | 8.50 | 0.6665 |  | 98.8 | 0.3111 |  | 16.6 | 0.9415 |  | 37.3 | 0.5915 | 0.0166 |
| EMMAX | 69.2 | 0.1857 |  | 20.2 | 0.7301 |  | 7.60 | 0.6704 |  | 98.4 | 0.3031 |  | 9.30 | 1.0748 |  | 34.6 | 0.5989 | 0.0051 |
| FarmCPU | 83.5 | 0.1120 |  | 67.5 | 0.0521 |  | 1.30 | 0.0426 |  | 93.7 | 0.1537 |  | 3.60 | 0.1950 |  | 59.5 | 0.0381 | 0.0119 |

*Note*: *, all the results were re-calculated using our mrMLM v4.0.2, including mrMLM, FASTmrMLM, FASTmrEMMA, ISIS EBLASSO, pLARmEB, and pKWmEB, which were published in the refs [16–21]. Note that the results of pLARmEB in the ref [20] aren’t consistent with those in the published paper, because there is one mistake in selecting potentially associated markers in the Monte Carlo simulation experiments of the ref [20]. The same is true for the later Tables.
