# Supplementary material for: mrMLM v4.0.2: An R Platform for Multi-locus Genome-wide Association Studies
Source: Genomics Proteomics Bioinformatics. 2020 Dec 18;18(4):481–7. doi: 10.1016/j.gpb.2020.06.006 (PMC8242264; doi:10.1016/j.gpb.2020.06.006)
Supplement: Supplementary Table S11 — Comparison of power (%), MSE, and FPR (%) for nine GWAS methods in the third simulation experiment [file mmc21.docx]

**Table S11 Comparison of power (%), MSE, and FPR (%) for nine GWAS methods in the third simulation experiment**

| Method^*^ | QTL_1_ | |  | QTL_2_ | |  | QTL_3_ | |  | QTL_4_ | |  | QTL_5_ | |  | QTL_6_ | | FPR(%) |
| --- | --- | --- | --- | --- | --- | --- | --- | --- | --- | --- | --- | --- | --- | --- | --- | --- | --- | --- |
|  | **Power** | **MSE** |  | **Power** | **MSE** |  | **Power** | **MSE** |  | **Power** | **MSE** |  | **Power** | **MSE** |  | **Power** | **MSE** |  |
| mrMLM | 93.0 | 0.0885 |  | 50.9 | 0.0548 |  | 40.6 | 0.1519 |  | 97.9 | 0.1118 |  | 24.4 | 0.1611 |  | 44.5 | 0.0507 | 0.0264 |
| FASTmrMLM | 92.6 | 0.1216 |  | 52.7 | 0.0770 |  | 43.2 | 0.0858 |  | 97.0 | 0.1443 |  | 19.7 | 0.0852 |  | 45.9 | 0.0820 | 0.0275 |
| FASTmrEMMA | 93.3 | 0.4450 |  | 50.9 | 0.2464 |  | 29.5 | 0.3030 |  | 98.0 | 0.5331 |  | 31.6 | 0.2827 |  | 46.9 | 0.2799 | 0.0138 |
| ISIS EBLASSO | 94.9 | 0.1323 |  | 52.4 | 0.0804 |  | 44.6 | 0.0794 |  | 99.4 | 0.1553 |  | 32.0 | 0.0788 |  | 53.1 | 0.0892 | 0.0477 |
| pLARmEB | 91.6 | 0.1196 |  | 53.6 | 0.0747 |  | 38.6 | 0.0835 |  | 97.1 | 0.1433 |  | 28.5 | 0.0598 |  | 48.3 | 0.0795 | 0.0296 |
| pKWmEB | 93.3 | 0.1359 |  | 59.1 | 0.0757 |  | 45.3 | 0.0893 |  | 98.4 | 0.1654 |  | 20.1 | 0.0722 |  | 52.3 | 0.0966 | 0.0480 |
| GEMMA | 72.9 | 0.2044 |  | 15.4 | 0.7576 |  | 16.0 | 0.7218 |  | 93.8 | 0.2131 |  | 19.7 | 1.0254 |  | 16.7 | 0.6615 | 0.0253 |
| EMMAX | 71.6 | 0.2031 |  | 14.4 | 0.7716 |  | 12.6 | 0.7415 |  | 92.1 | 0.2160 |  | 8.70 | 1.2131 |  | 15.9 | 0.6732 | 0.0091 |
| FarmCPU | 78.3 | 0.0981 |  | 47.8 | 0.0534 |  | 5.10 | 0.1065 |  | 76.5 | 0.1709 |  | 5.00 | 0.1899 |  | 40.6 | 0.0487 | 0.0161 |

*Note*: *, all the results were re-calculated using our mrMLM v4.0.2, including mrMLM, FASTmrMLM, FASTmrEMMA, ISIS EBLASSO, pLARmEB, and pKWmEB, which were published in the refs [16–21]. Note that the results of pLARmEB in the ref [20] aren’t consistent with those in the published paper, because there is one mistake in selecting potentially associated markers in the Monte Carlo simulation experiments of the ref [20]. The same is true for the later Tables.
